# Deciphering the intracellular metabolism of *Listeria monocytogenes *by mutant screening and modelling

**DOI:** 10.1186/1471-2164-11-573

**Published:** 2010-10-18

**Authors:** Kristina Schauer, Gernot Geginat, Chunguang Liang, Werner Goebel, Thomas Dandekar, Thilo M Fuchs

**Affiliations:** 1 Zentralinstitut für Ernährungs- und Lebensmittelforschung (ZIEL), Abteilung Mikrobiologie, Technische Universität München, Weihenstephaner Berg 3, 85354 Freising, Germany; 2 Institut für medizinische Mikrobiologie und Hygiene, Fakultät für Medizin Mannheim der Universität Heidelberg, Theodor-Kutzer-Ufer 1-3, 68167 Mannheim, Germany; 3 Theodor-Boveri-Institut (Biozentrum), Abteilung Bioinformatik, Universität Würzburg, 97074 Würzburg, Germany; 4 Max von Pettenkofer-Institut für Hygiene und medizinische Mikrobiologie, Ludwig Maximilians-Universität München, Pettenkoferstr. 9a, 80336 München, Germany

## Abstract

**Background:**

The human pathogen *Listeria monocytogenes *resides and proliferates within the cytoplasm of epithelial cells. While the virulence factors essentially contributing to this step of the infection cycle are well characterized, the set of listerial genes contributing to intracellular replication remains to be defined on a genome-wide level.

**Results:**

A comprehensive library of *L. monocytogenes *strain EGD knockout mutants was constructed upon insertion-duplication mutagenesis, and 1491 mutants were tested for their phenotypes in rich medium and in a Caco-2 cell culture assay. Following sequencing of the plasmid insertion site, 141 different genes required for invasion of and replication in Caco-2 cells were identified. Ten in-frame deletion mutants were constructed that confirmed the data. The genes with known functions are mainly involved in cellular processes including transport, in the intermediary metabolism of sugars, nucleotides and lipids, and in information pathways such as regulatory functions. No function could be ascribed to 18 genes, and a counterpart of eight genes is missing in the apathogenic species *L. innocua*. Mice infection studies revealed the *in vivo *requirement of IspE (Lmo0190) involved in mevalonate synthesis, and of the novel ABC transporter Lmo0135-0137 associated with cysteine transport. Based on the data of this genome-scale screening, an extreme pathway and elementary mode analysis was applied that demonstrates the critical role of glycerol and purine metabolism, of fucose utilization, and of the synthesis of glutathione, aspartate semialdehyde, serine and branched chain amino acids during intracellular replication of *L. monocytogenes*.

**Conclusion:**

The combination of a genetic screening and a modelling approach revealed that a series of transporters help *L. monocytogenes *to overcome a putative lack of nutrients within cells, and that a high metabolic flexibility contributes to the intracellular replication of this pathogen.

## Background

*Listeria monocytogenes *is a gram-positive, food-borne pathogen which is able to grow at low temperatures down to 1.7°C [1]. It is widely distributed in nature and mainly affects immunocompromised individuals. A hallmark of this facultative intracellular pathogen is its capability to use eukaryotic cells as a predominant growth niche [2].

*L. monocytogenes *entry into epithelial cells is mediated by several cell surface proteins including internalin A and B [3]. The phagosomal membrane is then disrupted by the activity of a hemolysin and phospholipases, resulting in the release of *L. monocytogenes *into the cytosol of its host cell where it encounters an environment of undefined composition. There, the pathogen is able to move intra- and intercellular by expressing ActA which polymerizes host actin molecules. The genes required for these steps of the infection cycle are under control of the central transcription regulator PrfA [4]. While these virulence factors have been extensively studied in the last years, far less is known about the availability of nutrients in the cytosol of eukaryotic cells, and the specific metabolic adaptations of *L. monocytogenes *including substrate acquisition that allow its efficient proliferation within this cellular compartment [2,5,6]. The knowledge of the gene set involved in the multiplication of *L. monocytogenes *inside epithelial cells might also help to discover its metabolic Achilles' heel as a prerequisite to control its intracellular lifestyle and to combat this medically important pathogen.

In a pilot study on virulent strains or mutants of *L. monocytogenes *auxotrophic for amino acids and nucleobases, it was revealed that hosts can provide sufficient organic and inorganic compounds to overcome selected auxotrophies with respect to uracil, phenylalanine, glycine, proline, or nicotinic acid [7]. In contrast, the synthesis of all three aromatic amino acids and adenine is essential for efficient cytosolic replication in J774A.1 macrophage cells. Some further factors involved in nutrient uptake and metabolism have also been shown to be necessary for intracellular survival and proliferation of pathogenic *L. monocytogenes*. By screening a library of Tn917-*lac *insertion mutants, the specific induction of genes for purine and pyrimidine biosynthesis as well as for arginine uptake in macrophages was observed [8]. Reduced replication has been demonstrated for a mutant of the PrfA-dependent gene *hpt *whose product is involved in the exploitation of hexose phosphate from the host cell [9]. The use of host-derived lipoic acid has also been shown to be required for intracellular replication and virulence of *L. monocytogenes*. A mutant lacking the lipoate protein ligase LplA1 was less virulent in the mouse model, probably due to a loss of pyruvate dehydrogenase function whose E2 subunit is modified by LplA1 [10]. In two independent transcriptomic approaches following infection of macrophages and epithelial cells, it was demonstrated that the pentose phosphate cycle, but not glycolysis is the predominant pathway of listerial metabolism in the cellular host environment [11,12]. Induced synthesis of the branched chain amino acids and the utilization of alternative carbon sources such as glycerol were also observed in these studies. The absence of the Entner-Doudoroff pathway and a bifurcated citric acid cycle due to the lack of α-ketoglutarate dehydrogenase activity were predicted by genome analysis and then experimentally demonstrated [13,14]. Little is known about the *in vivo *nitrogen metabolism of *L. monocytogenes *that is assumed to use ammonium, arginine or ethanolamine as an alternative nitrogen source during intracellular replication [12]. Results with amino acids-deprived tissue culture cells and a *L. monocytogenes *mutant auxotrophic for threonine indicate that this pathogen may utilize intracellular peptides as a source of amino acids [7]. This assumption is supported by *oppA *encoding an oligopeptide permease that is involved in intra-macrophagic survival, indicating that the efficient uptake of oligopeptides favours growth of *L. monocytogenes *in macrophages [15]. Listerial growth within eukaryotic cells also depends on vitamins. Both the uptake of thiamine and the *de novo *biosynthesis of its precursors have recently been shown to contribute to intracellular proliferation, a finding that reflects the role of thiamine as a cofactor of enzymes involved in central metabolic processes such as the pentose-phosphate cycle or the synthesis of branched chain amino acids [16]. Furthermore, the listerial genome harbours the genes for cobalamine biosynthesis required for intracellular ethanolamine degradation [12,17].

To obtain an overview of genes required by *L. monocytogenes *to efficiently replicate in mammalian cells, we established a mutant library of *L. monocytogenes *by insertion duplication mutagenesis (IDM) and screened 1491 mutants for impaired capacity to invade or replicate within Caco-2 cells. A total of 141 mutants were isolated, most of them involved in metabolism, in transport and in cell wall functions. All mutants showed wildtype-like growth in nutrient-rich medium, indicating the compartment-specific role of the genes tested. Mathematical modelling then allowed to improve our understanding of the complex data obtained. The overall results shed further light on the set of listerial genes required for the replication in the cytosol of epithelial cells, and on the environment that *L. monocytogenes *encounters within host cells.

## Results

### 141 genes were identified that affect cell invasion or intracellular replication

For the identification of insertional mutations that affect the ability of *L. monocytogenes *EGD to proliferate within eukaryotic cells, 1491 *L. monocytogenes *insertion mutants were screened for a phenotype in a Caco-2 cell infection assay. 238 mutants that exhibited invasion defects or reduced intracellular replication rates in at least three independent experiments were identified. As the insertion of pLSV101 into the chromosome leads to slight growth deficiencies, the intracellular growth data of a random group of more than 100 IDM mutants were collected [12], and an at least two-fold attenuation with respect to the average data of the control group was set as a threshold. All mutants were then grown in BHI medium to exclude unspecific growth deficiencies, and only mutants exhibiting wildtype-like growth were considered for further analysis. Sequence analysis revealed that in these mutants, 141 different gene loci had been targeted by IDM (Table 1). The remaining mutants carried insertions of identical fragments or fragments of genes that had already been identified. As indicated in Table 1, 23 of the genes identified have recently been described due to the fact that their transcription is upregulated during replication of *L. monocytogenes *in Caco-2 cells or macrophages, while the expression of 19 other genes is reduced in these eukaryotic cells [11,12].

**Table 1:** Genes identified to be required for intracellular replication of *L. monocytogenes *within epithelial cells

			fold reduction					fold reduction	
							
gene name or number	protein name, or protein homology/similarity to	polar effect	**intrac**.	invasion/adhesion	gene name or number	protein name, or protein homology/similarity to	polar effect	**intrac**.	invasion/adhesion
**cell wall**					**protein modification**				
^1^lmo0441	penicillin-binding protein (D-alanyl-D-alanine carboxypeptidase)	no	9.4	6.1	^5^lmo0618	protein kinase	no	11.0	2.0
^2,8^lmo1085	teichoic acid biosynthesis protein B	rf	12.3	2	^1^lmo0763	hypothetical Ser/Thr protein phosphatase family protein	possible	5.5	2.0/5.0
lmo1088	teichoic acid biosynthesis protein B	rf	26.2	1.9	**metabolism of amino acids and related molecules**				
^7^lmo1713	actin-like ATPase involved in cell morphogenesis	no	3.6	3.5	lmo0594	homoserine O-acetyltransferase	no	7.7	3.9
^3,5,7^*pbpB *(lmo2039)	penicillin-binding protein 2B	rf	3.8	2.6	^6,8^lmo1235	aspartokinase II a subunit	no	5.9	nd
^1^lmo2555	Glycosyltransferase	rf	8.1	3.9/5.0	lmo1495	5'-methylthioadenosine/S-adenosylhomocysteine nucleosidase	possible	6.2	1.0
**transport/binding proteins and lipoproteins**					lmo1916	peptidase	no	7.0	2.4
^4^lmo0135 (*ctpA*)	oligopeptide ABC transport system, substrate binding protein	rf	13.6	2.2	^8^*aroB *(lmo1927)	3-dehydroquinate synthase	rf	2.8	1.4
^4^lmo0136	oligopeptide ABC transport system, substrate binding protein	rf	5.0	2.4	^4,6^*ilvB *(lmo1484)	acetolactate synthase (acetohydroxy-acid synthase)	rf	5.5	1.8
lmo0137	oligopeptide ABC transport system, permease	no	9.7	1.9	^4,6^*ilvC *(lmo1486)	ketol-acid reductoisomerase	no	3.2	3
^4^lmo0195	ABC-type antimicrobial peptide transport system, permease	no	6.3	nd	lmo2051	weakly similar to proteases	no	4.1	1
lmo0495	permease of the drug/metabolite transporter (DMT) superfamily	possible	4.2	2.3	lmo2694	lysine decarboxylase	possible	5.5	0
^4^lmo0584	conserved hypothetical membrane protein, putative permease	no	7.2	3.0	lmo2770	γ-glutamylcysteine synthetase and cyanophycin synthetase	no	5.8	2.3
lmo0645	amino acid transporter	no	4.9	1.5	^6^*serC *(lmo2825)	phosphoserine aminotransferase	possible	5.7	3.5
^5^lmo0650	conserved membrane protein	possible	2.8	2.0	**metabolism of nucleotides and nucleic acids**				
lmo0787	amino acid transporter	no	3.4	1.0	^1,5^*purA *(lmo0055)	adenylosuccinate synthetase	no	14.9	/4.0
lmo0810	spermidine/putrescine-binding protein	no	7.4	3.1	*purQ *(lmo1769)	phosphoribosylformylglycinamidine synthetase	rf	12.0	nd
^3^lmo1003	phosphotransferase system enzyme I	no	3.4	1.0	^8^*purS *(lmo1771)	phosphoribosylformylglycinamidine synthetase	rf	11.0	1.0
*gbuA *(lmo1014)	glycine betaine ABC transporter, ATP-binding protein	rf	10.0	4.0	*pyrE *(lmo1831)	orotatephosphoribosyltransferase	no	5.5	2.5
^8^lmo1416	hypothetical transporter	no	5.3	1.0	**metabolism of lipids**				
^6^*opuCA *(lmo1428)	glycine betaine/carnitine/choline ABC transporter, ATP-binding protein	rf	15.7	1.7	^5,8^*ispE *(lmo0190)	CDP-ME synthase involved in isoprenoid biosynthesis	no	4.7	2.3
^5^lmo1431	ABC transporter, ATP binding protein	no	4.8	3	lmo1005	3-hydroxyisobutyrate dehydrogenase	no	11.0	1.8
lmo1506	ABC-type antimicrobial peptide transport system, permease	rf	6.2	2.3	lmo1363	geranyltransferase	no	5.0	2.8
lmo1739	amino acid ABC transporter	rf	5.5	2	lmo2450	carboxylesterase	possible	4.2	3.0
^3,5,7,8^lmo1847	ABC transporter specific for metal cations	no	5.8	2.3	**metabolism of coenzymes and prosthetic groups**				
lmo2124	maltodextrin ABC transport system, permease	rf	3.4	0	^5^lmo0221	hypothetical type III pantothenate kinase	possible	6.9	1.0
^1,3^*oppF *(lmo2192)	oligopeptide ABC-transporter, ATP-binding protein	no	21.0	8.0/4.0	^1,3^*pdxK *(lmo0662)	pyridoxine kinase	no	13.1	1/4.0
lmo2227	ABC transporter, ATP-binding protein	possible	5.8	1.3	lmo1043	molybdopterin-guanine dinucleotide biosynthesis MobB	rf	7.9	1.4
lmo2249	low-affinity inorganic phosphate transporter	no	2.8	2.0	lmo1932	heptaprenyl diphosphate synthase component I	rf	12.1	4.8
lmo2353	hypothetical Na+/H+ antiporter	no	4.3	2.3	*nadB *(lmo2023)	L-aspartate oxidase	rf	3.4	0
lmo2380	protein involved in resistance to cholate/Na^+ ^and in pH homeostasis	rf	5.7	2.5	^3^lmo2102	glutamine amidotransferase subunit PdxT (pyridoxine biosynthesis)	no	4.0	3.3
lmo2430	*B. subtilis *ferrichrome ABC transporter FhuG, permease	rf	75.6	5.0	lmo2566	biotin/lipoate A/B protein ligase family	possible	6.2	2.0
lmo2816	transport protein	no	6.3	2.0	**DNA restriction/modification and repair**				
lmo2850	sugar transport protein	rf	16.5	0	^5^*gyrB *(lmo0006)	DNA gyrase subunit B	rf	9.6	nd
**sensors**					lmo0157	ATP-dependent helicase	possible	2.1	1.0
lmo0799	oxygen/light sensor with PAS domain	no	8.3	2.0	*mfd *(lmo0214)	transcription-repair coupling factor	possible	8.9	3.7
lmo1508	two-component sensor histidine kinase	no	5.2	2.9	lmo0588	DNA photolyase	no	9.6	2.0
**membrane bioenergetics**					*mutM *(lmo1564)	formamidopyrimidine-DNA glycosylase	no	5.2	1.1
lmo0091	ATP synthase g chain, H^+^-transporting two-sector ATPase	rf	5.7	2.0	lmo1751	hypothetical RNA methyltransferase	no	6.2	2.0
*nifJ *(lmo0829)	pyruvate-flavodoxin oxidoreductase	no	6.4	3.6	^8^*ansB *(lmo1663)	asparaginyl-tRNA synthetases	no	4.1	1.6
^5^*atpA *(lmo2531)	H+-transporting ATP synthase chain α	rf	7.7	2.7	lmo2050	exconuclease ABC (subunit A)	no	5.1	2.0
*atpB *(lmo2535)	H+-transporting ATP synthase chain β	rf	4.4	5.9	**DNA recombination**				
**mobility and chemotaxis**					^6^*recN *(lmo1368)	RecN	no	14.8	1.9
lmo0680	flagella-associated protein FlhA	rf	7.0	3.1	*ruvB *(lmo1532)	Holliday junction DNA helicase RuvB	possible	9.4	3.9
^6^lmo0700	flagellar motor switch protein FliY	rf	5.8	1.7	^8^*recS *(lmo1942)	similar to ATP-dependent DNA helicase	no	7.0	2.3
**cell surface proteins**					**regulation**				
^1,2,6^*vip *(lmo0320)	putative peptidoglycan bound protein with LPXTG motif	no	6.4	4.5	^3^*agrA *(lmo0051)	2-component response regulator protein	no	3.4	1.0
^8^lmo0327	protein with LPXTG motif, putative murein hydrolase activity	no	6.9	3.0	^6^lmo0294	transcription regulator, LysR-*gltR *family	no	7.4	3.1
^4^lmo0514	internalin-like protein with LPXTG motif	no	6.4	1.4	lmo0535	transcription regulator, LacI family	no	9.5	1.0
^4^lmo0576	hypothetical cell wall associated protein	no	4.2	1.5	^3^*fur *(lmo1956)	transcriptional regulator, Fur family	no	5.5	1.0
^2,8^lmo1666	peptidoglycan linked protein with LPXTG motif	no	6.8	1.8	lmo1994	transcription regulators, LacI family	no	9.0	1.4
lmo2026	hypothetical peptidoglycan bound protein with LPXTG motif	no	8.7	1.4	**RNA modification**				
^5^lmo2504	cell wall binding protein, peptidase-related enzyme	no	4.8	2.0	lmo0241	hypothetical RNA methyltransferase, *trmH *family protein	rf	4.1	1.0
^1^*ami *(lmo2558)	autolysin, N-acetylmuramoyl-L-alanine amidase	no	3.8	5.3	^1,3,5^lmo1434	RNA-metabolising metallo-β-lactamase	no	9.6	3/22
**metabolism of carbohydrates and related molecules**					**miscellaneous**				
*glpQ *(lmo0052)	transmembrane protein with phosphoesterase domain	possible	4.1	2.0	lmo0066	toxin component of A/B toxin	rf	2.8	1.0
^8^lmo0182	α-xylosidase and α-glucosidase	rf	7.9	1.3	^4^lmo0585	secreted protein	no	4.8	1.0
lmo0261	phospho-β-glucosidase	no	3.4	0	lmo0587	secreted protein, YapH from *Y. pestis*, cell wall surface protein	no	3.4	1.0
^8^lmo0271	phospho-β-glucosidase	possible	4.1	1.0	**unknown proteins**				
lmo0517	phosphoglycerate mutase	no	13.8	2.3	lmo0276	hypothetical hydrolase, HAD superfamily	possible	5.2	1.3
^2^lmo1031	hypothetical L-fucose isomerase	rf	11.1	2.1	^2^lmo0313	hypothetical hydrolase, PHP superfamily	no	2.8	1.0
^2^lmo1032	Transketolase	rf	14.4		^4^lmo0590	hypothetical DAK2/DegV domain-containing protein	possible	9.6	1.0
lmo1166	NADPH-dependent butanol dehydrogenaseI	possible	4.2	1.0	lmo0765	unknown protein	possible	10.4	3.0
^4^*glpD *(lmo1293)	glycerol-3-phosphate dehydrogenase	no	4.8	1.0	^4,6,7^lmo0794	*B. subtilis *YwnB protein	no	12.4	2.0
^6^lmo1244	weakly similar to phosphoglycerate mutase 1	no	8.3	2.0	lmo1379	*B. subtilis *SpoIIIJ protein, hypothetical membrane protein	no	4.8	3.0
lmo2005	Oxidoreductase	no	7.4	3.9	^3^lmo1402	*B. subtilis *YmcA protein	no	2.1	2.0
lmo2015	α-mannosidase	no	6.4	1.7	lmo1575	phosphoesterase, DHH superfamily	no	n.d.	1.0
lmo2134	fructose-1,6-biphosphate aldolase type II	no	5.2	2.4	^6,8^lmo1700	unknown protein	no	4.6	1.3
lmo2172	propionate CoA-transferase	possible	5.3	1.3	^4^lmo1830	short chain dehydrogenase	no	11.7	2.0
lmo2247	Oxidoreductase	possible	5.0	2.4	lmo1866	hypothetical phosphotransferase	possible	7.1	2.2
^8^lmo2446	Glycosidase	no	9.9	2.5	lmo1920	unknown protein		16.5	1.0
lmo2586	formate dehydrogenase α-chain	possible	2.4	2.2	lmo2516	conserved hypothetical protein	possible	3.6	1.2
^6^lmo2660	Transketolase	rf	3.0	2.6	lmo2639	unknown protein, contains DUF1312 domain	possible	5.5	1.0
lmo2764	xylose operon regulatory protein and to glucose kinase	rf	3.6	2.1	**no similarity**				
^2^lmo2781	β-glucosidase	rf	6.5	2.0	lmo0729	no similarity	no	4.8	1.0
lmo2831	Phosphoglucomutase	no	5.3	2.8	^2^lmo1188	no similarity	no	5.1	3.5
					lmo1219	no similarity	no	4.8	2.0
					lmo2129	no similarity	no	5.8	1.6

^1^adhesion (seven genes) or invasion (four genes) defective as determined 35 min or 2 h post infection, respectively; ^2^genes without homologue in *L. innocua*; genes downregulated^3 ^or upregulated^4 ^during growth in macrophages [11]; genes downregulated^5 ^or upregulated^6 ^during growth in Caco-2 cells [12]; ^7^induced *in vivo *[23]; ^8^*prfA *binding box is located upstream [13]. Fold reduction of intracellular (intrac.) replication in comparison to a control group of >100 insertion mutants [12]. rf, related function of downstream genes. Four genes from categories which one gene only (1.6 protein secretion, 1.7 cell division, 1.10 transformation, and 3.7 protein synthesis) was assigned to are not shown. See also Additional file 6 for generation times.

### Validation by in-frame deletions

**Figure 1:**
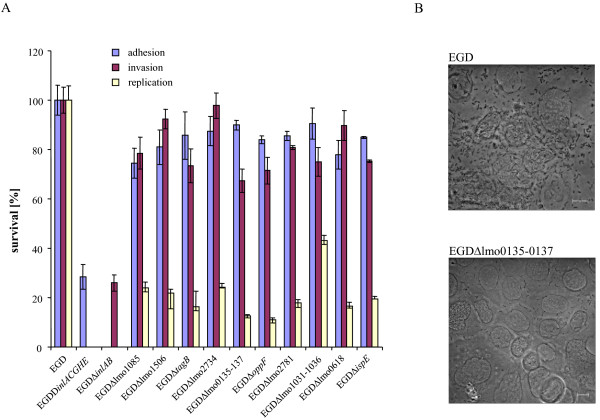
**Intracellular replication of *L. monocytogenes *deletion mutants**. (A) Caco-2 cells were infected at a multiplicity of infection (MOI) with either the wildtype strain or in-frame deletion mutants. The mutants EGDΔ*inlACGHE *and EGDΔ*inlAB *served as controls for the adhesion and invasion properties of the mutants. The number of viable bacterial cells recovered from the epithelial cells 8 h after infection was determined, and reduced survival of the ten mutants was calculated as a percentage in comparison with the wildtype strain. Error bars show the standard deviations from the mean. Each experiment was performed independently at least three times. The significance level was < 0.05 according to student's t test. (B) Phase contrast microscopy of a Caco-2 cell layer 8 h after infection with the wildtype strain (top) and mutant EGDΔlmo0135-0137 (buttom). The scale bars corresponds to 10 μm. The deletion of the transporter operon results in an approximately 10-fold reduced number of *L. monocytogenes *cells.

Although IDM has the potential for termination-induced reduction of downstream gene expression [18], a real-time RT-PCR approach revealed that regardless of its orientation, plasmid pLSV101 insertion did not significantly affect the transcription of the most distantly located genes of the operons investigated [12]. Moreover, genes clustered in an operon are often involved in the same cellular pathway or function (Table 1). To nevertheless further validate the data obtained, a series of ten non-polar deletion mutants were constructed. With the exception of lmo0618 expressing a putative protein kinase, genes selected for deletion encode proteins that belong to the classes of cell wall biosynthesis, metabolism, and transport. In all but one case, a five- to tenfold reduction in levels of intracellular replication within Caco-2 cells in comparison to the wildtype strain was observed (Figure 1A). EGDΔlmo1031-1036 showed a more modest reduction of replication, thus confirming the result of a recent study on listerial glycerol metabolism [19]. EGDΔlmo0135-0137 is an example for a very strong attenuation of the intracellular proliferation rate (Figure 1B). The experiments were also performed with an MOI of approximately 100, resulting in similar intracellular attenuation rates (data not shown). Taken together, each of the in-frame deletions led to intracellular growth attenuation, thus confirming the mutagenesis strategy applied.

### Classification of listerial genes required for invasion of and replication in Caco-2 cells

**Figure 2:**
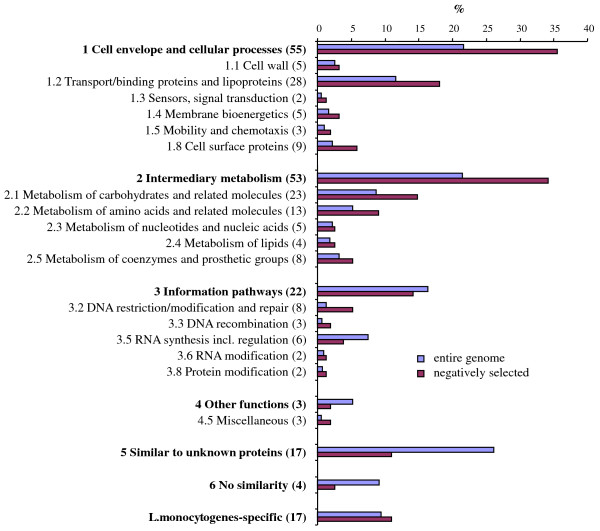
**Distribution of 157 genes negatively selected within different functional categories**. Classification and categorization of genes was done essentially in accordance with Glaser *et al. *[13] and the *L. monocytogenes *genome sequence provided by the Pasteur Institute http://genolist.pasteur.fr/ListiList/. Categories which no gene or one gene only (1.6 protein secretion, 1.7 cell division, 1.10 transformation, 3.7 protein synthesis) was assigned to were not listed, but the genes are included in total numbers (bold letters). For each category, the percentage of gene numbers relative to the total gene number in the *L. monocytogenes *genome (blue) and the number of genes with a Caco-2 infection phenotype relative to the total number of genes selected (red) is shown. For each class and category, the number of genes identified is indicated in brackets. 16 genes only recently identified [12] are included.

All genes were classified into functional categories (Figure 2, Table 1). 18 insertions into functional unknown genes were identified. Another striking feature of the classification is that 27 of the mutated genes belong to the class of transporters and lipoproteins, among them several uptake systems for sugars. At least five of the transporters isolated are possibly involved in the uptake of amino acids and oligopeptides (Table 1). Two of a total of ten antimicrobial permeases encoded by the listerial genome were also identified. A huge set of 47 genes are involved in intermediary metabolism, among them 21 genes contributing to the utilization of carbohydrates such as glucose, glycerol, or fucose, and 11 to the metabolism of amino acids (Table 1). Interestingly, genes of the two groups cell envelope/cellular processes including transport (35%) and intermediary metabolism (34%) are overrepresented, and genes of unknown function (11%) underrepresented with respect to their quota in the entire genome (22%, 22%, and 26%, Figure 2).

Eight genes identified in the screening described here have no homologue in the apathogenic species *L. innocua *(Table 1), indicating that their products might specifically contribute to virulence properties of *L. monocytogenes*. This group includes one *L. monocytogenes*-specific gene (lmo1188), lmo1085 required for teichoic acid synthesis, and several metabolic genes such as lmo2781 encoding a glucosidase (see below) or lmo1031 possibly involved in fucose utilization.

### Genes involved in invasion of epithelial cells, and cell surface genes

To discriminate between factors playing a role during intracellular multiplication from those that contribute to Caco-2 cell adhesion or invasion, the number of viable surface-attached or intracellular *L. monocytogenes *cells 35 minutes or two hours after infection was determined for all mutants selected above. Only mutants affected in their adhesion or invasion capabilities are expected to be identified by this approach. As bacterial entrance is a continuous process and cannot strictly be separated from the intracellular replication phase, only four mutants with an at least five-fold reduced number of intracellular bacteria two hours post infection were classified as invasion deficient. Further analysis of the plasmid insertion site revealed *ami*, lmo0441 encoding a putative D-alanyl-D-alanine carboxypeptidase, *oppF *encoding a membrane protein with similarity to an ABC transporter, and *vip *(lmo0320) (Table 1).

### Modelling

**Figure 3:**
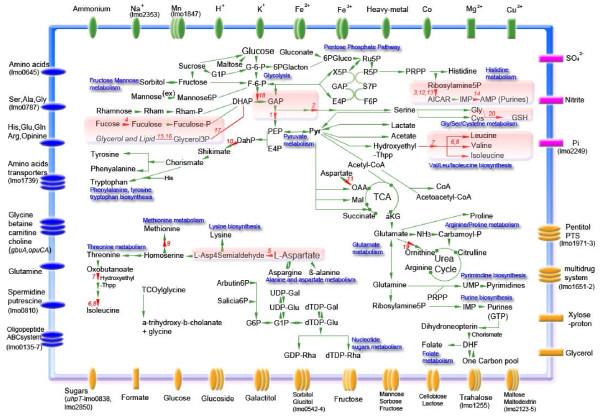
**Modelling of the listerial metabolism during intracellular replication**. The main anabolic and catabolic pathways of *L. monocytogenes *are shown. Transporters for cations (green), anions (pink), carbohydrates and organic acids (orange), and amino acids and peptides (blue) are depicted in the membrane. Numbers in red indicate key affected enzymes, areas shaded in light red key pathways for intracellular survival according to this study. Transporters are generally referred to TransportDB [56] and the Transport Classification Database tcdb [57], their known specificities were discussed [58]. Note the incomplete TCA pathway and a short-cut in the urea cycle [13,14]. Abbreviations: AICAR, 5-amino-1-(5-phospho-D-ribosyl)-imidazole-4-carboxamide; E4P, erythrose-4-phosphate; DHF, dihydrofolate; G-1-P, glucose-1-phosphate; DahP, 3-deoxy-D-arabino-heptulosonate-7-phosphate; F-6-P, fructose-6-phosphate; G-6-P, glucose-6-phosphate; GAP, glyceraldehyde-3-phosphate; OAA, oxaloacetate; aKB, 2-oxoglutarate; 6PGluco, 6-phosphogluconate; PRPP, 5-phosphoribosyl 1-pyrophosphate; R5P, ribose-5-phosphate; Ru5P, ribulose-5-phosphate; S7P, sedaheptulose-7-phosphate; TCA, tricarboxylic acid; TCOylcine, 3α,7α,12α-trihydroxy-5β-cholan-24-oylglycine; X5P, xylulose-5-phosphate; L-Asp4Semialdehyde, L-Aspartate 4-semialdehyde; GSH, glutathione.

The main anabolic and catabolic pathways of *L. monocytogenes *such as glycolysis, starch degradation, the pentose phosphate pathway, the tricarboxylic acid cycle (TCA), the metabolism of lipids and nucleic acids, and the biosynthesis of essential amino acids were reconstructed as described above, including consideration of important transporters (Figure 3). Upon data mining, additional genes were found in the literature to play a role during intracellular replication. 31 of them were further considered due to the availability of quantitative data (see Additional file 1 for list of genes and references). From Table 1 and Additional file 1, 20 genes where selected whose mutation resulted in an at least 3.2-fold reduction of the intracellular replication rate (see Additional file 2 for list of genes) and used for a simulation of the listerial metabolism during intracellular replication (see Additional file 3 for input data of the model for calculation with flux balance analysis).

Flux balance analysis [20] was applied to identify pathways critical for intracellular survival. Flux balance analysis assumes a steady state for all metabolites internal to the system. Given all biochemical reactions in a network, for each flux pathway in the network an equilibrium condition for each internal metabolite used by the different enzymes of this pathway has to be satisfied. Solving this balancing condition for all biochemical reactions in the network, the so called stochiometric matrix, directly enumerates all possible stable pathways of the system allowing equilibrium for their respective internal metabolites. Two types of calculation are possible: (i) All possible pathways in the system which satisfy the balancing condition and cannot be split further. These are used for elementary mode analysis (EMA). (ii) The extreme pathway analysis (EPA): a minimal set of pathways (a subset derived from EMA) that can completely describe the whole system by linear combinations of these fewer EPA pathways. They are termed extreme pathways because unique use of one of these pathways marks a boundary or extreme situation of the complete space of possibilities. While EPA is faster, one looses some solutions from EMA, in particular all those elementary pathways that are not at the boundary of the system but within it.

The metabolic model allows to identify all cellular pathways affected by each knockout mutation. Applying PERL scripts, we identified the number of mutants impairing the same pathway. Finally, all pathways relevant to intracellular survival were ranked with the highest redundancy at the top, and the key enzymes involved were listed (see Additional file 4 for results obtained from the knockout *in silico *experiment). A condensed view of these results is given in Figure 3.

Metabolic pathways severely affected by knockout mutations are the biosynthesis of valine/leucine/isoleucine, the purine, fucose, glycerol and lipid metabolism, lower glycolysis as well as serine and glutathione production and aspartate semialdehyde biosynthesis. It is important to note that on the other hand, a huge landscape of central metabolism is not important for intracellular growth. This demonstrates not only the robustness of these central metabolic pathways, but also the difference in the phenotypes revealed by the comparison of growth in full medium with the intracellular replication. The overview from EPA indicates that the glycerol metabolism is most critical for intracellular survival. Further genes important for intracellular replication of *L. monocytogenes *are *serC*, lmo0517, *glpD *and three genes involved in purine synthesis (*purS*, *purQ *and *purE*). The gene lmo0517 encodes a critical enzyme for glycolysis (periplasmic phosphoglycerate mutase), *glpD *encodes a glycerol-3-phosphate dehydrogenase which plays an important role in the lipid metabolism. In addition to *serC*, the purine operon, and lmo0517 (*pgm*), the more extensive EMA also suggests *aroB *to be critical for the intracellular survival. In addition to the result of EPA, *glpD *revealed to be less important for the system. This discrepancy might be explained by considering glycerol-3-phosphate as an intermediate metabolite for the glycolysis. On the other hand, lmo1031 putatively involved in fucose metabolism appears more important than *argD *for the cytoplasmic survival of *L. monocytogenes *as determined by EMA.

### IspE and the transporter Lmo0135-0137 are required *in vivo*

**Figure 4:**
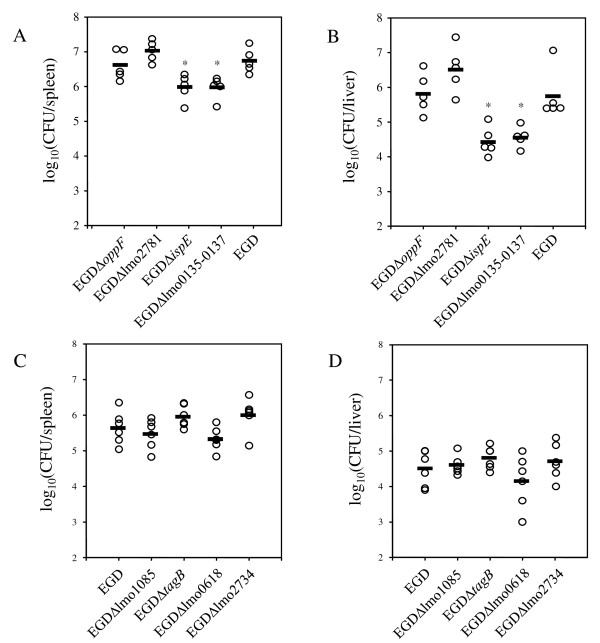
**Virulence of *L. monocytogenes *mutants *in vivo***. The bacterial loads of infected mice were determined three days and six days after intravenous infection with a sublethal dose of 2 × 10^3 ^CFU. The numbers of CFU per organ in spleen (A, C) and liver (B, D) of individual mice are shown. Bars indicate the mean values of the experimental groups. Statistical analysis of results was performed with the Tukey multiple comparison test (p < 0.05), and asterisks indicate significantly attenuated mutants. The experimental groups were ranked according to the bacterial load. The experiments were repeated twice with similar results.

*L. monocytogenes *mutants exhibiting intracellular growth deficiencies are often attenuated *in vivo*. Eight of the deletion mutants described above were therefore tested for their virulence properties in the BALB/c mouse infection model. For this purpose, groups of five 8- to 10-week-old female BALB/c mice were infected as described with a sub-lethal dose of the deletion mutants. Mice generally showed some signs of disease after three days of infection. At day three post infection with EGDΔlmo0135-0137 and EGDΔ*ispE*, the bacterial load of mice was significantly (*P *< 0.05) reduced in spleen and liver compared to mice infected with the wildtype-strain (Figure 4). The reduction was approximately one log_10 _in the spleen (Figure 4A) and more than 1.5 log_10 _in the liver (Figure 4B), respectively. We also determined the bacterial loads of mice infected with the strains EGDΔlmo0135-0137 and EGDΔ*ispE *six days after infection, and again observed an attenuation of these mutants compared to the wildtype strain (data not shown). Gene *ispE *(lmo0190) encodes a 4-diphosphocytidyl-2-C-methyl-D-erythritol-2-phosphate (CDP-ME) synthase involved in the alternative non-mevalonate (MEP) pathway of isoprenoid biosynthesis [21]. The operon lmo0135-0137 encodes a cysteine uptake-associated ABC-transporter [22]. It corresponds to a number of modes in the model (see Additional file 3 for input data of the model for calculation with flux balance analysis), thus supporting the importance of this route for the metabolic network.

The strains EGDΔlmo2734, EGDΔlmo1085 and EGDΔlmo618 also showed a minor reduction of bacterial counts, which was more pronounced in the spleen (Figure 4C) than in the liver (Figure 4D). This trend to reduced bacterial loads, however, was not statistically significant. In the case of the remaining strains EGDΔ*oppF*, EGDΔlmo2781 and EGDΔ*tagB*, no reduction of bacterial counts compared to mice infected with strain EGD was observed (Figure 4A, C), although *oppF *is induced during mouse infection [23].

## Discussion

Recently, two independent studies have disclosed the expression profile of *L. monocytogenes *during infection of Caco-2 cells and macrophages [11,12]. However, the up- or down-regulation of a gene under certain conditions does not always correlate with a phenotype of the respective mutant, possibly due to the high number of parameters changed, physiological side effects, catabolite repression, or transcriptional activities without impact. To overcome these limitations, we decided to complement our knowledge of listerial intracellular behaviour by the screening of a comprehensive mutant library, and report the results of a search for *L. monocytogenes *genes required during infection of and replication in human epithelial cells. This experimental genetic screen under defining conditions was finally combined with bioinformatic modelling.

### Surface proteins

Ami, Vip, and InlA are well known virulence factors that contribute to listerial adhesion to and invasion of eukaryotic cells, and there identification validates the screening procedure. Ami is an autolysin amidase involved in adhesion to epithelial cells [24], and InlA and Vip are required for entry into several non-phagocytic eukaryotic cells [25,26]. Besides InlA and Vip, four hypothetical cell surface proteins with LPXTG motif have been identified in this screening, namely Lmo1666, and the internalin-like proteins Lmo0514, Lmo2026, and Lmo0327 [3,27-30]. A lack of these four proteins affected the capability of strain EGD to invade Caco-2 cells only slightly, but a tissue-specific phenotype of these mutants as recently shown for Lmo2026 that plays a role during listerial multiplication in the brain cannot be excluded [31]. An lmo0441 mutant has been shown to grow wildtype-like in J774 macrophages and, in contrast to our data, also in Caco-2-derived C2Bbe1 cells [32].

### Virulence-related factors

Several of the genes that play a role intracellular replication as identified here are also known to be required for virulence of *L. monocytogenes *or other pathogens in mice. Upon application of signature-tagged transposon mutagenesis, a homologue of *agrA *encoding a response regulator involved in *Staphylococcus aureus *virulence [33], has recently been identified as virulence factor of *L. monocytogenes*, because its mutation resulted in a ten-fold increase in the 50% lethal dosis [34]. In contrast to this study, IDM inactivation of *agrA *resulted in a 3.4-fold attenuation of *L. monocytogenes *in Caco-2 cells, a discrepancy that might be explained by experimental conditions such as the MOI used. The inactivation of *sipX *(lmo1269) encoding a type I signal peptidase resulted in an eight-fold reduction of the intracellular multiplication of *L. monocytogenes *(data not shown) and also significantly affected the listerial virulence [35]. A deletion of *fur *responsible for oxidative stress response and iron storage led to a strong reduction of listerial virulence, but did not affect the growth of strain EGD-e in macrophages [36]. Our data clearly show the requirement of IspE for *L. monocytogenes *virulence, thus confirming a recent study that investigated mutants in other isoprenoid synthesis genes to demonstrate an *in vivo *role of the MEP pathway [37]. Listerial IspE exhibits a 62% similarity to IspE of *Bacillus cereus *and is highly conserved among *L. monocytogenes *strains. In contrast to our infection studies with Caco-2 cells (Table 1), isoprenoid biosynthesis is not required for listerial growth in macrophages [37]. Interestingly, IspE has been described as a novel protein target that elicits a strong antibody response from antiserum from rabbits infected with *L. monocytogenes*, suggesting that this protein is induced or upregulated during infection [38]. Three genes belonging to the category of membrane bioenergetics, namely lmo0091 and *atpA*/*atpB*, encode subunits of two ATP synthases for which a role in virulence has been demonstrated in *Francisella novicida *[39]. lmo2694, encoding a lysine decarboxylase, probably contributes to acid resistance by consuming intracellular protons [40].

### Transporters

A surprisingly high number of genes involved in various transport processes contribute to the intracellular replication of *L. monocytogenes*, justifying their consideration in the metabolism model (Figure 3). Obviously, the eukaryotic cytosol is exploited by the bacterium for osmoprotectants and nutrients such as sugars, phosphate and amino acids. This is in line with the assumption that within cells, *L. monocytogenes *utilizes sugars besides phosphorylated glucose, and intracellular peptides as a source of amino acids [7]. Mutant EGDΔlmo135-137 lacking the transporter Lmo0135-0137 associated with cysteine transport is growth attenuated in Caco-2 cells. This finding is in line with the assumption that the uptake of amino acids such as alanine, aspartate and glutamate from the host cell is a requirement for intracellular replicating of *L. monocytogenes *[41]. A similar observation has been made for OppABCDF essential for oligopeptide uptake. An *oppA *mutant showed retarded growth in macrophages and affects growth at low temperature [15]. lmo0135 (*ctpA*), but not OppABCDF, is essential for full virulence of *L. monocytogenes *in mice [4], suggesting a lack of available cysteine during systemic infection. In contrast to the oligopeptide-binding protein OppA of *L. monocytogenes *[15], Lmo0135-0137 does not mediate the transport of bialaphos, because strain EGDΔlmo0135-0137 showed a wildtype-like susceptibility of this toxic tripeptide (data not shown). Four other loci in the genome of *L. monocytogenes *are predicted to be involved in (oligo)peptide uptake, namely *dtpT*, lmo1265, lmo1712, and lmo0152 upregulated in macrophages [11], but the activity of only two of them has been disclosed [42-44].

The requirement of lmo2430 involved in ferrichrome transport, as well as of the iron uptake regulator Fur, points to a restriction of iron availability inside the cytosol, a finding that has also been reported for *Shigella flexneri *during intramacrophagic growth [45]. Within epithelial cells, *L. monocytogenes *competes for phosphate as shown by the identification of lmo2249 encoding a low-affinity inorganic phosphate transporter. The uptake of glycine betaine by *gbuA- *and *opuCA-*encoded transporters [46] contributes to osmotolerance of *L. monocytogenes *and thus to intracellular proliferation.

### Metabolism

Four genes involved in the synthesis of purines (*purA*, *purQ*, lmo1771) and pyrimidines (*pyrE*) are required for intracellular proliferation of *L. monocytogenes*, suggesting that these bases and nucleotides are not provided by the host cell, but must be synthesized by the bacterium. Two further genes identified here, *pdxK *and lmo2102, are involved in the biosynthesis of pyridoxine, a vitamin that contributes to transaminase activities during amino acid degradation. This observation also stresses that amino acid metabolism plays a key role for intracellular replication of *L. monocytogenes*, a finding that is supported by our network analysis of key flux modes for intracellular survival. A mutation in *nadB *demonstrate the requirement of nicotinate and nicotinamide metabolism during multiplication in Caco-2 cells, although a niacin-auxotrophic mutant of *L. monocytogenes *revealed no growth deficiencies following macrophage infection [7].

With respect to amino acid metabolism, the genes listed in Table 1, and the corresponding flux mode analysis (see Additional file 4 for results obtained from the knockout *in silico *experiment), show that besides the *de novo *biosynthesis of all aromatic and branched chain amino acids and arginine [7,12], an intact metabolism of methionine (lmo0594, lmo1495) and serine (lmo1235, *serC*) is required for multiplication of *L. monocytogenes *within Caco-2 cells.

The identification of lmo0517 and lmo2831 that encode a phosphoglycerate mutase and a phosphoglucomutase, respectively, support the finding that the pentose phosphate cycle rather than glycolysis is the predominant pathway of sugar metabolism of *L. monocytogenes *during proliferation in epithelial cells [12].

## Conclusion

An important outcome of the systems biology approach described here is the fact that *L. monocytogenes *overcomes several nutrient limitations within the epithelial cytosol by the expression of genes mainly involved in transport processes and in the metabolism of sugars, cofactors and nucleic acids. Although this pathogen is assumed to encounter a nutrient-rich surrounding after escape from the phagosome, it requires a surprisingly high number of metabolic pathways and factors during intracytosolic replication. Their identity became apparent by combining a genetic screen with flux mode calculations. As a result of this metabolic modelling, we could show that *Listeria *pathways for intracytoplasmatic survival are clearly distinct from the central set of genes essential for survival under optimal metabolic conditions, e.g. in full medium. Certain metabolic capabilities revealed to be important for intracellular survival, while the respective mutants did not show a phenotype in full medium. The results obtained are in line with the assumption that intracellular bacteria avoid the competition with the substrate requirements of their host cell, but prefer to use excess, storage, or garbage products of the cytosol [2]. Only few mutants identified in this screen were also attenuated in the mouse infection model, indicating that *L. monocytogenes *uses a huge number of partially redundant pathways and nutrient acquisition strategies, all of which contribute to its highly physiological flexibility within *in vivo *compartments.

## Methods

### Bacterial strains, growth conditions, cell lines, and mice

Strains used in this study are listed in Table 2. *Escherichia coli *strains XL2-blue (Stratagene, La Jolla, CA) and DH5α were cultivated in Luria-Bertani (LB) medium at 37°C. *L. monocytogenes *EGD (serovar 1/2a) was grown at 37°C in Brain Heart Infusion (BHI) or in modified minimal Welshimer's broth (mMWB) with 0.1 g histidine per liter [47]. The temperature applied was 30°C or 43°C in the presence of temperature-sensitive plasmid pLSV101. When necessary, media were supplemented with erythromycin (Serva, Electrophoresis GmbH, Heidelberg, Germany) to a final concentration of 300 μg/ml for *E. coli *or 5 μg/ml for *L. monocytogenes*. For solid media, 1.5% agar (w/v) was added. When appropriate, medium osmolarity was increased by the addition of 3% NaCl. Human colon epithelial cells (Caco-2 cells) were received from the American Type Culture Collection (ATCC HTB-37) and were cultured at 37°C with 5% CO_2 _in RPMI 1640 (Biochrom KG, Berlin, Germany) supplemented with 10% heat-inactivated fetal calf serum (FCS) (Perbio Science, Bonn, Germany). In order to monitor the growth of insertion mutants, overnight cultures were diluted 1:1000 in 250 μl BHI medium with erythromycin and shaken in a microtitre plate at 43°C for 24 h. The optical density of the cultures at 600 nm (OD_600_) was measured every 30 minutes in a colorimeter (Bioscreen C, Labsystem, France). For growth curves of deletion mutants, strains were grown overnight in the appropriate medium at 37°C, diluted as specified and shaken at 180 rpm in flasks until reaching stationary phase. The OD_600 _was measured each hour. Female BALB/c (H-2^d^) mice were purchased from Janvier (Le Geneste St. Isle, France), kept under conventional conditions, and used at 8-10 weeks of age.

**Table 2:** Strains and plasmids used in this study

name	characterization	reference
XL2-blue	*E. coli: recA1*, *endA1*, *gyrA96*, *thi-1*, *hsdR17*, *supE44*, *relA1*, *lac*, [F', *proA*B, *laclqZ*ΔM15Tn10(Tet^r^), Amy, Cam^r^]	Stratagene
DH5α	*E. coli: deoR*, *endA1, gyrA96*, *hsdR17*(r_k_-m_k+_),*recA1*, *relA1*, *supE44*, λlthi-1, Δ(*lacZYA*-*argFV169*)	[59]
EGD	*L. monocytogenes *Sv 1/2a, wildtype	S. H. E. Kaufmann
Pkp1	*L. monocytogenes *Sv 1/2a, Δ*plcA/hly/mpl/actA/plcB*, Kan^r^	[60]
EGDΔlmo0135-137	in-frame deletion mutant of a putative oligonucleotide transporter gene	This study
EGDΔ*ispE*	in-frame deletion mutant of lmo0190 involved in mevalonate biosynthesis	This study
EGDΔlmo0618	in-frame deletion mutant of a protein kinase gene	This study
EGDΔlmo1031-1036	In-frame deletion mutant of an operon responsible for glycerol metabolism	This study
EGDΔlmo1085	in-frame deletion mutant of lmo1085 involved in teichoic synthesis	This study
EGDΔ*tagB*	in-frame deletion mutant of *tagB *(lmo1088) involved in teichoic synthesis	This study
EGDΔlmo1506	in-frame deletion mutant of a putative transporter gene	This study
EGDΔ*oppF*	in-frame deletion mutant of the putative oligopeptide ABC transporter gene lmo2192	This study
EGDΔlmo2734	in-frame deletion mutant of lmo2734 encoding a putative sugar hydrolase	This study
EGDΔlmo2781	in-frame deletion mutant of lmo2781 involved in cellobiose metabolism	This study
pLSV101	Temperature-sensitive shuttle vector; Em^R^	[12]
EGDΔ*pdxK*	in-frame deletion mutant of *pdxK *(lmo0662) involved in biosynthesis of pyridoxine	This study

### General techniques

DNA manipulations and isolation of chromosomal DNA were performed according to standard protocols [48], and following the manufacturer's instructions. GeneRuler™ DNA Ladder Mix from MBI Fermentas (St. Leon-Rot, Germany) was used as a marker for DNA analysis. Plasmid DNA was transformed via electroporation by using a Bio-Rad Gene pulser II as recommended by the manufacturer. Polymerase chain reactions (PCRs) were carried out with Taq polymerase. Chromosomal DNA or an aliquot of a single colony resuspended in 100 μl H_2_O served as template for PCR. Listerial gene annotation was done according to the *Listeria *homepage of the Institut Pasteur http://genolist.pasteur.fr/ListiList/, and the oligonucleotides used for PCRs are listed in Additional file 5.

### Construction of a mutant library of *L. monocytogenes *EGD

A random mutant library of *L. monocytogenes *strain EGD Pkp1 was established as described recently [12]. Strain Pkp1 characterised by deletions of *plcA*, *hly*, *mpl*, *actA*, and *plcB *was chosen to avoid repeated identification of these well-known virulence genes. Briefly, chromosomal DNA was sonified, and the fractionated DNA was digested with MboI. Following separation by agarose gel electrophoresis, gel slices containing unirradiated DNA fragments from 200 to 400 bp were isolated. Purified chromosomal DNA was then ligated to MboI-restricted pLSV101. Sixteen independent ligation samples were transformed into *E. coli *XL2-blue, and a total of 3658 *E. coli *transformants was selected at 37°C on LB agar containing erythromycin. The average length of the cloned chromosomal fragments was 244 (+/-102) bp as determined by sequencing of 76 *E. coli *clones. Colonies were pooled in sets of 60-380 clones, and 300 ng DNA isolated from each pool was transformed into *L. monocytogenes *EGD. 3648 *L. monocytogenes *EGD fragment library clones were selected at 30°C in the presence of erythromycin, and single colonies were suspended in 200 μl BHI in a 96-well microtitre plate. 20 μl of each suspension were dropped with a multichannel pipette on BHI agar plates containing erythromycin and incubated at 43°C for two days [49]. Illegitimate insertion of pLSV101 without fragment was not observed. 1491 insertion mutants of *L. monocytogenes *EGD were isolated in 96-well microtitre plates in BHI and regrown over night at 43°C in the presence of erythromycin. Excision of the plasmid from the chromosomal site of insertion was obtained by repeated passage of a mutant clone on agar plates without erythromycin at 30°C, and the sites of plasmid insertion were determined by sequencing of plasmid-borne fragments derived from PCR with the primer pair LSV3 and LSV-4380rev [12]. Given a redundancy of 15% of the fragment library according to sequencing results, the 1491 clones tested here represent approximately 32% of the genome of strain *L. monocytogenes *EGD Pkp1 [12,50] and probably a higher percentage of functions encoded by operons. The stability of chromosomal pLSV101 integration during cell culture assays had already been demonstrated [12].

### Construction of deletion mutants

In-frame deletions of ten genes or gene loci were performed in the parental strains Sv1/2a EGD, namely of lmo1031-1036, lmo0135-0137, *ispE *(lmo190), lmo0618, *tagB *(lmo1088), lmo1085, lmo1506, lmo2192, lmo2734, and lmo2781. The standard procedure has recently been described [12,16] and is exemplified here for the deletion of lmo0190. Two fragments of approximately 500 bp were amplified from chromosomal DNA of strain EGD using the oligonucleotide pairs Lmo0189A/Lmo0189B and Lmo0191C/Lmo0191D, and then ligated via the introduced BglII sites. Following nested PCR using the oligonucleotides Lmo0189NestedAB and Lmo0191NestedCD and the ligation mixture as a template, the resulting fragment was cloned into pLSV101 via BamHI and EcoRI giving rise to pKS0190del. pKS0190del was then transformed into *L. monocytogenes *EGD by electroporation, and erythromycin-resistant bacteria growing at 43°C harbouring the chromosomally integrated plasmid were selected. Cointegrates were resolved by reiterated growth at 30°C, and erythromycin-sensitive clones were screened by PCR to identify the lmo0190 (*ispE*) deletion mutant. The gene deletions in all ten mutants listed in Table 2 were confirmed by sequencing. All primers are indicated in the Additional file 5.

### Caco-2 cell infection assays

Caco-2 cells (2.5 × 10^5 ^per well) were seeded in a 24-well culture plate and cultivated 22 h until infection. Cells were washed twice with Mg^2+^-and Ca^2+^-containing phosphate-buffered saline (PBS/Mg^2+^Ca^2+^) and covered for 1 h with 500 μl RPMI 1640 containing 2.0 μl of a bacterial culture grown over night. The average multiplicity of infection (MOI) was calculated to range from 6 to 14. To test deletion mutants, strains were grown in 20 ml BHI to late log-phase (OD_600_~1.0); aliquots were supplemented with glycerol at a final concentration of 15% and frozen at -80°C. Prior to infection, glycerol stocks were thawed, and the bacteria were sedimented and washed twice with PBS. After resuspension in 1 ml RPMI 1640, the number of viable bacteria was determined as CFU. The average MOI used here was 8 to 11.

After an infection period of 1 h, the Caco-2 cells were washed twice with PBS/Mg^2+^Ca^2+^. Extracellular bacteria were removed by adding 0.5 ml RPMI 1640 with 50 μg/ml gentamicin for 1 h, and the medium was then replaced by RPMI 1640 with 10 μg/ml gentamicin. At appropriate time points of incubation (2 h and 8 h), the infected cells were washed with PBS/Mg^2+^Ca^2+ ^and then lysed in 1 ml cold Triton X-100 (0.1%). Intracellular replication behaviour of the mutants and the wildtype was quantified by plating dilutions of the lysed cells on BHI agar plates that were incubated at 37°C and 43°C, respectively, for one day. If appropriate, the plates contained 5 μg/ml erythromycin. To examine adhesion properties of bacterial strains, the infection time was reduced to 35 min, and before lysis, cells were washed four times with PBS/Mg^2+^Ca^2+^. The capability of bacterial cells to invade Caco-2 cells was investigated as described above, but lysis of the epithelial cells was performed after 1 h, and a higher gentamicin concentration of 50 μg/ml was used. In all experiments, intact eukaryotic cell monolayers were observed prior to cell lysis.

### Mice infection assays

Female BALB/c (H-2d) mice were purchased (Janvier, Le Geneste St. Isle, France), kept under conventional conditions, and used at 8-10 weeks of age. Animal experiments were approved according to German federal law (Baden-Württemberg, permission number G-3/06). Mice were infected intravenously via the tail vein with a sublethal dose of *L. monocytogenes *serovar 1/2a EGD or *L. monocytogenes*-derived mutants in 0.2 ml endotoxin-free phosphate-buffered saline as indicated. Bacteria used for infection were in the logarithmic growth phase. The bacterial concentration of inoculated bacteria was calculated from OD_600 _and confirmed by plating on blood agar. Liver and spleen were removed three and six days after infection, respectively. The number of CFU per organ homogenate was determined by pour-plating dilutions of organ homogenates in BHI agar. The detection limit of the assay was 100 colony forming units (CFU) per organ. The statistical significance of the results of the mouse infection experiments was analyzed with the Tukey multiple comparison test [51] at the 0.05 significance level after logarithmic transformation of CFU and PFU values. This test analyzes the significance of the difference between all possible pairs of means with appropriate adjustment for the multiple testing. Calculations were performed using the WINKS statistical analysis software (TEXASOFT, Cedar Hill, USA).

### Modelling

The genome-scale metabolic network was reconstructed according to the latest annotation of *L. monocytogenes *EGD-e (GenBank accession number: NC_003210) based on the EGD-e genome sequence [13]. Reconstruction was done by systematic genome comparisons applying InGeno [52] and extensive sequence and domain analysis [53]. Metabolite terms and the topological structure were taken from the KEGG metabolic database. The topological structures were visualized and revised using the YANA and YANA square software [54,55]. A condensed network consisting of 167 metabolites, 155 enzymes and transporters was used to model the basic listerial metabolism based in a genome scale. Various carbon sources such as glucose, fucose, glycerol, acetate and citrate were considered. All essential amino acids are assumed to be synthesized *de novo *in the presence of sufficient amounts of ammonia. The null-space calculation from the convex basis resulted in 163 extreme pathways (EPs) and we calculated 20826 elementary modes (EMs) for the network.

## Abbreviations

MOI: multiplicity of infection; IDM: insertion duplication mutagenesis

## Authors' contributions

KS performed most experimental work with *Listeria *including infection assays and mutant construction, GG was responsible for the mice infection studies and CL for the modelling, WG and TD contributed to the conception and revised the manuscript, TMF designed and coordinated the study, and drafted the manuscript. All authors read and approved the final manuscript.

## Supplementary Material

Additional file 1 **Literature data regarding genes affecting intracellular replication of *L. monocytogenes***. Click here for file

Additional file 2 **Selected genes used for modelling of the listerial metabolism during intracellular replication**. Click here for file

Additional file 3 **Input data of the model for calculation with flux balance analysis (format: Suitable for Metatool or YANAsquare; Schwarz et al., 2007) including major transporters, amino acids and intermediary metabolites as shown in Figure **4. The model allows also further detailed analyses, e.g. of subnetworks as well as metabolic fluxes for identifying essential genes under different physiological growth conditions, such as medium or intracellular Caco-2 (PERL scripts were used for calculating different subsets). Click here for file

Additional file 4 **Results obtained from the knockout *in silico *experiment are summarized in this document (Tables S1-S3, Figure S1)**. Table S1: Metabolic flux modes calculated using extreme pathway analysis. Table S2: Key enzymes occurring in all the flux modes. Table S3: Metabolic flux modes critical for cytoplasmic survival interpreted as pathway equations. Figure S1: Elementary mode numbers affected by cytoplasmically attenuated mutants. Click here for file

Additional file 5 **Oligonucleotides used in this study**. Click here for file

Additional file 6 **Total cell number of mutants after invasion and after 7 hours of intracellular replication; the generation time is indicated**. Click here for file
