# Supplementary material for: Deciphering the intracellular metabolism of Listeria monocytogenes by mutant screening and modelling
Source: BMC Genomics. 2010 Oct 18;11:573. doi: 10.1186/1471-2164-11-573 (PMC3091722; doi:10.1186/1471-2164-11-573)
Supplement: Additional file 1 — Literature data regarding genes affecting intracellular replication of L. monocytogenes. [file 1471-2164-11-573-S1.DOC]

**Additional file 1**

**Literature data regarding genes affecting intracellular replication of *L. monocytogenes***

| *gene name* | lmo gene number | Intracellular replication (fold reduction) | functional category | literature | (putative) function/protein/enzyme |
| --- | --- | --- | --- | --- | --- |
|  |  |  |  |  |  |
| *uhpT* (*hpt*) | 0838 | 2.8-7 | transport/binding proteins and lipoproteins | [1] | hexose phosphate transporter |
| *lplA1* | 0931 | 8 | metabolism of lipids | [2] | lipoate protein ligase |
| *oppA* | 2196 | 8 | transport/binding proteins and lipoproteins | [3] | oligopeptide permease |
| *glpK2* | 1034 | 2-3 | metabolism of carbohydrates | [4] | glycerol kinase 2 |
| *thiD* | 0317 | 3.3 | metabolism of coenzymes and prosthetic groups | [5] | HMP kinase |
| *thiT* | 1429 | 2.2 | metabolism of coenzymes and prosthetic groups | [5] | thiamine transporter |
| *citG* | 2225 | 6 | metabolism of carbohydrates and related molecules | [6] | fumarate hydratase |
| *aroA* | 1600 | 10 | metabolism of amino acids and related molecules | [7] | 3-deoxy-7-phosphoheptulonate synthase |
| *aroB* | 1927 | 10 | metabolism of amino acids and related molecules | [7] | 3-dehydroquinate synthase |
| *aroE* | 1923 | 50 | metabolism of amino acids and related molecules | [7] | shikimate 5-dehydrogenase |
| *fhuC* | 1960 | 5 | transport/binding proteins and lipoproteins | [8] | ATP-binding protein of ferrichrome ABC transporter |
| *orfX* | 0206 | 8 | unknown | [9] |  |
| *ohrR* | 2200 | 4 | regulation | [9] | mediates organic hydroperoxide resistance |
| *prsA* | 2219 | 4 | protein secretion | [9] | posttranslocation molecular chaperone protein |
| *pycA* | 1072 | 100 | metabolism of carbohydrates and related molecules | [10] | pyruvate carboxylase |
| *inlA* | 0433 | 134 | cell surface proteins | [8] | internalin |
|  | 1971 | 4.1 | transport/binding proteins and lipoproteins | [8] | pentitol PTS system, enzyme IIC component |
|  | 1652 | 3.6 | transport/binding proteins and lipoproteins | [8] | ABC transporter, ATP binding protein |
|  | 2818 | 4.1 | transport/binding proteins and lipoproteins | [8] | transmembrane efflux protein |
| *eutB* | 1175 | 13.8 | metabolism of carbohydrates and related molecules | [8] | ethanolamine ammonia-lyase, heavy chain |
|  | 1538 | 4.8 | metabolism of carbohydrates and related molecules | [8] | glycerol kinase |
| *ilvD* | 1983 | 5.5 | metabolism of amino acids and related molecules | [8] | dihydroxy-acid dehydratase |
|  | 2434 | 2.3 | metabolism of amino acids and related molecules | [8] | glutamate decarboxylase |
|  | 2022 | 3 | metabolism of coenzymes and prosthetic groups | [8] | NifS-like protein required for NAD biosynthesis |
| *argD* | 1588 | 3.4 | metabolism of amino acids and related molecules | [8] | N-acetyl-ornithine aminotransferase |
|  | 0090 | 2.8 | membrane bioenergetics | [8] | ATP synthase a chain |
|  | 0705 | 5.4 | mobility and chemotaxis | [8] | flagellar hook-associated protein FlgK |
| *gidA* | 2810 | 4.3 | miscellaneous | [8] | tRNA-modifying enzyme |
|  | 1244 | 8.3 | metabolism of carbohydrates and related molecules | [8] | weakly similar to phosphoglycerate mutase 1 |
|  | 2734 | 4.1 | metabolism of carbohydrates and related molecules | [8] | weakly similar to sugar hydrolase |
|  | 0759 | 3.0 | unknown proteins | [8] | hypothetical glyoxalase family protein |

1. Chico-Calero I, Suarez M, Gonzalez-Zorn B, Scortti M, Slaghuis J, Goebel W, Vazquez-Boland JA: **Hpt, a bacterial homolog of the microsomal glucose- 6-phosphate translocase, mediates rapid intracellular proliferation in Listeria**. *Proc Natl Acad Sci U S A* 2002, **99**:431-436.

2. O'Riordan M, Moors MA, Portnoy DA: **Listeria intracellular growth and virulence require host-derived lipoic acid**. *Science* 2003, **302**:462-464.

3. Borezee E, Pellegrini E, Berche P: **OppA of Listeria monocytogenes, an oligopeptide-binding protein required for bacterial growth at low temperature and involved in intracellular survival**. *Infect Immun* 2000, **68**:7069-7077.

4. Joseph B, Mertins S, Stoll R, Schar J, Umesha KR, Luo Q, Muller-Altrock S, Goebel W: **Glycerol metabolism and PrfA activity in Listeria monocytogenes**. *J Bacteriol* 2008, **190**:5412-5430.

5. Schauer K, Stolz J, Scherer S, Fuchs TM: **Both thiamine uptake and biosynthesis of thiamine precursors are required for intracellular replication of Listeria monocytogenes** *J. Bacteriol.* 2009, **191**:2218-2227.

6. Gahan CG, Hill C: **The use of listeriolysin to identify in vivo induced genes in the gram-positive intracellular pathogen Listeria monocytogenes**. *Mol Microbiol* 2000, **36**:498-507.

7. Stritzker J, Janda J, Schoen C, Taupp M, Pilgrim S, Gentschev I, Schreier P, Geginat G, Goebel W: **Growth, virulence, and immunogenicity of Listeria monocytogenes aro mutants**. *Infect Immun* 2004, **72**:5622-5629.

8. Joseph B, Przybilla K, Stuhler C, Schauer K, Slaghuis J, Fuchs TM, Goebel W: **Identification of Listeria monocytogenes genes contributing to intracellular replication by expression profiling and mutant screening**. *J Bacteriol* 2006, **188**:556-568.

9. Chatterjee SS, Hossain H, Otten S, Kuenne C, Kuchmina K, Machata S, Domann E, Chakraborty T, Hain T: **Intracellular Gene Expression Profile of Listeria monocytogenes**. *Infect Immun* 2006, **74**:1323-1338.

10. Schär J, Stoll R, Schauer K, Loeffler DI, Eylert E, Joseph B, Eisenreich W, Fuchs TM, Goebel W: **Pyruvate Carboxylase Plays a Crucial Role in Carbon Metabolism of Extra- and Intracellularly Replicating Listeria monocytogenes**. *J Bacteriol* 2010, **192**:1774-1784.
