# Supplementary material for: Deciphering the intracellular metabolism of Listeria monocytogenes by mutant screening and modelling
Source: BMC Genomics. 2010 Oct 18;11:573. doi: 10.1186/1471-2164-11-573 (PMC3091722; doi:10.1186/1471-2164-11-573)
Supplement: Additional file 2 — Selected genes used for modelling of the listerial metabolism during intracellular replication. [file 1471-2164-11-573-S2.DOC]

**Additional file 2**

**Selected genes used for modelling of the listerial metabolism during intracellular replication**

| ***#*** | ***locus_tag*** | ***gene*** | ***EC_number*** | ***Process affected*** | ***Intracellular replication*** |
| --- | --- | --- | --- | --- | --- |
| 1 | ***lmo0517*** |  | 5.4.2.1 | PGM | 13.8 |
| 2 | ***lmo2825*** | *serC* | 2.6.1.52 | PSAT | 5.7 |
| 3 | ***lmo1771*** | *purS* | 6.3.5.3 | FGAMS | 11 |
| 4 | ***lmo1031*** |  | 5.3.1.25 | FucI | 11.1 |
| 5 | ***lmo1235*** |  | 2.7.2.4 | AspK | 5.9 |
| 6 | ***lmo1983*** | *ilvD* | 4.2.1.9 | ValEC, IleEC, LeuEC | 5.5 |
| 7 | ***lmo1984*** | *ilvB* | 2.2.1.6 | AcLacS, AcLacS2 | 5.5 |
| 8 | ***lmo1986*** | *ilvC* | 1.1.1.86 | ValEC, IleEC, LeuEC | 3.2 |
| 9 | ***lmo0594*** | *metX* | 2.3.1.31 | HSerTAc | 7.7 |
| 10 | ***lmo1927*** | *aroB* | 4.2.3.4 | DHQS | 2.8 |
| 11 | ***lmo2023*** | *nadB* | 1.4.3.16 | AspO | 3.4 |
| 12 | ***lmo1796*** | *purQ* | 6.3.5.3 | FGAMS | 12 |
| 13 | ***lmo1775*** | *purE* | 4.1.1.21 | AIRC | 5.5 |
| 14 | ***lmo0055*** | *purA* | 6.3.4.4 | ASuccS | 14.9 |
| 15 | ***lmo1538*** | *glpK* | 2.7.1.30 | GlycerolK | 4.8 |
| 16 | ***lmo1175*** | *eutB* | 4.3.1.7 | EthanolamineAmmoniaLya | 13.8 |
| 17 | ***lmo1293/*** | *glpD* | 1.1.5.3 | GlPDH_Q | 4.8 |
| 18 | ***lmo2134*** |  | 4.1.2.13 | FBPAld | 5.2 |
| 19 | ***lmo1588*** | *argD* | 2.6.1.11 | AcOrnTA | 3.4 |
| 20 | ***lmo2770*** |  | 6.3.2.2, 6.3.2.3 | GluCysLig | 5.8 |
