# Supplementary material for: Deciphering the intracellular metabolism of Listeria monocytogenes by mutant screening and modelling
Source: BMC Genomics. 2010 Oct 18;11:573. doi: 10.1186/1471-2164-11-573 (PMC3091722; doi:10.1186/1471-2164-11-573)
Supplement: Additional file 3 — Input data of the model for calculation with flux balance analysis (format: Suitable for Metatool or YANAsquare; Schwarz et al., 2007) including major transporters, amino acids and intermediary metabolites as shown in Figure 4. The model allows also further detailed analyses, e.g. of subnetworks as well as metabolic fluxes for identifying essential genes under different physiological growth conditions, such as medium or intracellular Caco-2 (PERL scripts were used for calculating different subsets). [file 1471-2164-11-573-S3.DOC]

**Additional file 3:** Input data of the model for calculation with flux balance analysis (format: Suitable for Metatool or YANAsquare; Schwarz et al., 2007) including major transporters, amino acids and intermediary metabolites as shown in Fig. 4. The model allows also further detailed analyses, e.g. of subnetworks as well as metabolic fluxes for identifying essential genes under different physiological growth conditions, such as medium or intracellular Caco-2 (PERL scripts were used for calculating different subsets).

| *#METATOOL input file* |
| --- |
| *#******************** |
| *#generated by libMETATOOLFileWriter Version 1.0* |
| *#on: 3/24/10 3:27 PM* |
|  |
| ENO PRAIso Nitrogen_Trans TPI LysEC GluS GluDH GlyHMTrans CitS TyrTA DHDPCS PyrDH_E2 DAHPS FBPAld DHQS PGI TrpS HomoSerDH EPSPS PGM PGK ICitDH PyrDH_E3 PTA ThrDHA PheTA OrnCarbTrans AcGluS AcGluR GlPDH_Q AcOrnTA R5PI GluSemiAld MDH AdenylatK TK2 TK1 DHQDA ThrS AspTA Acetate_Trans AspSemiAldDH ShiDH AlaDH GAPDH ACN TA 3PGDH ChorS PrephenateDH PSAT DHDPCR GlutAcetylTrans GlPDH SuccDH RPE GlyS FUM ArgSucc ProO |
|  |
| ASuccLya Gly_Trans PRT GluCysLig PyrK HomoCysTmet GlycerolK AnthranS Gln_Trans G6PDH GlnS Leu_Trans CarbK AsnS ShiK Glc_Trans TA_B_Val Fucose_Trans PSerP Leu_EC ArgSuccS Trp_Trans TA_B_Leu Tyr_Trans 6PGL Mae Ser_Trans SerDHA PyrCO AICARTF ArgDeiminase AldDH Succ_Trans FGAMS CarbPS Asn_Trans FucI Lys_Trans FucuPAld ADSL GlcPTS PyrDH_E1B PyrDH_E1A PFK Val_EC Met_Trans ACS AckA R5P_Trans Pro_Trans AIRS TA_B_Ile AcGluK Phe_Trans 6PGDH Glycerol_Trans AspK PPAT HomoSerK AspO ASuccS LDH IndoS SerTAc Thr_Trans SCAIRS FucuK IMPCyc Ile_Trans Ala_Trans GlcK PRPPS GARS DAPDC FBP HSerTAc ChorM Val_Trans AcLacS EthanolamineAmmoniaLya Glc6P PPDK AcLacS2 GARTF CysS Arg_Trans GluK Asp_Trans PrephenateDA Cit_Trans GlycerophosphodiesterPdisterase Cys_trans AIRC Ile_EC Glu_Trans |
|  |
| Arg AcGlu Ethanolamine FGAM PhePyr S7P EPSP Fuculose AIR DAHP AcCoA THDPC AcLac FGAR 6PGA Leu Cit CarbP CAIR 6PGL NH3 Succ Met Cys Fum E4P Shi Ru5P R5P Pro AcOrn MTHF G6P ThPP Tyr AspP DHAP Ile 2Ac2HB DHLipoamide AcP Lipoamide-E HomoSerP Thr DCEAMP AcGluAld 3PG IndoleGlycerolP PRA 3Methyl_AKPentanoate Asn Asp F6P Ala 2PG CPADeoxyRu5P PRPP Anth AKB Val ArgSucc AKG Ald AspSemiAld Fucose AMP HomoCys Trp Chor SCAIR Ser Citrulline 3PHPyr Mal CoA GAR GAP Shi3P Gln AcSer BPG meso_diaminopimelate 2HeThPP Glu X5P DHDPC Gly FAICAR Phe Glu5P AcGluP Glc PRAnth DHQ DHS FuculoseP PEP ICit PSer Ac OAA Orn HomoSer Prephenate HPYP FBP AICAR Lys 4MethyAKPentanoate AKiVal Glycerol S_AcDHLipoamide_E GluAld |
|  |
| NADPH CO2 THF FAD Leu_X Asp_X H2O2 NH3_X GDP Asn_X Gln_X Thr_X Ser_X Met_X FADH2 Tyr_X NADP+ Lac_X Glycerol_X Arg_X IMP FTHF ADP Gly_X Glc_biomass Phe_X Pyr Glu_X Trp_X HCO3 Glutathione Glc_X Acetate_X NAD+ LacAld Glycerophosphatidylethanolamine Succ_X Ile_X Ala_X H2S Quinol Cys_X NADH Val_X GTP ATP Lys_X Cit_X Fucose_X HS2O3 Glycerol3P Pro_X R5P_X Quinone |
|  |
| ENO : 2PG = PEP . |
| PRAIso : PRAnth = CPADeoxyRu5P . |
| Nitrogen_Trans : NH3_X = NH3 . |
| TPI : DHAP = GAP . |
| LysEC : AcCoA + THDPC = CoA + meso_diaminopimelate . |
| GluS : AKG + Gln + NADPH = 2 Glu + NADP+ . |
| GluDH : AKG + NADPH + NH3 = Glu + NADP+ . |
| GlyHMTrans : Gly + MTHF = Ser + THF . |
| CitS : AcCoA + OAA = Cit + CoA . |
| TyrTA : AKG + Tyr = Glu + HPYP . |
| DHDPCS : AspSemiAld + Pyr = DHDPC . |
| PyrDH_E2 : AcCoA + DHLipoamide = CoA + S_AcDHLipoamide_E . |
| DAHPS : E4P + PEP = DAHP . |
| FBPAld : FBP = DHAP + GAP . |
| DHQS : DAHP = DHQ . |
| PGI : G6P = F6P . |
| TrpS : IndoleGlycerolP + Ser = GAP + Trp . |
| HomoSerDH : HomoSer + NADP+ = AspSemiAld + NADPH . |
| EPSPS : PEP + Shi3P = EPSP . |
| PGM : 3PG = 2PG . |
| PGK : ADP + BPG = 3PG + ATP . |
| ICitDH : ICit + NADP+ = AKG + CO2 + NADPH . |
| PyrDH_E3 : DHLipoamide + NAD+ = Lipoamide-E + NADH . |
| PTA : AcCoA = AcP + CoA . |
| ThrDHA : Thr = AKB + NH3 . |
| PheTA : AKG + Phe = Glu + PhePyr . |
| OrnCarbTrans : CarbP + Orn = Citrulline . |
| AcGluS : AcCoA + Glu = AcGlu + CoA . |
| AcGluR : AcGluAld + NADP+ = AcGluP + NADPH . |
| GlPDH_Q : Glycerol3P + Quinone = DHAP + Quinol . |
| AcOrnTA : AcOrn + AKG = AcGluAld + Glu . |
| R5PI : Ru5P = R5P . |
| GluSemiAld : Glu5P + NADPH = GluAld + NADP+ . |
| MDH : Mal + NAD+ = NADH + OAA . |
| AdenylatK : AMP + ATP = ADP . |
| TK2 : E4P + X5P = F6P + GAP . |
| TK1 : R5P + X5P = GAP + S7P . |
| DHQDA : DHQ = DHS . |
| ThrS : HomoSerP = Thr . |
| AspTA : Glu + OAA = AKG + Asp . |
| Acetate_Trans : Ac = Acetate_X . |
| AspSemiAldDH : AspSemiAld + NADP+ = AspP + NADPH . |
| ShiDH : NADP+ + Shi = DHS + NADPH . |
| AlaDH : Ala + NAD+ = NADH + NH3 + Pyr . |
| GAPDH : GAP + NAD+ = BPG + NADH . |
| ACN : Cit = ICit . |
| TA : GAP + S7P = E4P + F6P . |
| 3PGDH : 3PG + NAD+ = 3PHPyr + NADH . |
| ChorS : EPSP = Chor . |
| PrephenateDH : NAD+ + Prephenate = CO2 + HPYP + NADH . |
| PSAT : 3PHPyr + Glu = AKG + PSer . |
| DHDPCR : NAD+ + THDPC = DHDPC + NADH . |
| GlutAcetylTrans : AcOrn + Glu = AcGlu + Orn . |
| GlPDH : Glycerol3P + NAD+ = DHAP + NADH . |
| SuccDH : FAD + Succ = FADH2 + Fum . |
| RPE : Ru5P = X5P . |
| GlyS : CO2 + MTHF + NADH + NH3 = Gly + NAD+ + THF . |
| FUM : Fum = Mal . |
| ArgSucc : ArgSucc = Arg + Fum . |
| ProO : GluAld + NADPH = NADP+ + Pro . |
| ASuccLya : DCEAMP = AMP + Fum . |
| Gly_Trans : Gly = Gly_X . |
| PRT : Anth + PRPP = PRAnth . |
| GluCysLig : 2 ATP + Cys + Glu + Gly = 2 ADP + Glutathione . |
| PyrK : ADP + PEP = ATP + Pyr . |
| HomoCysTmet : HomoCys = Met . |
| GlycerolK : ATP + Glycerol = ADP + Glycerol3P . |
| AnthranS : Chor + Gln = Anth + Glu + Pyr . |
| Gln_Trans : Gln = Gln_X . |
| G6PDH : G6P + NADP+ = 6PGL + NADPH . |
| GlnS : ATP + Glu + NH3 = ADP + Gln . |
| Leu_Trans : Leu = Leu_X . |
| CarbK : ATP + CO2 + NH3 = ADP + CarbP . |
| AsnS : Asp + ATP + Gln = AMP + Asn + Glu . |
| ShiK : ATP + Shi = ADP + Shi3P . |
| Glc_Trans : Glc = Glc_biomass . |
| TA_B_Val : AKiVal + Glu = AKG + Val . |
| Fucose_Trans : Fucose_X = Fucose . |
| PSerP : PSer = Ser . |
| Leu_EC : AcCoA + AKiVal + NAD+ = 4MethyAKPentanoate + CoA + NADH . |
| ArgSuccS : Asp + ATP + Citrulline = AMP + ArgSucc . |
| Trp_Trans : Trp = Trp_X . |
| TA_B_Leu : 4MethyAKPentanoate + Glu = AKG + Leu . |
| Tyr_Trans : Tyr = Tyr_X . |
| 6PGL : 6PGL = 6PGA . |
| Mae : Mal + NAD+ = CO2 + NADH + Pyr . |
| Ser_Trans : Ser = Ser_X . |
| SerDHA : Ser = NH3 + Pyr . |
| PyrCO : ATP + HCO3 + Pyr = ADP + OAA . |
| AICARTF : AICAR + FTHF = FAICAR + THF . |
| ArgDeiminase : Arg = Citrulline + NH3 . |
| AldDH : Ald + CoA + NAD+ = AcCoA + NADH . |
| Succ_Trans : Succ = Succ_X . |
| FGAMS : ATP + FGAR + Gln = ADP + FGAM + Glu . |
| CarbPS : 2 ATP + CO2 + Gln = 2 ADP + CarbP + Glu . |
| Asn_Trans : Asn = Asn_X . |
| FucI : Fucose = Fuculose . |
| Lys_Trans : Lys = Lys_X . |
| FucuPAld : FuculoseP = DHAP + LacAld . |
| ADSL : SCAIR = AICAR + Fum . |
| GlcPTS : Glc_X + PEP = G6P + Pyr . |
| PyrDH_E1B : 2HeThPP + Lipoamide-E = S_AcDHLipoamide_E + ThPP . |
| PyrDH_E1A : Pyr + ThPP = 2HeThPP + CO2 . |
| PFK : ATP + F6P = ADP + FBP . |
| Val_EC : AcLac + NADPH = AKiVal + NADP+ . |
| Met_Trans : Met = Met_X . |
| ACS : Ac + ATP + CoA = AcCoA + AMP . |
| AckA : AcP + ADP = Ac + ATP . |
| R5P_Trans : R5P = R5P_X . |
| Pro_Trans : Pro = Pro_X . |
| AIRS : ATP + FGAM = ADP + AIR . |
| TA_B_Ile : 3Methyl_AKPentanoate + Glu = AKG + Ile . |
| AcGluK : AcGlu + ATP = AcGluP + ADP . |
| Phe_Trans : Phe = Phe_X . |
| 6PGDH : 6PGA + NADP+ = CO2 + NADPH + Ru5P . |
| Glycerol_Trans : Glycerol_X = Glycerol . |
| AspK : Asp + ATP = ADP + AspP . |
| PPAT : Gln + PRPP = Glu + PRA . |
| HomoSerK : ATP + HomoSer = ADP + HomoSerP . |
| AspO : Asp = H2O2 + NH3 + OAA . |
| ASuccS : Asp + GTP + IMP = DCEAMP + GDP . |
| LDH : NADH + Pyr = Lac_X + NAD+ . |
| IndoS : CPADeoxyRu5P = CO2 + IndoleGlycerolP . |
| SerTAc : AcCoA + Ser = AcSer + CoA . |
| Thr_Trans : Thr = Thr_X . |
| SCAIRS : CAIR = SCAIR . |
| FucuK : ATP + Fuculose = ADP + FuculoseP . |
| IMPCyc : FAICAR = IMP . |
| Ile_Trans : Ile = Ile_X . |
| Ala_Trans : Ala = Ala_X . |
| GlcK : ATP + Glc = ADP + G6P . |
| PRPPS : ATP + R5P = AMP + PRPP . |
| GARS : ATP + Gly + PRA = ADP + GAR . |
| DAPDC : meso_diaminopimelate = CO2 + Lys . |
| FBP : FBP = F6P . |
| HSerTAc : AcCoA + HomoSer + HS2O3 = Ac + CoA + HomoCys . |
| ChorM : Chor = Prephenate . |
| Val_Trans : Val = Val_X . |
| AcLacS : 2 Pyr = AcLac + CO2 . |
| EthanolamineAmmoniaLya : Ethanolamine = Ald + NH3 . |
| Glc6P : G6P = Glc . |
| PPDK : ATP + Pyr = AMP + PEP . |
| AcLacS2 : AKB + Pyr = 2Ac2HB + CO2 . |
| GARTF : FTHF + GAR = FGAR + THF . |
| CysS : AcSer + H2S = Ac + Cys . |
| Arg_Trans : Arg = Arg_X . |
| GluK : ATP + Glu = ADP + Glu5P . |
| Asp_Trans : Asp = Asp_X . |
| PrephenateDA : Prephenate = CO2 + PhePyr . |
| Cit_Trans : Cit_X = Cit . |
| GlycerophosphodiesterPdisterase : Glycerophosphatidylethanolamine = Ethanolamine + Glycerol3P . |
| Cys_trans : Cys = Cys_X . |
| AIRC : AIR + CO2 = CAIR . |
| Ile_EC : 2Ac2HB + NADPH = 3Methyl_AKPentanoate + NADP+ . |
| Glu_Trans : Glu = Glu_X . |
