# Supplementary material for: Deciphering the intracellular metabolism of Listeria monocytogenes by mutant screening and modelling
Source: BMC Genomics. 2010 Oct 18;11:573. doi: 10.1186/1471-2164-11-573 (PMC3091722; doi:10.1186/1471-2164-11-573)

**Additional file 4:** Results obtained from the knockout *in silico* experiment are summarized in this document.

**Table S1: Metabolic flux modes calculated using extreme pathway analysis.**

Modes using Extreme Pathway Analysis (EPA) are enumerated.

| **#,"Activity","Flux sum","Reversible","Pathlength","Reactions"** |
| --- |
| 1,"1.0","2","true","2","(1 GlPDH) (-1 GlPDH_Q)" |
| 2,"1.0","6","false","6","(1 ACN) (1 Cit_Trans) (1 Glu_Trans) (1 GluDH) (1 ICitDH) (1 Nitrogen_Trans)" |
| 3,"1.0","8","false","8","(1 AdenylatK) (-1 ENO) (-1 GAPDH) (-1 GlPDH_Q) (-1 PGK) (-1 PGM) (1 PPDK) (-1 TPI)" |
| 4,"1.0","2","false","2","(1 Glc6P) (1 GlcK)" |
| 5,"1.0","3","false","3","(1 Ala_Trans) (-1 AlaDH) (1 Nitrogen_Trans)" |
| 6,"1.0","1","false","1","(1 LDH)" |
| 7,"1.0","3","false","3","(1 AspO) (1 AspTA) (1 GluDH)" |
| 8,"1.0","9","false","9","(1 ACN) (1 Cit_Trans) (1 GluDH) (1 GluK) (1 GluSemiAld) (1 ICitDH) (1 Nitrogen_Trans) (1 Pro_Trans) (1 ProO)" |
| 9,"1.0","22","false","10","(2 FBP) (-2 FBPAld) (5 GlPDH_Q) (3 R5P_Trans) (2 R5PI) (-2 RPE) (-1 TA) (-1 TK1) (-1 TK2) (3 TPI)" |
| 10,"1.0","9","false","9","(1 Acetate_Trans) (1 AckA) (1 Asp_Trans) (1 AspTA) (1 Cit_Trans) (-1 CitS) (1 GluDH) (1 Nitrogen_Trans) (1 PTA)" |
| 11,"1.0","14","false","14","(1 Acetate_Trans) (1 AckA) (1 AspK) (-1 AspSemiAldDH) (1 AspTA) (1 Cit_Trans) (-1 CitS) (1 GluDH) (-1 HomoSerDH) (1 HomoSerK) (1 Nitrogen_Trans) (1 PTA) (1 Thr_Trans) (1 ThrS)" |
| 12,"1.0","19","false","18","(1 Acetate_Trans) (1 AckA) (1 AcLacS2) (1 AspK) (-1 AspSemiAldDH) (1 AspTA) (1 Cit_Trans) (-1 CitS) (2 GluDH) (-1 HomoSerDH) (1 HomoSerK) (1 Ile_EC) (1 Ile_Trans) (1 Nitrogen_Trans) (1 PTA) (1 TA_B_Ile) (1 ThrDHA) (1 ThrS)" |
| 13,"1.0","4","false","4","(1 AckA) (1 ACS) (1 AdenylatK) (1 PTA)" |
| 14,"1.0","2","false","2","(1 FBP) (1 PFK)" |
| 15,"1.0","7","false","7","(1 Acetate_Trans) (1 AckA) (1 PTA) (1 PyrDH_E1A) (1 PyrDH_E1B) (-1 PyrDH_E2) (1 PyrDH_E3)" |
| 16,"1.0","28","false","14","(2 ENO) (2 GAPDH) (2 GlcPTS) (3 GlPDH_Q) (2 PGI) (2 PGK) (2 PGM) (3 R5P_Trans) (2 R5PI) (-2 RPE) (-1 TA) (-1 TK1) (-1 TK2) (3 TPI)" |
| 17,"1.0","9","false","9","(1 ENO) (1 FBPAld) (1 GAPDH) (1 GlcPTS) (-1 GlPDH_Q) (1 PFK) (1 PGI) (1 PGK) (1 PGM)" |
| 18,"1.0","12","false","12","(1 AspK) (-1 AspSemiAldDH) (1 AspTA) (1 Cit_Trans) (-1 CitS) (1 DAPDC) (-1 DHDPCR) (1 DHDPCS) (1 GluDH) (1 Lys_Trans) (1 LysEC) (1 Nitrogen_Trans)" |
| 19,"1.0","9","false","9","(1 Acetate_Trans) (1 AckA) (1 Cit_Trans) (-1 CitS) (-1 FUM) (-1 MDH) (1 PTA) (1 Succ_Trans) (-1 SuccDH)" |
| 20,"1.0","7","false","7","(1 Acetate_Trans) (1 AckA) (1 AldDH) (1 EthanolamineAmmoniaLya) (1 GlycerophosphodiesterPdisterase) (-1 Nitrogen_Trans) (1 PTA)" |
| 21,"1.0","5","false","5","(1 Asp_Trans) (1 AspTA) (1 GluDH) (1 Nitrogen_Trans) (1 PyrCO)" |
| 22,"1.0","10","false","10","(1 AspK) (-1 AspSemiAldDH) (1 AspTA) (1 GluDH) (-1 HomoSerDH) (1 HomoSerK) (1 Nitrogen_Trans) (1 PyrCO) (1 Thr_Trans) (1 ThrS)" |
| 23,"1.0","15","false","14","(1 AcLacS2) (1 AspK) (-1 AspSemiAldDH) (1 AspTA) (2 GluDH) (-1 HomoSerDH) (1 HomoSerK) (1 Ile_EC) (1 Ile_Trans) (1 Nitrogen_Trans) (1 PyrCO) (1 TA_B_Ile) (1 ThrDHA) (1 ThrS)" |
| 24,"1.0","10","false","10","(-1 Acetate_Trans) (1 ACN) (1 ACS) (1 AdenylatK) (1 CitS) (1 Glu_Trans) (1 GluDH) (1 ICitDH) (1 Nitrogen_Trans) (1 PyrCO)" |
| 25,"1.0","13","false","13","(-1 Acetate_Trans) (1 ACN) (1 ACS) (1 AdenylatK) (1 CitS) (1 GluDH) (1 GluK) (1 GluSemiAld) (1 ICitDH) (1 Nitrogen_Trans) (1 Pro_Trans) (1 ProO) (1 PyrCO)" |
| 26,"1.0","11","false","11","(1 ACN) (1 CitS) (1 Glu_Trans) (1 GluDH) (1 ICitDH) (1 Nitrogen_Trans) (1 PyrCO) (1 PyrDH_E1A) (1 PyrDH_E1B) (-1 PyrDH_E2) (1 PyrDH_E3)" |
| 27,"1.0","14","false","14","(1 ACN) (1 CitS) (1 GluDH) (1 GluK) (1 GluSemiAld) (1 ICitDH) (1 Nitrogen_Trans) (1 Pro_Trans) (1 ProO) (1 PyrCO) (1 PyrDH_E1A) (1 PyrDH_E1B) (-1 PyrDH_E2) (1 PyrDH_E3)" |
| 28,"1.0","14","false","14","(-1 Acetate_Trans) (1 ACS) (1 AdenylatK) (1 AspK) (-1 AspSemiAldDH) (1 AspTA) (1 DAPDC) (-1 DHDPCR) (1 DHDPCS) (1 GluDH) (1 Lys_Trans) (1 LysEC) (1 Nitrogen_Trans) (1 PyrCO)" |
| 29,"1.0","15","false","15","(1 AspK) (-1 AspSemiAldDH) (1 AspTA) (1 DAPDC) (-1 DHDPCR) (1 DHDPCS) (1 GluDH) (1 Lys_Trans) (1 LysEC) (1 Nitrogen_Trans) (1 PyrCO) (1 PyrDH_E1A) (1 PyrDH_E1B) (-1 PyrDH_E2) (1 PyrDH_E3)" |
| 30,"1.0","5","false","5","(-1 FUM) (-1 MDH) (1 PyrCO) (1 Succ_Trans) (-1 SuccDH)" |
| 31,"1.0","9","false","9","(1 ACN) (1 AldDH) (1 CitS) (1 EthanolamineAmmoniaLya) (1 Glu_Trans) (1 GluDH) (1 GlycerophosphodiesterPdisterase) (1 ICitDH) (1 PyrCO)" |
| 32,"1.0","12","false","12","(1 ACN) (1 AldDH) (1 CitS) (1 EthanolamineAmmoniaLya) (1 GluDH) (1 GluK) (1 GluSemiAld) (1 GlycerophosphodiesterPdisterase) (1 ICitDH) (1 Pro_Trans) (1 ProO) (1 PyrCO)" |
| 33,"1.0","13","false","13","(1 AldDH) (1 AspK) (-1 AspSemiAldDH) (1 AspTA) (1 DAPDC) (-1 DHDPCR) (1 DHDPCS) (1 EthanolamineAmmoniaLya) (1 GluDH) (1 GlycerophosphodiesterPdisterase) (1 Lys_Trans) (1 LysEC) (1 PyrCO)" |
| 34,"1.0","7","false","7","(1 Acetate_Trans) (1 AckA) (1 Cit_Trans) (-1 CitS) (1 Mae) (-1 MDH) (1 PTA)" |
| 35,"1.0","3","false","3","(1 Mae) (-1 MDH) (1 PyrCO)" |
| 36,"1.0","25","false","19","(1 AcGluK) (-1 AcGluR) (1 ACN) (-1 AcOrnTA) (1 AdenylatK) (1 Arg_Trans) (1 ArgSucc) (1 ArgSuccS) (1 AspTA) (1 CarbPS) (1 Cit_Trans) (1 FUM) (4 GluDH) (-1 GluS) (1 GlutAcetylTrans) (1 ICitDH) (1 MDH) (4 Nitrogen_Trans) (1 OrnCarbTrans)" |
| 37,"1.0","8","false","8","(1 AdenylatK) (1 ArgDeiminase) (1 ArgSucc) (1 ArgSuccS) (1 AspTA) (1 FUM) (1 GluDH) (1 MDH)" |
| 38,"1.0","29","false","22","(-1 Acetate_Trans) (1 AcGluK) (-1 AcGluR) (1 ACN) (-1 AcOrnTA) (1 ACS) (2 AdenylatK) (1 Arg_Trans) (1 ArgSucc) (1 ArgSuccS) (1 AspTA) (1 CarbPS) (1 CitS) (1 FUM) (4 GluDH) (-1 GluS) (1 GlutAcetylTrans) (1 ICitDH) (1 MDH) (4 Nitrogen_Trans) (1 OrnCarbTrans) (1 PyrCO)" |
| 39,"1.0","30","false","24","(1 AcGluK) (-1 AcGluR) (1 ACN) (-1 AcOrnTA) (1 AdenylatK) (1 Arg_Trans) (1 ArgSucc) (1 ArgSuccS) (1 AspTA) (1 CarbPS) (1 CitS) (1 FUM) (4 GluDH) (-1 GluS) (1 GlutAcetylTrans) (1 ICitDH) (1 MDH) (4 Nitrogen_Trans) (1 OrnCarbTrans) (1 PyrCO) (1 PyrDH_E1A) (1 PyrDH_E1B) (-1 PyrDH_E2) (1 PyrDH_E3)" |
| 40,"1.0","28","false","23","(1 AcGluK) (-1 AcGluR) (1 ACN) (-1 AcOrnTA) (1 AdenylatK) (1 AldDH) (1 Arg_Trans) (1 ArgSucc) (1 ArgSuccS) (1 AspTA) (1 CarbPS) (1 CitS) (1 EthanolamineAmmoniaLya) (1 FUM) (4 GluDH) (-1 GluS) (1 GlutAcetylTrans) (1 GlycerophosphodiesterPdisterase) (1 ICitDH) (1 MDH) (3 Nitrogen_Trans) (1 OrnCarbTrans) (1 PyrCO)" |
| 41,"1.0","19","false","17","(1 3PGDH) (1 Acetate_Trans) (1 Asp_Trans) (1 AspTA) (1 Cit_Trans) (-1 CitS) (1 Cys_trans) (1 CysS) (1 GAPDH) (1 GlPDH_Q) (2 GluDH) (2 Nitrogen_Trans) (1 PGK) (1 PSAT) (1 PSerP) (1 SerTAc) (1 TPI)" |
| 42,"1.0","24","false","22","(1 3PGDH) (1 Acetate_Trans) (1 AspK) (-1 AspSemiAldDH) (1 AspTA) (1 Cit_Trans) (-1 CitS) (1 Cys_trans) (1 CysS) (1 GAPDH) (1 GlPDH_Q) (2 GluDH) (-1 HomoSerDH) (1 HomoSerK) (2 Nitrogen_Trans) (1 PGK) (1 PSAT) (1 PSerP) (1 SerTAc) (1 Thr_Trans) (1 ThrS) (1 TPI)" |
| 43,"1.0","29","false","26","(1 3PGDH) (1 Acetate_Trans) (1 AcLacS2) (1 AspK) (-1 AspSemiAldDH) (1 AspTA) (1 Cit_Trans) (-1 CitS) (1 Cys_trans) (1 CysS) (1 GAPDH) (1 GlPDH_Q) (3 GluDH) (-1 HomoSerDH) (1 HomoSerK) (1 Ile_EC) (1 Ile_Trans) (2 Nitrogen_Trans) (1 PGK) (1 PSAT) (1 PSerP) (1 SerTAc) (1 TA_B_Ile) (1 ThrDHA) (1 ThrS) (1 TPI)" |
| 44,"1.0","14","false","14","(1 3PGDH) (1 ACS) (1 AdenylatK) (1 Cys_trans) (1 CysS) (1 GAPDH) (1 GlPDH_Q) (1 GluDH) (1 Nitrogen_Trans) (1 PGK) (1 PSAT) (1 PSerP) (1 SerTAc) (1 TPI)" |
| 45,"1.0","17","false","17","(1 3PGDH) (1 Acetate_Trans) (1 Cys_trans) (1 CysS) (1 GAPDH) (1 GlPDH_Q) (1 GluDH) (1 Nitrogen_Trans) (1 PGK) (1 PSAT) (1 PSerP) (1 PyrDH_E1A) (1 PyrDH_E1B) (-1 PyrDH_E2) (1 PyrDH_E3) (1 SerTAc) (1 TPI)" |
| 46,"1.0","19","false","19","(1 3PGDH) (1 Acetate_Trans) (1 Cit_Trans) (-1 CitS) (1 Cys_trans) (1 CysS) (-1 FUM) (1 GAPDH) (1 GlPDH_Q) (1 GluDH) (-1 MDH) (1 Nitrogen_Trans) (1 PGK) (1 PSAT) (1 PSerP) (1 SerTAc) (1 Succ_Trans) (-1 SuccDH) (1 TPI)" |
| 47,"1.0","15","false","15","(1 3PGDH) (1 Acetate_Trans) (1 AldDH) (1 Cys_trans) (1 CysS) (1 EthanolamineAmmoniaLya) (1 GAPDH) (1 GlPDH_Q) (1 GluDH) (1 GlycerophosphodiesterPdisterase) (1 PGK) (1 PSAT) (1 PSerP) (1 SerTAc) (1 TPI)" |
| 48,"1.0","218","false","35","(3 3PGDH) (6 AdenylatK) (6 ADSL) (6 AICARTF) (6 AIRC) (6 AIRS) (6 Asp_Trans) (6 AspTA) (4 FBP) (-4 FBPAld) (6 FGAMS) (6 FUM) (3 GAPDH) (6 GARS) (6 GARTF) (13 GlPDH_Q) (21 GluDH) (-12 GluS) (-3 GlyHMTrans) (3 GlyS) (6 IMPCyc) (6 MDH) (24 Nitrogen_Trans) (3 PGK) (6 PPAT) (6 PRPPS) (3 PSAT) (3 PSerP) (4 R5PI) (-4 RPE) (6 SCAIRS) (-2 TA) (-2 TK1) (-2 TK2) (9 TPI)" |
| 49,"1.0","248","false","40","(3 3PGDH) (6 AdenylatK) (6 ADSL) (6 AICARTF) (6 AIRC) (6 AIRS) (6 AspK) (-6 AspSemiAldDH) (6 AspTA) (4 FBP) (-4 FBPAld) (6 FGAMS) (6 FUM) (3 GAPDH) (6 GARS) (6 GARTF) (13 GlPDH_Q) (21 GluDH) (-12 GluS) (-3 GlyHMTrans) (3 GlyS) (-6 HomoSerDH) (6 HomoSerK) (6 IMPCyc) (6 MDH) (24 Nitrogen_Trans) (3 PGK) (6 PPAT) (6 PRPPS) (3 PSAT) (3 PSerP) (4 R5PI) (-4 RPE) (6 SCAIRS) (-2 TA) (6 Thr_Trans) (6 ThrS) (-2 TK1) (-2 TK2) (9 TPI)" |
| 50,"1.0","278","false","44","(3 3PGDH) (6 AcLacS2) (6 AdenylatK) (6 ADSL) (6 AICARTF) (6 AIRC) (6 AIRS) (6 AspK) (-6 AspSemiAldDH) (6 AspTA) (4 FBP) (-4 FBPAld) (6 FGAMS) (6 FUM) (3 GAPDH) (6 GARS) (6 GARTF) (13 GlPDH_Q) (27 GluDH) (-12 GluS) (-3 GlyHMTrans) (3 GlyS) (-6 HomoSerDH) (6 HomoSerK) (6 Ile_EC) (6 Ile_Trans) (6 IMPCyc) (6 MDH) (24 Nitrogen_Trans) (3 PGK) (6 PPAT) (6 PRPPS) (3 PSAT) (3 PSerP) (4 R5PI) (-4 RPE) (6 SCAIRS) (-2 TA) (6 TA_B_Ile) (6 ThrDHA) (6 ThrS) (-2 TK1) (-2 TK2) (9 TPI)" |
| 51,"1.0","248","false","39","(3 3PGDH) (-6 Acetate_Trans) (6 ACN) (6 ACS) (12 AdenylatK) (6 ADSL) (6 AICARTF) (6 AIRC) (6 AIRS) (6 CitS) (4 FBP) (-4 FBPAld) (6 FGAMS) (6 FUM) (3 GAPDH) (6 GARS) (6 GARTF) (13 GlPDH_Q) (6 Glu_Trans) (21 GluDH) (-12 GluS) (-3 GlyHMTrans) (3 GlyS) (6 ICitDH) (6 IMPCyc) (6 MDH) (24 Nitrogen_Trans) (3 PGK) (6 PPAT) (6 PRPPS) (3 PSAT) (3 PSerP) (4 R5PI) (-4 RPE) (6 SCAIRS) (-2 TA) (-2 TK1) (-2 TK2) (9 TPI)" |
| 52,"1.0","266","false","42","(3 3PGDH) (-6 Acetate_Trans) (6 ACN) (6 ACS) (12 AdenylatK) (6 ADSL) (6 AICARTF) (6 AIRC) (6 AIRS) (6 CitS) (4 FBP) (-4 FBPAld) (6 FGAMS) (6 FUM) (3 GAPDH) (6 GARS) (6 GARTF) (13 GlPDH_Q) (21 GluDH) (6 GluK) (-12 GluS) (6 GluSemiAld) (-3 GlyHMTrans) (3 GlyS) (6 ICitDH) (6 IMPCyc) (6 MDH) (24 Nitrogen_Trans) (3 PGK) (6 PPAT) (6 Pro_Trans) (6 ProO) (6 PRPPS) (3 PSAT) (3 PSerP) (4 R5PI) (-4 RPE) (6 SCAIRS) (-2 TA) (-2 TK1) (-2 TK2) (9 TPI)" |
| 53,"1.0","254","false","41","(3 3PGDH) (6 ACN) (6 AdenylatK) (6 ADSL) (6 AICARTF) (6 AIRC) (6 AIRS) (6 CitS) (4 FBP) (-4 FBPAld) (6 FGAMS) (6 FUM) (3 GAPDH) (6 GARS) (6 GARTF) (13 GlPDH_Q) (6 Glu_Trans) (21 GluDH) (-12 GluS) (-3 GlyHMTrans) (3 GlyS) (6 ICitDH) (6 IMPCyc) (6 MDH) (24 Nitrogen_Trans) (3 PGK) (6 PPAT) (6 PRPPS) (3 PSAT) (3 PSerP) (6 PyrDH_E1A) (6 PyrDH_E1B) (-6 PyrDH_E2) (6 PyrDH_E3) (4 R5PI) (-4 RPE) (6 SCAIRS) (-2 TA) (-2 TK1) (-2 TK2) (9 TPI)" |
| 54,"1.0","272","false","44","(3 3PGDH) (6 ACN) (6 AdenylatK) (6 ADSL) (6 AICARTF) (6 AIRC) (6 AIRS) (6 CitS) (4 FBP) (-4 FBPAld) (6 FGAMS) (6 FUM) (3 GAPDH) (6 GARS) (6 GARTF) (13 GlPDH_Q) (21 GluDH) (6 GluK) (-12 GluS) (6 GluSemiAld) (-3 GlyHMTrans) (3 GlyS) (6 ICitDH) (6 IMPCyc) (6 MDH) (24 Nitrogen_Trans) (3 PGK) (6 PPAT) (6 Pro_Trans) (6 ProO) (6 PRPPS) (3 PSAT) (3 PSerP) (6 PyrDH_E1A) (6 PyrDH_E1B) (-6 PyrDH_E2) (6 PyrDH_E3) (4 R5PI) (-4 RPE) (6 SCAIRS) (-2 TA) (-2 TK1) (-2 TK2) (9 TPI)" |
| 55,"1.0","230","false","37","(3 3PGDH) (6 AdenylatK) (6 ADSL) (6 AICARTF) (6 AIRC) (6 AIRS) (6 Asp_Trans) (6 AspTA) (4 ENO) (6 FGAMS) (6 FUM) (7 GAPDH) (6 GARS) (6 GARTF) (4 GlcPTS) (9 GlPDH_Q) (21 GluDH) (-12 GluS) (-3 GlyHMTrans) (3 GlyS) (6 IMPCyc) (6 MDH) (24 Nitrogen_Trans) (4 PGI) (7 PGK) (4 PGM) (6 PPAT) (6 PRPPS) (3 PSAT) (3 PSerP) (4 R5PI) (-4 RPE) (6 SCAIRS) (-2 TA) (-2 TK1) (-2 TK2) (9 TPI)" |
| 56,"1.0","260","false","42","(3 3PGDH) (6 AdenylatK) (6 ADSL) (6 AICARTF) (6 AIRC) (6 AIRS) (6 AspK) (-6 AspSemiAldDH) (6 AspTA) (4 ENO) (6 FGAMS) (6 FUM) (7 GAPDH) (6 GARS) (6 GARTF) (4 GlcPTS) (9 GlPDH_Q) (21 GluDH) (-12 GluS) (-3 GlyHMTrans) (3 GlyS) (-6 HomoSerDH) (6 HomoSerK) (6 IMPCyc) (6 MDH) (24 Nitrogen_Trans) (4 PGI) (7 PGK) (4 PGM) (6 PPAT) (6 PRPPS) (3 PSAT) (3 PSerP) (4 R5PI) (-4 RPE) (6 SCAIRS) (-2 TA) (6 Thr_Trans) (6 ThrS) (-2 TK1) (-2 TK2) (9 TPI)" |
| 57,"1.0","290","false","46","(3 3PGDH) (6 AcLacS2) (6 AdenylatK) (6 ADSL) (6 AICARTF) (6 AIRC) (6 AIRS) (6 AspK) (-6 AspSemiAldDH) (6 AspTA) (4 ENO) (6 FGAMS) (6 FUM) (7 GAPDH) (6 GARS) (6 GARTF) (4 GlcPTS) (9 GlPDH_Q) (27 GluDH) (-12 GluS) (-3 GlyHMTrans) (3 GlyS) (-6 HomoSerDH) (6 HomoSerK) (6 Ile_EC) (6 Ile_Trans) (6 IMPCyc) (6 MDH) (24 Nitrogen_Trans) (4 PGI) (7 PGK) (4 PGM) (6 PPAT) (6 PRPPS) (3 PSAT) (3 PSerP) (4 R5PI) (-4 RPE) (6 SCAIRS) (-2 TA) (6 TA_B_Ile) (6 ThrDHA) (6 ThrS) (-2 TK1) (-2 TK2) (9 TPI)" |
| 58,"1.0","260","false","41","(3 3PGDH) (-6 Acetate_Trans) (6 ACN) (6 ACS) (12 AdenylatK) (6 ADSL) (6 AICARTF) (6 AIRC) (6 AIRS) (6 CitS) (4 ENO) (6 FGAMS) (6 FUM) (7 GAPDH) (6 GARS) (6 GARTF) (4 GlcPTS) (9 GlPDH_Q) (6 Glu_Trans) (21 GluDH) (-12 GluS) (-3 GlyHMTrans) (3 GlyS) (6 ICitDH) (6 IMPCyc) (6 MDH) (24 Nitrogen_Trans) (4 PGI) (7 PGK) (4 PGM) (6 PPAT) (6 PRPPS) (3 PSAT) (3 PSerP) (4 R5PI) (-4 RPE) (6 SCAIRS) (-2 TA) (-2 TK1) (-2 TK2) (9 TPI)" |
| 59,"1.0","278","false","44","(3 3PGDH) (-6 Acetate_Trans) (6 ACN) (6 ACS) (12 AdenylatK) (6 ADSL) (6 AICARTF) (6 AIRC) (6 AIRS) (6 CitS) (4 ENO) (6 FGAMS) (6 FUM) (7 GAPDH) (6 GARS) (6 GARTF) (4 GlcPTS) (9 GlPDH_Q) (21 GluDH) (6 GluK) (-12 GluS) (6 GluSemiAld) (-3 GlyHMTrans) (3 GlyS) (6 ICitDH) (6 IMPCyc) (6 MDH) (24 Nitrogen_Trans) (4 PGI) (7 PGK) (4 PGM) (6 PPAT) (6 Pro_Trans) (6 ProO) (6 PRPPS) (3 PSAT) (3 PSerP) (4 R5PI) (-4 RPE) (6 SCAIRS) (-2 TA) (-2 TK1) (-2 TK2) (9 TPI)" |
| 60,"1.0","266","false","43","(3 3PGDH) (6 ACN) (6 AdenylatK) (6 ADSL) (6 AICARTF) (6 AIRC) (6 AIRS) (6 CitS) (4 ENO) (6 FGAMS) (6 FUM) (7 GAPDH) (6 GARS) (6 GARTF) (4 GlcPTS) (9 GlPDH_Q) (6 Glu_Trans) (21 GluDH) (-12 GluS) (-3 GlyHMTrans) (3 GlyS) (6 ICitDH) (6 IMPCyc) (6 MDH) (24 Nitrogen_Trans) (4 PGI) (7 PGK) (4 PGM) (6 PPAT) (6 PRPPS) (3 PSAT) (3 PSerP) (6 PyrDH_E1A) (6 PyrDH_E1B) (-6 PyrDH_E2) (6 PyrDH_E3) (4 R5PI) (-4 RPE) (6 SCAIRS) (-2 TA) (-2 TK1) (-2 TK2) (9 TPI)" |
| 61,"1.0","284","false","46","(3 3PGDH) (6 ACN) (6 AdenylatK) (6 ADSL) (6 AICARTF) (6 AIRC) (6 AIRS) (6 CitS) (4 ENO) (6 FGAMS) (6 FUM) (7 GAPDH) (6 GARS) (6 GARTF) (4 GlcPTS) (9 GlPDH_Q) (21 GluDH) (6 GluK) (-12 GluS) (6 GluSemiAld) (-3 GlyHMTrans) (3 GlyS) (6 ICitDH) (6 IMPCyc) (6 MDH) (24 Nitrogen_Trans) (4 PGI) (7 PGK) (4 PGM) (6 PPAT) (6 Pro_Trans) (6 ProO) (6 PRPPS) (3 PSAT) (3 PSerP) (6 PyrDH_E1A) (6 PyrDH_E1B) (-6 PyrDH_E2) (6 PyrDH_E3) (4 R5PI) (-4 RPE) (6 SCAIRS) (-2 TA) (-2 TK1) (-2 TK2) (9 TPI)" |
| 62,"1.0","272","false","43","(3 3PGDH) (-6 Acetate_Trans) (6 ACS) (12 AdenylatK) (6 ADSL) (6 AICARTF) (6 AIRC) (6 AIRS) (6 AspK) (-6 AspSemiAldDH) (6 AspTA) (6 DAPDC) (-6 DHDPCR) (6 DHDPCS) (4 FBP) (-4 FBPAld) (6 FGAMS) (6 FUM) (3 GAPDH) (6 GARS) (6 GARTF) (13 GlPDH_Q) (21 GluDH) (-12 GluS) (-3 GlyHMTrans) (3 GlyS) (6 IMPCyc) (6 Lys_Trans) (6 LysEC) (6 MDH) (24 Nitrogen_Trans) (3 PGK) (6 PPAT) (6 PRPPS) (3 PSAT) (3 PSerP) (4 R5PI) (-4 RPE) (6 SCAIRS) (-2 TA) (-2 TK1) (-2 TK2) (9 TPI)" |
| 63,"1.0","278","false","45","(3 3PGDH) (6 AdenylatK) (6 ADSL) (6 AICARTF) (6 AIRC) (6 AIRS) (6 AspK) (-6 AspSemiAldDH) (6 AspTA) (6 DAPDC) (-6 DHDPCR) (6 DHDPCS) (4 FBP) (-4 FBPAld) (6 FGAMS) (6 FUM) (3 GAPDH) (6 GARS) (6 GARTF) (13 GlPDH_Q) (21 GluDH) (-12 GluS) (-3 GlyHMTrans) (3 GlyS) (6 IMPCyc) (6 Lys_Trans) (6 LysEC) (6 MDH) (24 Nitrogen_Trans) (3 PGK) (6 PPAT) (6 PRPPS) (3 PSAT) (3 PSerP) (6 PyrDH_E1A) (6 PyrDH_E1B) (-6 PyrDH_E2) (6 PyrDH_E3) (4 R5PI) (-4 RPE) (6 SCAIRS) (-2 TA) (-2 TK1) (-2 TK2) (9 TPI)" |
| 64,"1.0","284","false","45","(3 3PGDH) (-6 Acetate_Trans) (6 ACS) (12 AdenylatK) (6 ADSL) (6 AICARTF) (6 AIRC) (6 AIRS) (6 AspK) (-6 AspSemiAldDH) (6 AspTA) (6 DAPDC) (-6 DHDPCR) (6 DHDPCS) (4 ENO) (6 FGAMS) (6 FUM) (7 GAPDH) (6 GARS) (6 GARTF) (4 GlcPTS) (9 GlPDH_Q) (21 GluDH) (-12 GluS) (-3 GlyHMTrans) (3 GlyS) (6 IMPCyc) (6 Lys_Trans) (6 LysEC) (6 MDH) (24 Nitrogen_Trans) (4 PGI) (7 PGK) (4 PGM) (6 PPAT) (6 PRPPS) (3 PSAT) (3 PSerP) (4 R5PI) (-4 RPE) (6 SCAIRS) (-2 TA) (-2 TK1) (-2 TK2) (9 TPI)" |
| 65,"1.0","290","false","47","(3 3PGDH) (6 AdenylatK) (6 ADSL) (6 AICARTF) (6 AIRC) (6 AIRS) (6 AspK) (-6 AspSemiAldDH) (6 AspTA) (6 DAPDC) (-6 DHDPCR) (6 DHDPCS) (4 ENO) (6 FGAMS) (6 FUM) (7 GAPDH) (6 GARS) (6 GARTF) (4 GlcPTS) (9 GlPDH_Q) (21 GluDH) (-12 GluS) (-3 GlyHMTrans) (3 GlyS) (6 IMPCyc) (6 Lys_Trans) (6 LysEC) (6 MDH) (24 Nitrogen_Trans) (4 PGI) (7 PGK) (4 PGM) (6 PPAT) (6 PRPPS) (3 PSAT) (3 PSerP) (6 PyrDH_E1A) (6 PyrDH_E1B) (-6 PyrDH_E2) (6 PyrDH_E3) (4 R5PI) (-4 RPE) (6 SCAIRS) (-2 TA) (-2 TK1) (-2 TK2) (9 TPI)" |
| 66,"1.0","194","false","33","(3 3PGDH) (6 AdenylatK) (6 ADSL) (6 AICARTF) (6 AIRC) (6 AIRS) (4 FBP) (-4 FBPAld) (6 FGAMS) (3 GAPDH) (6 GARS) (6 GARTF) (13 GlPDH_Q) (15 GluDH) (-12 GluS) (-3 GlyHMTrans) (3 GlyS) (6 IMPCyc) (18 Nitrogen_Trans) (3 PGK) (6 PPAT) (6 PRPPS) (3 PSAT) (3 PSerP) (4 R5PI) (-4 RPE) (6 SCAIRS) (6 Succ_Trans) (-6 SuccDH) (-2 TA) (-2 TK1) (-2 TK2) (9 TPI)" |
| 67,"1.0","206","false","35","(3 3PGDH) (6 AdenylatK) (6 ADSL) (6 AICARTF) (6 AIRC) (6 AIRS) (4 ENO) (6 FGAMS) (7 GAPDH) (6 GARS) (6 GARTF) (4 GlcPTS) (9 GlPDH_Q) (15 GluDH) (-12 GluS) (-3 GlyHMTrans) (3 GlyS) (6 IMPCyc) (18 Nitrogen_Trans) (4 PGI) (7 PGK) (4 PGM) (6 PPAT) (6 PRPPS) (3 PSAT) (3 PSerP) (4 R5PI) (-4 RPE) (6 SCAIRS) (6 Succ_Trans) (-6 SuccDH) (-2 TA) (-2 TK1) (-2 TK2) (9 TPI)" |
| 68,"1.0","242","false","40","(3 3PGDH) (6 ACN) (6 AdenylatK) (6 ADSL) (6 AICARTF) (6 AIRC) (6 AIRS) (6 AldDH) (6 CitS) (6 EthanolamineAmmoniaLya) (4 FBP) (-4 FBPAld) (6 FGAMS) (6 FUM) (3 GAPDH) (6 GARS) (6 GARTF) (13 GlPDH_Q) (6 Glu_Trans) (21 GluDH) (-12 GluS) (6 GlycerophosphodiesterPdisterase) (-3 GlyHMTrans) (3 GlyS) (6 ICitDH) (6 IMPCyc) (6 MDH) (18 Nitrogen_Trans) (3 PGK) (6 PPAT) (6 PRPPS) (3 PSAT) (3 PSerP) (4 R5PI) (-4 RPE) (6 SCAIRS) (-2 TA) (-2 TK1) (-2 TK2) (9 TPI)" |
| 69,"1.0","260","false","43","(3 3PGDH) (6 ACN) (6 AdenylatK) (6 ADSL) (6 AICARTF) (6 AIRC) (6 AIRS) (6 AldDH) (6 CitS) (6 EthanolamineAmmoniaLya) (4 FBP) (-4 FBPAld) (6 FGAMS) (6 FUM) (3 GAPDH) (6 GARS) (6 GARTF) (13 GlPDH_Q) (21 GluDH) (6 GluK) (-12 GluS) (6 GluSemiAld) (6 GlycerophosphodiesterPdisterase) (-3 GlyHMTrans) (3 GlyS) (6 ICitDH) (6 IMPCyc) (6 MDH) (18 Nitrogen_Trans) (3 PGK) (6 PPAT) (6 Pro_Trans) (6 ProO) (6 PRPPS) (3 PSAT) (3 PSerP) (4 R5PI) (-4 RPE) (6 SCAIRS) (-2 TA) (-2 TK1) (-2 TK2) (9 TPI)" |
| 70,"1.0","254","false","42","(3 3PGDH) (6 ACN) (6 AdenylatK) (6 ADSL) (6 AICARTF) (6 AIRC) (6 AIRS) (6 AldDH) (6 CitS) (4 ENO) (6 EthanolamineAmmoniaLya) (6 FGAMS) (6 FUM) (7 GAPDH) (6 GARS) (6 GARTF) (4 GlcPTS) (9 GlPDH_Q) (6 Glu_Trans) (21 GluDH) (-12 GluS) (6 GlycerophosphodiesterPdisterase) (-3 GlyHMTrans) (3 GlyS) (6 ICitDH) (6 IMPCyc) (6 MDH) (18 Nitrogen_Trans) (4 PGI) (7 PGK) (4 PGM) (6 PPAT) (6 PRPPS) (3 PSAT) (3 PSerP) (4 R5PI) (-4 RPE) (6 SCAIRS) (-2 TA) (-2 TK1) (-2 TK2) (9 TPI)" |
| 71,"1.0","272","false","45","(3 3PGDH) (6 ACN) (6 AdenylatK) (6 ADSL) (6 AICARTF) (6 AIRC) (6 AIRS) (6 AldDH) (6 CitS) (4 ENO) (6 EthanolamineAmmoniaLya) (6 FGAMS) (6 FUM) (7 GAPDH) (6 GARS) (6 GARTF) (4 GlcPTS) (9 GlPDH_Q) (21 GluDH) (6 GluK) (-12 GluS) (6 GluSemiAld) (6 GlycerophosphodiesterPdisterase) (-3 GlyHMTrans) (3 GlyS) (6 ICitDH) (6 IMPCyc) (6 MDH) (18 Nitrogen_Trans) (4 PGI) (7 PGK) (4 PGM) (6 PPAT) (6 Pro_Trans) (6 ProO) (6 PRPPS) (3 PSAT) (3 PSerP) (4 R5PI) (-4 RPE) (6 SCAIRS) (-2 TA) (-2 TK1) (-2 TK2) (9 TPI)" |
| 72,"1.0","266","false","44","(3 3PGDH) (6 AdenylatK) (6 ADSL) (6 AICARTF) (6 AIRC) (6 AIRS) (6 AldDH) (6 AspK) (-6 AspSemiAldDH) (6 AspTA) (6 DAPDC) (-6 DHDPCR) (6 DHDPCS) (6 EthanolamineAmmoniaLya) (4 FBP) (-4 FBPAld) (6 FGAMS) (6 FUM) (3 GAPDH) (6 GARS) (6 GARTF) (13 GlPDH_Q) (21 GluDH) (-12 GluS) (6 GlycerophosphodiesterPdisterase) (-3 GlyHMTrans) (3 GlyS) (6 IMPCyc) (6 Lys_Trans) (6 LysEC) (6 MDH) (18 Nitrogen_Trans) (3 PGK) (6 PPAT) (6 PRPPS) (3 PSAT) (3 PSerP) (4 R5PI) (-4 RPE) (6 SCAIRS) (-2 TA) (-2 TK1) (-2 TK2) (9 TPI)" |
| 73,"1.0","278","false","46","(3 3PGDH) (6 AdenylatK) (6 ADSL) (6 AICARTF) (6 AIRC) (6 AIRS) (6 AldDH) (6 AspK) (-6 AspSemiAldDH) (6 AspTA) (6 DAPDC) (-6 DHDPCR) (6 DHDPCS) (4 ENO) (6 EthanolamineAmmoniaLya) (6 FGAMS) (6 FUM) (7 GAPDH) (6 GARS) (6 GARTF) (4 GlcPTS) (9 GlPDH_Q) (21 GluDH) (-12 GluS) (6 GlycerophosphodiesterPdisterase) (-3 GlyHMTrans) (3 GlyS) (6 IMPCyc) (6 Lys_Trans) (6 LysEC) (6 MDH) (18 Nitrogen_Trans) (4 PGI) (7 PGK) (4 PGM) (6 PPAT) (6 PRPPS) (3 PSAT) (3 PSerP) (4 R5PI) (-4 RPE) (6 SCAIRS) (-2 TA) (-2 TK1) (-2 TK2) (9 TPI)" |
| 74,"1.0","9","false","9","(1 3PGDH) (1 GAPDH) (1 GlPDH_Q) (1 GluDH) (1 PGK) (1 PSAT) (1 PSerP) (1 SerDHA) (1 TPI)" |
| 75,"1.0","10","false","10","(1 3PGDH) (1 GAPDH) (1 GlPDH_Q) (1 GluDH) (1 Nitrogen_Trans) (1 PGK) (1 PSAT) (1 PSerP) (1 Ser_Trans) (1 TPI)" |
| 76,"1.0","17","false","17","(1 3PGDH) (1 Acetate_Trans) (1 Cit_Trans) (-1 CitS) (1 Cys_trans) (1 CysS) (1 GAPDH) (1 GlPDH_Q) (1 GluDH) (1 Mae) (-1 MDH) (1 Nitrogen_Trans) (1 PGK) (1 PSAT) (1 PSerP) (1 SerTAc) (1 TPI)" |
| 77,"1.0","194","false","33","(3 3PGDH) (6 AdenylatK) (6 ADSL) (6 AICARTF) (6 AIRC) (6 AIRS) (4 FBP) (-4 FBPAld) (6 FGAMS) (6 FUM) (3 GAPDH) (6 GARS) (6 GARTF) (13 GlPDH_Q) (15 GluDH) (-12 GluS) (-3 GlyHMTrans) (3 GlyS) (6 IMPCyc) (6 Mae) (18 Nitrogen_Trans) (3 PGK) (6 PPAT) (6 PRPPS) (3 PSAT) (3 PSerP) (4 R5PI) (-4 RPE) (6 SCAIRS) (-2 TA) (-2 TK1) (-2 TK2) (9 TPI)" |
| 78,"1.0","206","false","35","(3 3PGDH) (6 AdenylatK) (6 ADSL) (6 AICARTF) (6 AIRC) (6 AIRS) (4 ENO) (6 FGAMS) (6 FUM) (7 GAPDH) (6 GARS) (6 GARTF) (4 GlcPTS) (9 GlPDH_Q) (15 GluDH) (-12 GluS) (-3 GlyHMTrans) (3 GlyS) (6 IMPCyc) (6 Mae) (18 Nitrogen_Trans) (4 PGI) (7 PGK) (4 PGM) (6 PPAT) (6 PRPPS) (3 PSAT) (3 PSerP) (4 R5PI) (-4 RPE) (6 SCAIRS) (-2 TA) (-2 TK1) (-2 TK2) (9 TPI)" |
| 79,"1.0","362","false","48","(3 3PGDH) (-6 Acetate_Trans) (6 AcGluK) (-6 AcGluR) (6 ACN) (-6 AcOrnTA) (6 ACS) (18 AdenylatK) (6 ADSL) (6 AICARTF) (6 AIRC) (6 AIRS) (6 Arg_Trans) (6 ArgSucc) (6 ArgSuccS) (6 AspTA) (6 CarbPS) (6 CitS) (4 FBP) (-4 FBPAld) (6 FGAMS) (12 FUM) (3 GAPDH) (6 GARS) (6 GARTF) (13 GlPDH_Q) (39 GluDH) (-18 GluS) (6 GlutAcetylTrans) (-3 GlyHMTrans) (3 GlyS) (6 ICitDH) (6 IMPCyc) (12 MDH) (42 Nitrogen_Trans) (6 OrnCarbTrans) (3 PGK) (6 PPAT) (6 PRPPS) (3 PSAT) (3 PSerP) (4 R5PI) (-4 RPE) (6 SCAIRS) (-2 TA) (-2 TK1) (-2 TK2) (9 TPI)" |
| 80,"1.0","368","false","50","(3 3PGDH) (6 AcGluK) (-6 AcGluR) (6 ACN) (-6 AcOrnTA) (12 AdenylatK) (6 ADSL) (6 AICARTF) (6 AIRC) (6 AIRS) (6 Arg_Trans) (6 ArgSucc) (6 ArgSuccS) (6 AspTA) (6 CarbPS) (6 CitS) (4 FBP) (-4 FBPAld) (6 FGAMS) (12 FUM) (3 GAPDH) (6 GARS) (6 GARTF) (13 GlPDH_Q) (39 GluDH) (-18 GluS) (6 GlutAcetylTrans) (-3 GlyHMTrans) (3 GlyS) (6 ICitDH) (6 IMPCyc) (12 MDH) (42 Nitrogen_Trans) (6 OrnCarbTrans) (3 PGK) (6 PPAT) (6 PRPPS) (3 PSAT) (3 PSerP) (6 PyrDH_E1A) (6 PyrDH_E1B) (-6 PyrDH_E2) (6 PyrDH_E3) (4 R5PI) (-4 RPE) (6 SCAIRS) (-2 TA) (-2 TK1) (-2 TK2) (9 TPI)" |
| 81,"1.0","374","false","50","(3 3PGDH) (-6 Acetate_Trans) (6 AcGluK) (-6 AcGluR) (6 ACN) (-6 AcOrnTA) (6 ACS) (18 AdenylatK) (6 ADSL) (6 AICARTF) (6 AIRC) (6 AIRS) (6 Arg_Trans) (6 ArgSucc) (6 ArgSuccS) (6 AspTA) (6 CarbPS) (6 CitS) (4 ENO) (6 FGAMS) (12 FUM) (7 GAPDH) (6 GARS) (6 GARTF) (4 GlcPTS) (9 GlPDH_Q) (39 GluDH) (-18 GluS) (6 GlutAcetylTrans) (-3 GlyHMTrans) (3 GlyS) (6 ICitDH) (6 IMPCyc) (12 MDH) (42 Nitrogen_Trans) (6 OrnCarbTrans) (4 PGI) (7 PGK) (4 PGM) (6 PPAT) (6 PRPPS) (3 PSAT) (3 PSerP) (4 R5PI) (-4 RPE) (6 SCAIRS) (-2 TA) (-2 TK1) (-2 TK2) (9 TPI)" |
| 82,"1.0","380","false","52","(3 3PGDH) (6 AcGluK) (-6 AcGluR) (6 ACN) (-6 AcOrnTA) (12 AdenylatK) (6 ADSL) (6 AICARTF) (6 AIRC) (6 AIRS) (6 Arg_Trans) (6 ArgSucc) (6 ArgSuccS) (6 AspTA) (6 CarbPS) (6 CitS) (4 ENO) (6 FGAMS) (12 FUM) (7 GAPDH) (6 GARS) (6 GARTF) (4 GlcPTS) (9 GlPDH_Q) (39 GluDH) (-18 GluS) (6 GlutAcetylTrans) (-3 GlyHMTrans) (3 GlyS) (6 ICitDH) (6 IMPCyc) (12 MDH) (42 Nitrogen_Trans) (6 OrnCarbTrans) (4 PGI) (7 PGK) (4 PGM) (6 PPAT) (6 PRPPS) (3 PSAT) (3 PSerP) (6 PyrDH_E1A) (6 PyrDH_E1B) (-6 PyrDH_E2) (6 PyrDH_E3) (4 R5PI) (-4 RPE) (6 SCAIRS) (-2 TA) (-2 TK1) (-2 TK2) (9 TPI)" |
| 83,"1.0","356","false","49","(3 3PGDH) (6 AcGluK) (-6 AcGluR) (6 ACN) (-6 AcOrnTA) (12 AdenylatK) (6 ADSL) (6 AICARTF) (6 AIRC) (6 AIRS) (6 AldDH) (6 Arg_Trans) (6 ArgSucc) (6 ArgSuccS) (6 AspTA) (6 CarbPS) (6 CitS) (6 EthanolamineAmmoniaLya) (4 FBP) (-4 FBPAld) (6 FGAMS) (12 FUM) (3 GAPDH) (6 GARS) (6 GARTF) (13 GlPDH_Q) (39 GluDH) (-18 GluS) (6 GlutAcetylTrans) (6 GlycerophosphodiesterPdisterase) (-3 GlyHMTrans) (3 GlyS) (6 ICitDH) (6 IMPCyc) (12 MDH) (36 Nitrogen_Trans) (6 OrnCarbTrans) (3 PGK) (6 PPAT) (6 PRPPS) (3 PSAT) (3 PSerP) (4 R5PI) (-4 RPE) (6 SCAIRS) (-2 TA) (-2 TK1) (-2 TK2) (9 TPI)" |
| 84,"1.0","368","false","51","(3 3PGDH) (6 AcGluK) (-6 AcGluR) (6 ACN) (-6 AcOrnTA) (12 AdenylatK) (6 ADSL) (6 AICARTF) (6 AIRC) (6 AIRS) (6 AldDH) (6 Arg_Trans) (6 ArgSucc) (6 ArgSuccS) (6 AspTA) (6 CarbPS) (6 CitS) (4 ENO) (6 EthanolamineAmmoniaLya) (6 FGAMS) (12 FUM) (7 GAPDH) (6 GARS) (6 GARTF) (4 GlcPTS) (9 GlPDH_Q) (39 GluDH) (-18 GluS) (6 GlutAcetylTrans) (6 GlycerophosphodiesterPdisterase) (-3 GlyHMTrans) (3 GlyS) (6 ICitDH) (6 IMPCyc) (12 MDH) (36 Nitrogen_Trans) (6 OrnCarbTrans) (4 PGI) (7 PGK) (4 PGM) (6 PPAT) (6 PRPPS) (3 PSAT) (3 PSerP) (4 R5PI) (-4 RPE) (6 SCAIRS) (-2 TA) (-2 TK1) (-2 TK2) (9 TPI)" |
| 85,"1.0","5","false","5","(1 FucI) (1 Fucose_Trans) (1 FucuK) (1 FucuPAld) (-1 GlPDH_Q)" |
| 86,"1.0","6","false","6","(1 AcLacS) (1 GluDH) (1 Nitrogen_Trans) (1 TA_B_Val) (1 Val_EC) (1 Val_Trans)" |
| 87,"1.0","8","false","7","(1 FBP) (-1 FBPAld) (1 Glc6P) (1 Glc_Trans) (2 GlPDH_Q) (-1 PGI) (1 TPI)" |
| 88,"1.0","9","false","9","(1 ENO) (1 GAPDH) (1 Glc6P) (1 Glc_Trans) (1 GlcPTS) (1 GlPDH_Q) (1 PGK) (1 PGM) (1 TPI)" |
| 89,"1.0","90","false","26","(3 ChorM) (3 ChorS) (3 DAHPS) (3 DHQDA) (3 DHQS) (6 ENO) (3 EPSPS) (1 FBP) (-1 FBPAld) (6 GAPDH) (10 GlPDH_Q) (3 GluDH) (3 Nitrogen_Trans) (6 PGK) (6 PGM) (3 Phe_Trans) (-3 PheTA) (3 PrephenateDA) (1 R5PI) (-1 RPE) (-3 ShiDH) (3 ShiK) (1 TA) (1 TK1) (-2 TK2) (9 TPI)" |
| 90,"1.0","93","false","26","(3 ChorM) (3 ChorS) (3 DAHPS) (3 DHQDA) (3 DHQS) (7 ENO) (3 EPSPS) (7 GAPDH) (1 GlcPTS) (9 GlPDH_Q) (3 GluDH) (3 Nitrogen_Trans) (1 PGI) (7 PGK) (7 PGM) (3 Phe_Trans) (-3 PheTA) (3 PrephenateDA) (1 R5PI) (-1 RPE) (-3 ShiDH) (3 ShiK) (1 TA) (1 TK1) (-2 TK2) (9 TPI)" |
| 91,"1.0","90","false","26","(3 ChorM) (3 ChorS) (3 DAHPS) (3 DHQDA) (3 DHQS) (6 ENO) (3 EPSPS) (1 FBP) (-1 FBPAld) (6 GAPDH) (10 GlPDH_Q) (3 GluDH) (3 Nitrogen_Trans) (6 PGK) (6 PGM) (3 PrephenateDH) (1 R5PI) (-1 RPE) (-3 ShiDH) (3 ShiK) (1 TA) (1 TK1) (-2 TK2) (9 TPI) (3 Tyr_Trans) (-3 TyrTA)" |
| 92,"1.0","93","false","26","(3 ChorM) (3 ChorS) (3 DAHPS) (3 DHQDA) (3 DHQS) (7 ENO) (3 EPSPS) (7 GAPDH) (1 GlcPTS) (9 GlPDH_Q) (3 GluDH) (3 Nitrogen_Trans) (1 PGI) (7 PGK) (7 PGM) (3 PrephenateDH) (1 R5PI) (-1 RPE) (-3 ShiDH) (3 ShiK) (1 TA) (1 TK1) (-2 TK2) (9 TPI) (3 Tyr_Trans) (-3 TyrTA)" |
| 93,"1.0","14","false","12","(1 Acetate_Trans) (1 AckA) (1 AdenylatK) (1 Asn_Trans) (1 AsnS) (1 AspTA) (1 Cit_Trans) (-1 CitS) (2 GluDH) (-1 GluS) (2 Nitrogen_Trans) (1 PTA)" |
| 94,"1.0","10","false","8","(1 AdenylatK) (1 Asn_Trans) (1 AsnS) (1 AspTA) (2 GluDH) (-1 GluS) (2 Nitrogen_Trans) (1 PyrCO)" |
| 95,"1.0","24","false","20","(1 3PGDH) (1 Acetate_Trans) (1 AdenylatK) (1 Asn_Trans) (1 AsnS) (1 AspTA) (1 Cit_Trans) (-1 CitS) (1 Cys_trans) (1 CysS) (1 GAPDH) (1 GlPDH_Q) (3 GluDH) (-1 GluS) (3 Nitrogen_Trans) (1 PGK) (1 PSAT) (1 PSerP) (1 SerTAc) (1 TPI)" |
| 96,"1.0","248","false","36","(3 3PGDH) (12 AdenylatK) (6 ADSL) (6 AICARTF) (6 AIRC) (6 AIRS) (6 Asn_Trans) (6 AsnS) (6 AspTA) (4 FBP) (-4 FBPAld) (6 FGAMS) (6 FUM) (3 GAPDH) (6 GARS) (6 GARTF) (13 GlPDH_Q) (27 GluDH) (-18 GluS) (-3 GlyHMTrans) (3 GlyS) (6 IMPCyc) (6 MDH) (30 Nitrogen_Trans) (3 PGK) (6 PPAT) (6 PRPPS) (3 PSAT) (3 PSerP) (4 R5PI) (-4 RPE) (6 SCAIRS) (-2 TA) (-2 TK1) (-2 TK2) (9 TPI)" |
| 97,"1.0","260","false","38","(3 3PGDH) (12 AdenylatK) (6 ADSL) (6 AICARTF) (6 AIRC) (6 AIRS) (6 Asn_Trans) (6 AsnS) (6 AspTA) (4 ENO) (6 FGAMS) (6 FUM) (7 GAPDH) (6 GARS) (6 GARTF) (4 GlcPTS) (9 GlPDH_Q) (27 GluDH) (-18 GluS) (-3 GlyHMTrans) (3 GlyS) (6 IMPCyc) (6 MDH) (30 Nitrogen_Trans) (4 PGI) (7 PGK) (4 PGM) (6 PPAT) (6 PRPPS) (3 PSAT) (3 PSerP) (4 R5PI) (-4 RPE) (6 SCAIRS) (-2 TA) (-2 TK1) (-2 TK2) (9 TPI)" |
| 98,"1.0","23","false","18","(1 AcGluK) (-1 AcGluR) (1 ACN) (-1 AcOrnTA) (1 AdenylatK) (1 Arg_Trans) (1 ArgSucc) (1 ArgSuccS) (1 AspTA) (1 CarbK) (1 Cit_Trans) (1 FUM) (3 GluDH) (1 GlutAcetylTrans) (1 ICitDH) (1 MDH) (4 Nitrogen_Trans) (1 OrnCarbTrans)" |
| 99,"1.0","27","false","21","(-1 Acetate_Trans) (1 AcGluK) (-1 AcGluR) (1 ACN) (-1 AcOrnTA) (1 ACS) (2 AdenylatK) (1 Arg_Trans) (1 ArgSucc) (1 ArgSuccS) (1 AspTA) (1 CarbK) (1 CitS) (1 FUM) (3 GluDH) (1 GlutAcetylTrans) (1 ICitDH) (1 MDH) (4 Nitrogen_Trans) (1 OrnCarbTrans) (1 PyrCO)" |
| 100,"1.0","28","false","23","(1 AcGluK) (-1 AcGluR) (1 ACN) (-1 AcOrnTA) (1 AdenylatK) (1 Arg_Trans) (1 ArgSucc) (1 ArgSuccS) (1 AspTA) (1 CarbK) (1 CitS) (1 FUM) (3 GluDH) (1 GlutAcetylTrans) (1 ICitDH) (1 MDH) (4 Nitrogen_Trans) (1 OrnCarbTrans) (1 PyrCO) (1 PyrDH_E1A) (1 PyrDH_E1B) (-1 PyrDH_E2) (1 PyrDH_E3)" |
| 101,"1.0","26","false","22","(1 AcGluK) (-1 AcGluR) (1 ACN) (-1 AcOrnTA) (1 AdenylatK) (1 AldDH) (1 Arg_Trans) (1 ArgSucc) (1 ArgSuccS) (1 AspTA) (1 CarbK) (1 CitS) (1 EthanolamineAmmoniaLya) (1 FUM) (3 GluDH) (1 GlutAcetylTrans) (1 GlycerophosphodiesterPdisterase) (1 ICitDH) (1 MDH) (3 Nitrogen_Trans) (1 OrnCarbTrans) (1 PyrCO)" |
| 102,"1.0","350","false","48","(3 3PGDH) (-6 Acetate_Trans) (6 AcGluK) (-6 AcGluR) (6 ACN) (-6 AcOrnTA) (6 ACS) (18 AdenylatK) (6 ADSL) (6 AICARTF) (6 AIRC) (6 AIRS) (6 Arg_Trans) (6 ArgSucc) (6 ArgSuccS) (6 AspTA) (6 CarbK) (6 CitS) (4 FBP) (-4 FBPAld) (6 FGAMS) (12 FUM) (3 GAPDH) (6 GARS) (6 GARTF) (13 GlPDH_Q) (33 GluDH) (-12 GluS) (6 GlutAcetylTrans) (-3 GlyHMTrans) (3 GlyS) (6 ICitDH) (6 IMPCyc) (12 MDH) (42 Nitrogen_Trans) (6 OrnCarbTrans) (3 PGK) (6 PPAT) (6 PRPPS) (3 PSAT) (3 PSerP) (4 R5PI) (-4 RPE) (6 SCAIRS) (-2 TA) (-2 TK1) (-2 TK2) (9 TPI)" |
| 103,"1.0","356","false","50","(3 3PGDH) (6 AcGluK) (-6 AcGluR) (6 ACN) (-6 AcOrnTA) (12 AdenylatK) (6 ADSL) (6 AICARTF) (6 AIRC) (6 AIRS) (6 Arg_Trans) (6 ArgSucc) (6 ArgSuccS) (6 AspTA) (6 CarbK) (6 CitS) (4 FBP) (-4 FBPAld) (6 FGAMS) (12 FUM) (3 GAPDH) (6 GARS) (6 GARTF) (13 GlPDH_Q) (33 GluDH) (-12 GluS) (6 GlutAcetylTrans) (-3 GlyHMTrans) (3 GlyS) (6 ICitDH) (6 IMPCyc) (12 MDH) (42 Nitrogen_Trans) (6 OrnCarbTrans) (3 PGK) (6 PPAT) (6 PRPPS) (3 PSAT) (3 PSerP) (6 PyrDH_E1A) (6 PyrDH_E1B) (-6 PyrDH_E2) (6 PyrDH_E3) (4 R5PI) (-4 RPE) (6 SCAIRS) (-2 TA) (-2 TK1) (-2 TK2) (9 TPI)" |
| 104,"1.0","362","false","50","(3 3PGDH) (-6 Acetate_Trans) (6 AcGluK) (-6 AcGluR) (6 ACN) (-6 AcOrnTA) (6 ACS) (18 AdenylatK) (6 ADSL) (6 AICARTF) (6 AIRC) (6 AIRS) (6 Arg_Trans) (6 ArgSucc) (6 ArgSuccS) (6 AspTA) (6 CarbK) (6 CitS) (4 ENO) (6 FGAMS) (12 FUM) (7 GAPDH) (6 GARS) (6 GARTF) (4 GlcPTS) (9 GlPDH_Q) (33 GluDH) (-12 GluS) (6 GlutAcetylTrans) (-3 GlyHMTrans) (3 GlyS) (6 ICitDH) (6 IMPCyc) (12 MDH) (42 Nitrogen_Trans) (6 OrnCarbTrans) (4 PGI) (7 PGK) (4 PGM) (6 PPAT) (6 PRPPS) (3 PSAT) (3 PSerP) (4 R5PI) (-4 RPE) (6 SCAIRS) (-2 TA) (-2 TK1) (-2 TK2) (9 TPI)" |
| 105,"1.0","368","false","52","(3 3PGDH) (6 AcGluK) (-6 AcGluR) (6 ACN) (-6 AcOrnTA) (12 AdenylatK) (6 ADSL) (6 AICARTF) (6 AIRC) (6 AIRS) (6 Arg_Trans) (6 ArgSucc) (6 ArgSuccS) (6 AspTA) (6 CarbK) (6 CitS) (4 ENO) (6 FGAMS) (12 FUM) (7 GAPDH) (6 GARS) (6 GARTF) (4 GlcPTS) (9 GlPDH_Q) (33 GluDH) (-12 GluS) (6 GlutAcetylTrans) (-3 GlyHMTrans) (3 GlyS) (6 ICitDH) (6 IMPCyc) (12 MDH) (42 Nitrogen_Trans) (6 OrnCarbTrans) (4 PGI) (7 PGK) (4 PGM) (6 PPAT) (6 PRPPS) (3 PSAT) (3 PSerP) (6 PyrDH_E1A) (6 PyrDH_E1B) (-6 PyrDH_E2) (6 PyrDH_E3) (4 R5PI) (-4 RPE) (6 SCAIRS) (-2 TA) (-2 TK1) (-2 TK2) (9 TPI)" |
| 106,"1.0","344","false","49","(3 3PGDH) (6 AcGluK) (-6 AcGluR) (6 ACN) (-6 AcOrnTA) (12 AdenylatK) (6 ADSL) (6 AICARTF) (6 AIRC) (6 AIRS) (6 AldDH) (6 Arg_Trans) (6 ArgSucc) (6 ArgSuccS) (6 AspTA) (6 CarbK) (6 CitS) (6 EthanolamineAmmoniaLya) (4 FBP) (-4 FBPAld) (6 FGAMS) (12 FUM) (3 GAPDH) (6 GARS) (6 GARTF) (13 GlPDH_Q) (33 GluDH) (-12 GluS) (6 GlutAcetylTrans) (6 GlycerophosphodiesterPdisterase) (-3 GlyHMTrans) (3 GlyS) (6 ICitDH) (6 IMPCyc) (12 MDH) (36 Nitrogen_Trans) (6 OrnCarbTrans) (3 PGK) (6 PPAT) (6 PRPPS) (3 PSAT) (3 PSerP) (4 R5PI) (-4 RPE) (6 SCAIRS) (-2 TA) (-2 TK1) (-2 TK2) (9 TPI)" |
| 107,"1.0","356","false","51","(3 3PGDH) (6 AcGluK) (-6 AcGluR) (6 ACN) (-6 AcOrnTA) (12 AdenylatK) (6 ADSL) (6 AICARTF) (6 AIRC) (6 AIRS) (6 AldDH) (6 Arg_Trans) (6 ArgSucc) (6 ArgSuccS) (6 AspTA) (6 CarbK) (6 CitS) (4 ENO) (6 EthanolamineAmmoniaLya) (6 FGAMS) (12 FUM) (7 GAPDH) (6 GARS) (6 GARTF) (4 GlcPTS) (9 GlPDH_Q) (33 GluDH) (-12 GluS) (6 GlutAcetylTrans) (6 GlycerophosphodiesterPdisterase) (-3 GlyHMTrans) (3 GlyS) (6 ICitDH) (6 IMPCyc) (12 MDH) (36 Nitrogen_Trans) (6 OrnCarbTrans) (4 PGI) (7 PGK) (4 PGM) (6 PPAT) (6 PRPPS) (3 PSAT) (3 PSerP) (4 R5PI) (-4 RPE) (6 SCAIRS) (-2 TA) (-2 TK1) (-2 TK2) (9 TPI)" |
| 108,"1.0","13","false","11","(1 AcLacS) (1 Asp_Trans) (1 AspTA) (1 Cit_Trans) (-1 CitS) (2 GluDH) (1 Leu_EC) (1 Leu_Trans) (2 Nitrogen_Trans) (1 TA_B_Leu) (1 Val_EC)" |
| 109,"1.0","18","false","16","(1 AcLacS) (1 AspK) (-1 AspSemiAldDH) (1 AspTA) (1 Cit_Trans) (-1 CitS) (2 GluDH) (-1 HomoSerDH) (1 HomoSerK) (1 Leu_EC) (1 Leu_Trans) (2 Nitrogen_Trans) (1 TA_B_Leu) (1 Thr_Trans) (1 ThrS) (1 Val_EC)" |
| 110,"1.0","23","false","20","(1 AcLacS) (1 AcLacS2) (1 AspK) (-1 AspSemiAldDH) (1 AspTA) (1 Cit_Trans) (-1 CitS) (3 GluDH) (-1 HomoSerDH) (1 HomoSerK) (1 Ile_EC) (1 Ile_Trans) (1 Leu_EC) (1 Leu_Trans) (2 Nitrogen_Trans) (1 TA_B_Ile) (1 TA_B_Leu) (1 ThrDHA) (1 ThrS) (1 Val_EC)" |
| 111,"1.0","10","false","10","(-1 Acetate_Trans) (1 AcLacS) (1 ACS) (1 AdenylatK) (1 GluDH) (1 Leu_EC) (1 Leu_Trans) (1 Nitrogen_Trans) (1 TA_B_Leu) (1 Val_EC)" |
| 112,"1.0","11","false","11","(1 AcLacS) (1 GluDH) (1 Leu_EC) (1 Leu_Trans) (1 Nitrogen_Trans) (1 PyrDH_E1A) (1 PyrDH_E1B) (-1 PyrDH_E2) (1 PyrDH_E3) (1 TA_B_Leu) (1 Val_EC)" |
| 113,"1.0","13","false","13","(1 AcLacS) (1 Cit_Trans) (-1 CitS) (-1 FUM) (1 GluDH) (1 Leu_EC) (1 Leu_Trans) (-1 MDH) (1 Nitrogen_Trans) (1 Succ_Trans) (-1 SuccDH) (1 TA_B_Leu) (1 Val_EC)" |
| 114,"1.0","9","false","9","(1 AcLacS) (1 AldDH) (1 EthanolamineAmmoniaLya) (1 GluDH) (1 GlycerophosphodiesterPdisterase) (1 Leu_EC) (1 Leu_Trans) (1 TA_B_Leu) (1 Val_EC)" |
| 115,"1.0","11","false","11","(1 AcLacS) (1 Cit_Trans) (-1 CitS) (1 GluDH) (1 Leu_EC) (1 Leu_Trans) (1 Mae) (-1 MDH) (1 Nitrogen_Trans) (1 TA_B_Leu) (1 Val_EC)" |
| 116,"1.0","18","false","14","(1 AcLacS) (1 AdenylatK) (1 Asn_Trans) (1 AsnS) (1 AspTA) (1 Cit_Trans) (-1 CitS) (3 GluDH) (-1 GluS) (1 Leu_EC) (1 Leu_Trans) (3 Nitrogen_Trans) (1 TA_B_Leu) (1 Val_EC)" |
| 117,"1.0","3","false","3","(1 GlnS) (-1 GluDH) (1 GluS)" |
| 118,"1.0","21","false","12","(3 6PGDH) (3 6PGL) (1 FBP) (-1 FBPAld) (3 G6PDH) (1 GlPDH_Q) (-3 PGI) (1 R5PI) (2 RPE) (1 TA) (1 TK1) (1 TK2)" |
| 119,"1.0","22","false","14","(3 6PGDH) (3 6PGL) (1 ENO) (3 G6PDH) (1 GAPDH) (1 GlcPTS) (-2 PGI) (1 PGK) (1 PGM) (1 R5PI) (2 RPE) (1 TA) (1 TK1) (1 TK2)" |
| 120,"1.0","9","false","7","(1 ACN) (1 Cit_Trans) (1 Gln_Trans) (2 GluDH) (-1 GluS) (1 ICitDH) (2 Nitrogen_Trans)" |
| 121,"1.0","13","false","11","(-1 Acetate_Trans) (1 ACN) (1 ACS) (1 AdenylatK) (1 CitS) (1 Gln_Trans) (2 GluDH) (-1 GluS) (1 ICitDH) (2 Nitrogen_Trans) (1 PyrCO)" |
| 122,"1.0","14","false","12","(1 ACN) (1 CitS) (1 Gln_Trans) (2 GluDH) (-1 GluS) (1 ICitDH) (2 Nitrogen_Trans) (1 PyrCO) (1 PyrDH_E1A) (1 PyrDH_E1B) (-1 PyrDH_E2) (1 PyrDH_E3)" |
| 123,"1.0","12","false","11","(1 ACN) (1 AldDH) (1 CitS) (1 EthanolamineAmmoniaLya) (1 Gln_Trans) (2 GluDH) (-1 GluS) (1 GlycerophosphodiesterPdisterase) (1 ICitDH) (1 Nitrogen_Trans) (1 PyrCO)" |
| 124,"1.0","266","false","39","(3 3PGDH) (-6 Acetate_Trans) (6 ACN) (6 ACS) (12 AdenylatK) (6 ADSL) (6 AICARTF) (6 AIRC) (6 AIRS) (6 CitS) (4 FBP) (-4 FBPAld) (6 FGAMS) (6 FUM) (3 GAPDH) (6 GARS) (6 GARTF) (6 Gln_Trans) (13 GlPDH_Q) (27 GluDH) (-18 GluS) (-3 GlyHMTrans) (3 GlyS) (6 ICitDH) (6 IMPCyc) (6 MDH) (30 Nitrogen_Trans) (3 PGK) (6 PPAT) (6 PRPPS) (3 PSAT) (3 PSerP) (4 R5PI) (-4 RPE) (6 SCAIRS) (-2 TA) (-2 TK1) (-2 TK2) (9 TPI)" |
| 125,"1.0","272","false","41","(3 3PGDH) (6 ACN) (6 AdenylatK) (6 ADSL) (6 AICARTF) (6 AIRC) (6 AIRS) (6 CitS) (4 FBP) (-4 FBPAld) (6 FGAMS) (6 FUM) (3 GAPDH) (6 GARS) (6 GARTF) (6 Gln_Trans) (13 GlPDH_Q) (27 GluDH) (-18 GluS) (-3 GlyHMTrans) (3 GlyS) (6 ICitDH) (6 IMPCyc) (6 MDH) (30 Nitrogen_Trans) (3 PGK) (6 PPAT) (6 PRPPS) (3 PSAT) (3 PSerP) (6 PyrDH_E1A) (6 PyrDH_E1B) (-6 PyrDH_E2) (6 PyrDH_E3) (4 R5PI) (-4 RPE) (6 SCAIRS) (-2 TA) (-2 TK1) (-2 TK2) (9 TPI)" |
| 126,"1.0","278","false","41","(3 3PGDH) (-6 Acetate_Trans) (6 ACN) (6 ACS) (12 AdenylatK) (6 ADSL) (6 AICARTF) (6 AIRC) (6 AIRS) (6 CitS) (4 ENO) (6 FGAMS) (6 FUM) (7 GAPDH) (6 GARS) (6 GARTF) (4 GlcPTS) (6 Gln_Trans) (9 GlPDH_Q) (27 GluDH) (-18 GluS) (-3 GlyHMTrans) (3 GlyS) (6 ICitDH) (6 IMPCyc) (6 MDH) (30 Nitrogen_Trans) (4 PGI) (7 PGK) (4 PGM) (6 PPAT) (6 PRPPS) (3 PSAT) (3 PSerP) (4 R5PI) (-4 RPE) (6 SCAIRS) (-2 TA) (-2 TK1) (-2 TK2) (9 TPI)" |
| 127,"1.0","284","false","43","(3 3PGDH) (6 ACN) (6 AdenylatK) (6 ADSL) (6 AICARTF) (6 AIRC) (6 AIRS) (6 CitS) (4 ENO) (6 FGAMS) (6 FUM) (7 GAPDH) (6 GARS) (6 GARTF) (4 GlcPTS) (6 Gln_Trans) (9 GlPDH_Q) (27 GluDH) (-18 GluS) (-3 GlyHMTrans) (3 GlyS) (6 ICitDH) (6 IMPCyc) (6 MDH) (30 Nitrogen_Trans) (4 PGI) (7 PGK) (4 PGM) (6 PPAT) (6 PRPPS) (3 PSAT) (3 PSerP) (6 PyrDH_E1A) (6 PyrDH_E1B) (-6 PyrDH_E2) (6 PyrDH_E3) (4 R5PI) (-4 RPE) (6 SCAIRS) (-2 TA) (-2 TK1) (-2 TK2) (9 TPI)" |
| 128,"1.0","260","false","40","(3 3PGDH) (6 ACN) (6 AdenylatK) (6 ADSL) (6 AICARTF) (6 AIRC) (6 AIRS) (6 AldDH) (6 CitS) (6 EthanolamineAmmoniaLya) (4 FBP) (-4 FBPAld) (6 FGAMS) (6 FUM) (3 GAPDH) (6 GARS) (6 GARTF) (6 Gln_Trans) (13 GlPDH_Q) (27 GluDH) (-18 GluS) (6 GlycerophosphodiesterPdisterase) (-3 GlyHMTrans) (3 GlyS) (6 ICitDH) (6 IMPCyc) (6 MDH) (24 Nitrogen_Trans) (3 PGK) (6 PPAT) (6 PRPPS) (3 PSAT) (3 PSerP) (4 R5PI) (-4 RPE) (6 SCAIRS) (-2 TA) (-2 TK1) (-2 TK2) (9 TPI)" |
| 129,"1.0","272","false","42","(3 3PGDH) (6 ACN) (6 AdenylatK) (6 ADSL) (6 AICARTF) (6 AIRC) (6 AIRS) (6 AldDH) (6 CitS) (4 ENO) (6 EthanolamineAmmoniaLya) (6 FGAMS) (6 FUM) (7 GAPDH) (6 GARS) (6 GARTF) (4 GlcPTS) (6 Gln_Trans) (9 GlPDH_Q) (27 GluDH) (-18 GluS) (6 GlycerophosphodiesterPdisterase) (-3 GlyHMTrans) (3 GlyS) (6 ICitDH) (6 IMPCyc) (6 MDH) (24 Nitrogen_Trans) (4 PGI) (7 PGK) (4 PGM) (6 PPAT) (6 PRPPS) (3 PSAT) (3 PSerP) (4 R5PI) (-4 RPE) (6 SCAIRS) (-2 TA) (-2 TK1) (-2 TK2) (9 TPI)" |
| 130,"1.0","2","false","2","(1 Glycerol_Trans) (1 GlycerolK)" |
| 131,"1.0","12","false","12","(1 Acetate_Trans) (1 AspK) (-1 AspSemiAldDH) (1 AspTA) (1 Cit_Trans) (-1 CitS) (1 GluDH) (1 HomoCysTmet) (-1 HomoSerDH) (1 HSerTAc) (1 Met_Trans) (1 Nitrogen_Trans)" |
| 132,"1.0","12","false","12","(1 ACS) (1 AdenylatK) (1 AspK) (-1 AspSemiAldDH) (1 AspTA) (1 GluDH) (1 HomoCysTmet) (-1 HomoSerDH) (1 HSerTAc) (1 Met_Trans) (1 Nitrogen_Trans) (1 PyrCO)" |
| 133,"1.0","15","false","15","(1 Acetate_Trans) (1 AspK) (-1 AspSemiAldDH) (1 AspTA) (1 GluDH) (1 HomoCysTmet) (-1 HomoSerDH) (1 HSerTAc) (1 Met_Trans) (1 Nitrogen_Trans) (1 PyrCO) (1 PyrDH_E1A) (1 PyrDH_E1B) (-1 PyrDH_E2) (1 PyrDH_E3)" |
| 134,"1.0","13","false","13","(1 Acetate_Trans) (1 AldDH) (1 AspK) (-1 AspSemiAldDH) (1 AspTA) (1 EthanolamineAmmoniaLya) (1 GluDH) (1 GlycerophosphodiesterPdisterase) (1 HomoCysTmet) (-1 HomoSerDH) (1 HSerTAc) (1 Met_Trans) (1 PyrCO)" |
| 135,"1.0","260","false","41","(3 3PGDH) (6 ACS) (12 AdenylatK) (6 ADSL) (6 AICARTF) (6 AIRC) (6 AIRS) (6 AspK) (-6 AspSemiAldDH) (6 AspTA) (4 FBP) (-4 FBPAld) (6 FGAMS) (6 FUM) (3 GAPDH) (6 GARS) (6 GARTF) (13 GlPDH_Q) (21 GluDH) (-12 GluS) (-3 GlyHMTrans) (3 GlyS) (6 HomoCysTmet) (-6 HomoSerDH) (6 HSerTAc) (6 IMPCyc) (6 MDH) (6 Met_Trans) (24 Nitrogen_Trans) (3 PGK) (6 PPAT) (6 PRPPS) (3 PSAT) (3 PSerP) (4 R5PI) (-4 RPE) (6 SCAIRS) (-2 TA) (-2 TK1) (-2 TK2) (9 TPI)" |
| 136,"1.0","278","false","45","(3 3PGDH) (6 Acetate_Trans) (6 AdenylatK) (6 ADSL) (6 AICARTF) (6 AIRC) (6 AIRS) (6 AspK) (-6 AspSemiAldDH) (6 AspTA) (4 FBP) (-4 FBPAld) (6 FGAMS) (6 FUM) (3 GAPDH) (6 GARS) (6 GARTF) (13 GlPDH_Q) (21 GluDH) (-12 GluS) (-3 GlyHMTrans) (3 GlyS) (6 HomoCysTmet) (-6 HomoSerDH) (6 HSerTAc) (6 IMPCyc) (6 MDH) (6 Met_Trans) (24 Nitrogen_Trans) (3 PGK) (6 PPAT) (6 PRPPS) (3 PSAT) (3 PSerP) (6 PyrDH_E1A) (6 PyrDH_E1B) (-6 PyrDH_E2) (6 PyrDH_E3) (4 R5PI) (-4 RPE) (6 SCAIRS) (-2 TA) (-2 TK1) (-2 TK2) (9 TPI)" |
| 137,"1.0","272","false","43","(3 3PGDH) (6 ACS) (12 AdenylatK) (6 ADSL) (6 AICARTF) (6 AIRC) (6 AIRS) (6 AspK) (-6 AspSemiAldDH) (6 AspTA) (4 ENO) (6 FGAMS) (6 FUM) (7 GAPDH) (6 GARS) (6 GARTF) (4 GlcPTS) (9 GlPDH_Q) (21 GluDH) (-12 GluS) (-3 GlyHMTrans) (3 GlyS) (6 HomoCysTmet) (-6 HomoSerDH) (6 HSerTAc) (6 IMPCyc) (6 MDH) (6 Met_Trans) (24 Nitrogen_Trans) (4 PGI) (7 PGK) (4 PGM) (6 PPAT) (6 PRPPS) (3 PSAT) (3 PSerP) (4 R5PI) (-4 RPE) (6 SCAIRS) (-2 TA) (-2 TK1) (-2 TK2) (9 TPI)" |
| 138,"1.0","290","false","47","(3 3PGDH) (6 Acetate_Trans) (6 AdenylatK) (6 ADSL) (6 AICARTF) (6 AIRC) (6 AIRS) (6 AspK) (-6 AspSemiAldDH) (6 AspTA) (4 ENO) (6 FGAMS) (6 FUM) (7 GAPDH) (6 GARS) (6 GARTF) (4 GlcPTS) (9 GlPDH_Q) (21 GluDH) (-12 GluS) (-3 GlyHMTrans) (3 GlyS) (6 HomoCysTmet) (-6 HomoSerDH) (6 HSerTAc) (6 IMPCyc) (6 MDH) (6 Met_Trans) (24 Nitrogen_Trans) (4 PGI) (7 PGK) (4 PGM) (6 PPAT) (6 PRPPS) (3 PSAT) (3 PSerP) (6 PyrDH_E1A) (6 PyrDH_E1B) (-6 PyrDH_E2) (6 PyrDH_E3) (4 R5PI) (-4 RPE) (6 SCAIRS) (-2 TA) (-2 TK1) (-2 TK2) (9 TPI)" |
| 139,"1.0","266","false","44","(3 3PGDH) (6 Acetate_Trans) (6 AdenylatK) (6 ADSL) (6 AICARTF) (6 AIRC) (6 AIRS) (6 AldDH) (6 AspK) (-6 AspSemiAldDH) (6 AspTA) (6 EthanolamineAmmoniaLya) (4 FBP) (-4 FBPAld) (6 FGAMS) (6 FUM) (3 GAPDH) (6 GARS) (6 GARTF) (13 GlPDH_Q) (21 GluDH) (-12 GluS) (6 GlycerophosphodiesterPdisterase) (-3 GlyHMTrans) (3 GlyS) (6 HomoCysTmet) (-6 HomoSerDH) (6 HSerTAc) (6 IMPCyc) (6 MDH) (6 Met_Trans) (18 Nitrogen_Trans) (3 PGK) (6 PPAT) (6 PRPPS) (3 PSAT) (3 PSerP) (4 R5PI) (-4 RPE) (6 SCAIRS) (-2 TA) (-2 TK1) (-2 TK2) (9 TPI)" |
| 140,"1.0","278","false","46","(3 3PGDH) (6 Acetate_Trans) (6 AdenylatK) (6 ADSL) (6 AICARTF) (6 AIRC) (6 AIRS) (6 AldDH) (6 AspK) (-6 AspSemiAldDH) (6 AspTA) (4 ENO) (6 EthanolamineAmmoniaLya) (6 FGAMS) (6 FUM) (7 GAPDH) (6 GARS) (6 GARTF) (4 GlcPTS) (9 GlPDH_Q) (21 GluDH) (-12 GluS) (6 GlycerophosphodiesterPdisterase) (-3 GlyHMTrans) (3 GlyS) (6 HomoCysTmet) (-6 HomoSerDH) (6 HSerTAc) (6 IMPCyc) (6 MDH) (6 Met_Trans) (18 Nitrogen_Trans) (4 PGI) (7 PGK) (4 PGM) (6 PPAT) (6 PRPPS) (3 PSAT) (3 PSerP) (4 R5PI) (-4 RPE) (6 SCAIRS) (-2 TA) (-2 TK1) (-2 TK2) (9 TPI)" |
| 141,"1.0","7","false","7","(1 ENO) (1 GAPDH) (1 GlPDH_Q) (1 PGK) (1 PGM) (1 PyrK) (1 TPI)" |
| 142,"1.0","60","false","21","(3 3PGDH) (2 Acetate_Trans) (2 ACN) (2 Asp_Trans) (2 AspTA) (4 Cit_Trans) (-2 CitS) (2 CysS) (3 GAPDH) (3 GlPDH_Q) (2 GluCysLig) (7 GluDH) (-1 GlyHMTrans) (1 GlyS) (2 ICitDH) (8 Nitrogen_Trans) (3 PGK) (3 PSAT) (3 PSerP) (2 SerTAc) (3 TPI)" |
| 143,"1.0","70","false","26","(3 3PGDH) (2 Acetate_Trans) (2 ACN) (2 AspK) (-2 AspSemiAldDH) (2 AspTA) (4 Cit_Trans) (-2 CitS) (2 CysS) (3 GAPDH) (3 GlPDH_Q) (2 GluCysLig) (7 GluDH) (-1 GlyHMTrans) (1 GlyS) (-2 HomoSerDH) (2 HomoSerK) (2 ICitDH) (8 Nitrogen_Trans) (3 PGK) (3 PSAT) (3 PSerP) (2 SerTAc) (2 Thr_Trans) (2 ThrS) (3 TPI)" |
| 144,"1.0","80","false","30","(3 3PGDH) (2 Acetate_Trans) (2 AcLacS2) (2 ACN) (2 AspK) (-2 AspSemiAldDH) (2 AspTA) (4 Cit_Trans) (-2 CitS) (2 CysS) (3 GAPDH) (3 GlPDH_Q) (2 GluCysLig) (9 GluDH) (-1 GlyHMTrans) (1 GlyS) (-2 HomoSerDH) (2 HomoSerK) (2 ICitDH) (2 Ile_EC) (2 Ile_Trans) (8 Nitrogen_Trans) (3 PGK) (3 PSAT) (3 PSerP) (2 SerTAc) (2 TA_B_Ile) (2 ThrDHA) (2 ThrS) (3 TPI)" |
| 145,"1.0","50","false","19","(3 3PGDH) (2 ACN) (2 ACS) (2 AdenylatK) (2 Cit_Trans) (2 CysS) (3 GAPDH) (3 GlPDH_Q) (2 GluCysLig) (5 GluDH) (-1 GlyHMTrans) (1 GlyS) (2 ICitDH) (6 Nitrogen_Trans) (3 PGK) (3 PSAT) (3 PSerP) (2 SerTAc) (3 TPI)" |
| 146,"1.0","56","false","22","(3 3PGDH) (2 Acetate_Trans) (2 ACN) (2 Cit_Trans) (2 CysS) (3 GAPDH) (3 GlPDH_Q) (2 GluCysLig) (5 GluDH) (-1 GlyHMTrans) (1 GlyS) (2 ICitDH) (6 Nitrogen_Trans) (3 PGK) (3 PSAT) (3 PSerP) (2 PyrDH_E1A) (2 PyrDH_E1B) (-2 PyrDH_E2) (2 PyrDH_E3) (2 SerTAc) (3 TPI)" |
| 147,"1.0","60","false","23","(3 3PGDH) (2 Acetate_Trans) (2 ACN) (4 Cit_Trans) (-2 CitS) (2 CysS) (-2 FUM) (3 GAPDH) (3 GlPDH_Q) (2 GluCysLig) (5 GluDH) (-1 GlyHMTrans) (1 GlyS) (2 ICitDH) (-2 MDH) (6 Nitrogen_Trans) (3 PGK) (3 PSAT) (3 PSerP) (2 SerTAc) (2 Succ_Trans) (-2 SuccDH) (3 TPI)" |
| 148,"1.0","52","false","21","(3 3PGDH) (2 Acetate_Trans) (2 ACN) (2 AldDH) (2 Cit_Trans) (2 CysS) (2 EthanolamineAmmoniaLya) (3 GAPDH) (3 GlPDH_Q) (2 GluCysLig) (5 GluDH) (2 GlycerophosphodiesterPdisterase) (-1 GlyHMTrans) (1 GlyS) (2 ICitDH) (4 Nitrogen_Trans) (3 PGK) (3 PSAT) (3 PSerP) (2 SerTAc) (3 TPI)" |
| 149,"1.0","181","false","41","(6 3PGDH) (-3 Acetate_Trans) (3 ACN) (6 ACS) (9 AdenylatK) (3 ADSL) (3 AICARTF) (3 AIRC) (3 AIRS) (3 CitS) (3 CysS) (2 FBP) (-2 FBPAld) (3 FGAMS) (3 FUM) (6 GAPDH) (3 GARS) (3 GARTF) (11 GlPDH_Q) (3 GluCysLig) (15 GluDH) (-6 GluS) (-3 GlyHMTrans) (3 GlyS) (3 ICitDH) (3 IMPCyc) (3 MDH) (18 Nitrogen_Trans) (6 PGK) (3 PPAT) (3 PRPPS) (6 PSAT) (6 PSerP) (2 R5PI) (-2 RPE) (3 SCAIRS) (3 SerTAc) (-1 TA) (-1 TK1) (-1 TK2) (9 TPI)" |
| 150,"1.0","193","false","44","(6 3PGDH) (3 Acetate_Trans) (3 ACN) (3 AdenylatK) (3 ADSL) (3 AICARTF) (3 AIRC) (3 AIRS) (3 CitS) (3 CysS) (2 FBP) (-2 FBPAld) (3 FGAMS) (3 FUM) (6 GAPDH) (3 GARS) (3 GARTF) (11 GlPDH_Q) (3 GluCysLig) (15 GluDH) (-6 GluS) (-3 GlyHMTrans) (3 GlyS) (3 ICitDH) (3 IMPCyc) (3 MDH) (18 Nitrogen_Trans) (6 PGK) (3 PPAT) (3 PRPPS) (6 PSAT) (6 PSerP) (6 PyrDH_E1A) (6 PyrDH_E1B) (-6 PyrDH_E2) (6 PyrDH_E3) (2 R5PI) (-2 RPE) (3 SCAIRS) (3 SerTAc) (-1 TA) (-1 TK1) (-1 TK2) (9 TPI)" |
| 151,"1.0","187","false","43","(6 3PGDH) (-3 Acetate_Trans) (3 ACN) (6 ACS) (9 AdenylatK) (3 ADSL) (3 AICARTF) (3 AIRC) (3 AIRS) (3 CitS) (3 CysS) (2 ENO) (3 FGAMS) (3 FUM) (8 GAPDH) (3 GARS) (3 GARTF) (2 GlcPTS) (9 GlPDH_Q) (3 GluCysLig) (15 GluDH) (-6 GluS) (-3 GlyHMTrans) (3 GlyS) (3 ICitDH) (3 IMPCyc) (3 MDH) (18 Nitrogen_Trans) (2 PGI) (8 PGK) (2 PGM) (3 PPAT) (3 PRPPS) (6 PSAT) (6 PSerP) (2 R5PI) (-2 RPE) (3 SCAIRS) (3 SerTAc) (-1 TA) (-1 TK1) (-1 TK2) (9 TPI)" |
| 152,"1.0","199","false","46","(6 3PGDH) (3 Acetate_Trans) (3 ACN) (3 AdenylatK) (3 ADSL) (3 AICARTF) (3 AIRC) (3 AIRS) (3 CitS) (3 CysS) (2 ENO) (3 FGAMS) (3 FUM) (8 GAPDH) (3 GARS) (3 GARTF) (2 GlcPTS) (9 GlPDH_Q) (3 GluCysLig) (15 GluDH) (-6 GluS) (-3 GlyHMTrans) (3 GlyS) (3 ICitDH) (3 IMPCyc) (3 MDH) (18 Nitrogen_Trans) (2 PGI) (8 PGK) (2 PGM) (3 PPAT) (3 PRPPS) (6 PSAT) (6 PSerP) (6 PyrDH_E1A) (6 PyrDH_E1B) (-6 PyrDH_E2) (6 PyrDH_E3) (2 R5PI) (-2 RPE) (3 SCAIRS) (3 SerTAc) (-1 TA) (-1 TK1) (-1 TK2) (9 TPI)" |
| 153,"1.0","181","false","43","(6 3PGDH) (3 Acetate_Trans) (3 ACN) (3 AdenylatK) (3 ADSL) (3 AICARTF) (3 AIRC) (3 AIRS) (6 AldDH) (3 CitS) (3 CysS) (6 EthanolamineAmmoniaLya) (2 FBP) (-2 FBPAld) (3 FGAMS) (3 FUM) (6 GAPDH) (3 GARS) (3 GARTF) (11 GlPDH_Q) (3 GluCysLig) (15 GluDH) (-6 GluS) (6 GlycerophosphodiesterPdisterase) (-3 GlyHMTrans) (3 GlyS) (3 ICitDH) (3 IMPCyc) (3 MDH) (12 Nitrogen_Trans) (6 PGK) (3 PPAT) (3 PRPPS) (6 PSAT) (6 PSerP) (2 R5PI) (-2 RPE) (3 SCAIRS) (3 SerTAc) (-1 TA) (-1 TK1) (-1 TK2) (9 TPI)" |
| 154,"1.0","187","false","45","(6 3PGDH) (3 Acetate_Trans) (3 ACN) (3 AdenylatK) (3 ADSL) (3 AICARTF) (3 AIRC) (3 AIRS) (6 AldDH) (3 CitS) (3 CysS) (2 ENO) (6 EthanolamineAmmoniaLya) (3 FGAMS) (3 FUM) (8 GAPDH) (3 GARS) (3 GARTF) (2 GlcPTS) (9 GlPDH_Q) (3 GluCysLig) (15 GluDH) (-6 GluS) (6 GlycerophosphodiesterPdisterase) (-3 GlyHMTrans) (3 GlyS) (3 ICitDH) (3 IMPCyc) (3 MDH) (12 Nitrogen_Trans) (2 PGI) (8 PGK) (2 PGM) (3 PPAT) (3 PRPPS) (6 PSAT) (6 PSerP) (2 R5PI) (-2 RPE) (3 SCAIRS) (3 SerTAc) (-1 TA) (-1 TK1) (-1 TK2) (9 TPI)" |
| 155,"1.0","58","false","21","(3 3PGDH) (-2 Acetate_Trans) (2 ACN) (4 ACS) (4 AdenylatK) (2 CitS) (2 CysS) (3 GAPDH) (3 GlPDH_Q) (2 GluCysLig) (5 GluDH) (-1 GlyHMTrans) (1 GlyS) (2 ICitDH) (6 Nitrogen_Trans) (3 PGK) (3 PSAT) (3 PSerP) (2 PyrCO) (2 SerTAc) (3 TPI)" |
| 156,"1.0","66","false","23","(3 3PGDH) (2 Acetate_Trans) (2 ACN) (2 CitS) (2 CysS) (3 GAPDH) (3 GlPDH_Q) (2 GluCysLig) (5 GluDH) (-1 GlyHMTrans) (1 GlyS) (2 ICitDH) (6 Nitrogen_Trans) (3 PGK) (3 PSAT) (3 PSerP) (2 PyrCO) (4 PyrDH_E1A) (4 PyrDH_E1B) (-4 PyrDH_E2) (4 PyrDH_E3) (2 SerTAc) (3 TPI)" |
| 157,"1.0","58","false","22","(3 3PGDH) (2 Acetate_Trans) (2 ACN) (4 AldDH) (2 CitS) (2 CysS) (4 EthanolamineAmmoniaLya) (3 GAPDH) (3 GlPDH_Q) (2 GluCysLig) (5 GluDH) (4 GlycerophosphodiesterPdisterase) (-1 GlyHMTrans) (1 GlyS) (2 ICitDH) (2 Nitrogen_Trans) (3 PGK) (3 PSAT) (3 PSerP) (2 PyrCO) (2 SerTAc) (3 TPI)" |
| 158,"1.0","56","false","21","(3 3PGDH) (2 Acetate_Trans) (2 ACN) (4 Cit_Trans) (-2 CitS) (2 CysS) (3 GAPDH) (3 GlPDH_Q) (2 GluCysLig) (5 GluDH) (-1 GlyHMTrans) (1 GlyS) (2 ICitDH) (2 Mae) (-2 MDH) (6 Nitrogen_Trans) (3 PGK) (3 PSAT) (3 PSerP) (2 SerTAc) (3 TPI)" |
| 159,"1.0","70","false","24","(3 3PGDH) (2 Acetate_Trans) (2 ACN) (2 AdenylatK) (2 Asn_Trans) (2 AsnS) (2 AspTA) (4 Cit_Trans) (-2 CitS) (2 CysS) (3 GAPDH) (3 GlPDH_Q) (2 GluCysLig) (9 GluDH) (-2 GluS) (-1 GlyHMTrans) (1 GlyS) (2 ICitDH) (10 Nitrogen_Trans) (3 PGK) (3 PSAT) (3 PSerP) (2 SerTAc) (3 TPI)" |
| 160,"1.0","47","false","32","(1 3PGDH) (1 AdenylatK) (1 AnthranS) (1 ChorS) (1 DAHPS) (1 DHQDA) (1 DHQS) (2 ENO) (1 EPSPS) (1 FBP) (-1 FBPAld) (3 GAPDH) (5 GlPDH_Q) (2 GluDH) (-1 GluS) (1 IndoS) (2 Nitrogen_Trans) (3 PGK) (2 PGM) (1 PRAIso) (1 PRPPS) (1 PRT) (1 PSAT) (1 PSerP) (1 R5PI) (-1 RPE) (-1 ShiDH) (1 ShiK) (-1 TK2) (4 TPI) (1 Trp_Trans) (1 TrpS)" |
| 161,"1.0","50","false","32","(1 3PGDH) (1 AdenylatK) (1 AnthranS) (1 ChorS) (1 DAHPS) (1 DHQDA) (1 DHQS) (3 ENO) (1 EPSPS) (4 GAPDH) (1 GlcPTS) (4 GlPDH_Q) (2 GluDH) (-1 GluS) (1 IndoS) (2 Nitrogen_Trans) (1 PGI) (4 PGK) (3 PGM) (1 PRAIso) (1 PRPPS) (1 PRT) (1 PSAT) (1 PSerP) (1 R5PI) (-1 RPE) (-1 ShiDH) (1 ShiK) (-1 TK2) (4 TPI) (1 Trp_Trans) (1 TrpS)" |
| 162,"1.0","14","false","12","(1 3PGDH) (1 GAPDH) (1 GlPDH_Q) (1 GluDH) (2 Gly_Trans) (-1 GlyHMTrans) (1 GlyS) (2 Nitrogen_Trans) (1 PGK) (1 PSAT) (1 PSerP) (1 TPI)" |
| 163,"1.0","8","false","8","(1 AdenylatK) (1 AspTA) (1 ASuccLya) (1 ASuccS) (1 FUM) (1 GluDH) (1 MDH) (1 Nitrogen_Trans)" |

**Table S2: Key enzymes occurring in all the flux modes.**

Flux modes occurred more than 6 times in the 20 knockouts tested which a) affect enzymatic activities and b) are capable to reduce intracellular growth.

| **Key enzyme (processes)** | **Freqs** | **Key enzyme (processes)** | **Freqs** | **Key enzyme (processes)** | **Freqs** |
| --- | --- | --- | --- | --- | --- |
| PGM | 42 | AICARTF | 42 | PPAT | 42 |
| FGAMS | 42 | AspSemiAldDH | 42 | PTA | 42 |
| MDH | 42 | SCAIRS | 42 | ThrS | 42 |
| ChorM | 42 | ADSL | 42 | DHQDA | 42 |
| HomoSerK | 42 | RPE | 42 | FBP | 36 |
| PSAT | 42 | PRPPS | 42 | PPDK | 30 |
| AspTA | 42 | AldDH | 42 | GluS | 28 |
| AspK | 42 | AdenylatK | 42 | GluDH | 28 |
| Ile_EC | 42 | GARS | 42 | GlnS | 28 |
| 3PGDH | 42 | IMPCyc | 42 | TPI | 24 |
| AIRS | 42 | ThrDHA | 42 | PGK | 24 |
| Ile_Trans | 42 | DAHPS | 42 | GAPDH | 24 |
| FUM | 42 | PSerP | 42 | GlPDH_Q | 24 |
| TA_B_Ile | 42 | GlycerophosphodiesterPdisterase | 42 | Phe_Trans | 21 |
| Acetate_Trans | 42 | DHQS | 42 | PheTA | 21 |
| EthanolamineAmmoniaLya | 42 | ChorS | 42 | PrephenateDA | 21 |
| EPSPS | 42 | AckA | 42 | PrephenateDH | 21 |
| HomoSerDH | 42 | ENO | 42 | Tyr_Trans | 21 |
| FBPAld | 42 | AIRC | 42 | TyrTA | 21 |
| AcLacS2 | 42 | ShiDH | 42 | FucI | 18 |
| ShiK | 42 | GlyS | 42 | Fucose_Trans | 18 |
| TK2 | 42 | R5PI | 42 | FucuK | 18 |
| GlyHMTrans | 42 | GARTF | 42 | FucuPAld | 18 |

**Table S3: Metabolic flux modes critical for cytoplasmic survival interpreted as pathway equations.** The degree of robustness for all the calculated elementary modes was studied. Considering all the 20-gene knockouts in our experiment, there are **57** elementary modes can not be altered through all these 20 mutants, there are **190** modes that can be only affected by a single gene deletion, **496** modes can be influenced by missing either of 2 genes, **994** modes can be blocked by any of 3 genes’ loss, **1332** modes by 4, **1499** modes by 5, **2818** modes by 6, **4698** modes by 7, 4628 by 8, 2629 by 9, 939 by 10, 342 by 11, 162 by 12. The most fragile **42** modes can be quenched by absence of any one of 13 genes, they are listed in the following tables. The corresponding pathways covers the common region of the metabolism system in our research, they reveal the critical pathways essentially important for the Listeria cytoplasmic survival.

| **K.o. genes** | **Modes in a equation view** |
| --- | --- |
| purE-ilvB-lmo0517-lmo2134-ilvC-glpD-serC-purS-purQ-lmo1235-aroB-eutB-ilvD | ADP + ATP + 4 FTHF + Glycerol3P + 10 Glycerophosphatidylethanolamine + 17 NAD+ + 15 NADPH + 2 Pyr + 11 Quinone = 10 Acetate_X + CO2 + 2 IMP + 2 Ile_X + 17 NADH + 15 NADP+ + 2 Phe_X + 11 Quinol + 4 THF |
| purE-ilvB-lmo0517-lmo2134-ilvC-glpD-serC-purS-purQ-lmo1235-aroB-eutB-ilvD | 28 ATP + 4 FTHF + 10 Glycerophosphatidylethanolamine + 8 NAD+ + 15 NADPH + 11 Pyr + 2 Quinone = 17 ADP + 10 Acetate_X + CO2 + 8 Glycerol3P + 2 IMP + 2 Ile_X + 8 NADH + 15 NADP+ + 2 Phe_X + 2 Quinol + 4 THF |
| purE-ilvB-lmo0517-lmo2134-ilvC-glpD-serC-purS-purQ-lmo1235-aroB-eutB-ilvD | 16 ATP + 4 FTHF + 10 Glycerophosphatidylethanolamine + 12 NAD+ + 15 NADPH + 7 Pyr + 6 Quinone = 9 ADP + 10 Acetate_X + CO2 + 4 Glycerol3P + 2 IMP + 2 Ile_X + 12 NADH + 15 NADP+ + 2 Phe_X + 6 Quinol + 4 THF |
| purE-ilvB-lmo0517-lmo2134-ilvC-glpD-serC-purS-purQ-lmo1235-aroB-eutB-ilvD | 26 ATP + 4 FTHF + 4 Glc_X + 10 Glycerophosphatidylethanolamine + 12 NAD+ + 15 NADPH + 7 Pyr + 2 Quinol = 15 ADP + 10 Acetate_X + CO2 + 12 Glycerol3P + 2 IMP + 2 Ile_X + 12 NADH + 15 NADP+ + 2 Phe_X + 2 Quinone + 4 THF |
| purE-ilvB-lmo0517-lmo2134-ilvC-glpD-serC-purS-purQ-lmo1235-aroB-eutB-ilvD | ADP + ATP + 4 FTHF + Glycerol3P + 10 Glycerophosphatidylethanolamine + 19 NAD+ + 15 NADPH + 2 Pyr + 11 Quinone = 10 Acetate_X + CO2 + 2 IMP + 2 Ile_X + 19 NADH + 15 NADP+ + 11 Quinol + 4 THF + 2 Tyr_X |
| purE-ilvB-lmo0517-lmo2134-ilvC-glpD-serC-purS-purQ-lmo1235-aroB-eutB-ilvD | 28 ATP + 4 FTHF + 10 Glycerophosphatidylethanolamine + 10 NAD+ + 15 NADPH + 11 Pyr + 2 Quinone = 17 ADP + 10 Acetate_X + CO2 + 8 Glycerol3P + 2 IMP + 2 Ile_X + 10 NADH + 15 NADP+ + 2 Quinol + 4 THF + 2 Tyr_X |
| purE-ilvB-lmo0517-lmo2134-ilvC-glpD-serC-purS-purQ-lmo1235-aroB-eutB-ilvD | 16 ATP + 4 FTHF + 10 Glycerophosphatidylethanolamine + 14 NAD+ + 15 NADPH + 7 Pyr + 6 Quinone = 9 ADP + 10 Acetate_X + CO2 + 4 Glycerol3P + 2 IMP + 2 Ile_X + 14 NADH + 15 NADP+ + 6 Quinol + 4 THF + 2 Tyr_X |
| purE-ilvB-lmo0517-lmo2134-ilvC-glpD-serC-purS-purQ-lmo1235-aroB-eutB-ilvD | 26 ATP + 4 FTHF + 4 Glc_X + 10 Glycerophosphatidylethanolamine + 14 NAD+ + 15 NADPH + 7 Pyr + 2 Quinol = 15 ADP + 10 Acetate_X + CO2 + 12 Glycerol3P + 2 IMP + 2 Ile_X + 14 NADH + 15 NADP+ + 2 Quinone + 4 THF + 2 Tyr_X |
| purE-ilvB-lmo0517-lmo2134-ilvC-serC-purS-purQ-lmo1235-aroB-eutB-lmo1031-ilvD | 12 ATP + 4 FTHF + 11 Fucose_X + 10 Glycerophosphatidylethanolamine + 17 NAD+ + 15 NADPH + 2 Pyr = 10 ADP + 10 Acetate_X + CO2 + 10 Glycerol3P + 2 IMP + 2 Ile_X + 11 LacAld + 17 NADH + 15 NADP+ + 2 Phe_X + 4 THF |
| purE-ilvB-lmo0517-lmo2134-ilvC-serC-purS-purQ-lmo1235-aroB-eutB-lmo1031-ilvD | 30 ATP + 4 FTHF + 2 Fucose_X + 10 Glycerophosphatidylethanolamine + 8 NAD+ + 15 NADPH + 11 Pyr = 19 ADP + 10 Acetate_X + CO2 + 10 Glycerol3P + 2 IMP + 2 Ile_X + 2 LacAld + 8 NADH + 15 NADP+ + 2 Phe_X + 4 THF |
| purE-ilvB-lmo0517-lmo2134-ilvC-serC-purS-purQ-lmo1235-aroB-eutB-lmo1031-ilvD | 22 ATP + 4 FTHF + 6 Fucose_X + 10 Glycerophosphatidylethanolamine + 12 NAD+ + 15 NADPH + 7 Pyr = 15 ADP + 10 Acetate_X + CO2 + 10 Glycerol3P + 2 IMP + 2 Ile_X + 6 LacAld + 12 NADH + 15 NADP+ + 2 Phe_X + 4 THF |
| purE-ilvB-lmo0517-lmo2134-ilvC-serC-purS-purQ-lmo1235-aroB-eutB-lmo1031-ilvD | 12 ATP + 4 FTHF + 11 Fucose_X + 10 Glycerophosphatidylethanolamine + 19 NAD+ + 15 NADPH + 2 Pyr = 10 ADP + 10 Acetate_X + CO2 + 10 Glycerol3P + 2 IMP + 2 Ile_X + 11 LacAld + 19 NADH + 15 NADP+ + 4 THF + 2 Tyr_X |
| purE-ilvB-lmo0517-lmo2134-ilvC-serC-purS-purQ-lmo1235-aroB-eutB-lmo1031-ilvD | 30 ATP + 4 FTHF + 2 Fucose_X + 10 Glycerophosphatidylethanolamine + 10 NAD+ + 15 NADPH + 11 Pyr = 19 ADP + 10 Acetate_X + CO2 + 10 Glycerol3P + 2 IMP + 2 Ile_X + 2 LacAld + 10 NADH + 15 NADP+ + 4 THF + 2 Tyr_X |
| purE-ilvB-lmo0517-lmo2134-ilvC-serC-purS-purQ-lmo1235-aroB-eutB-lmo1031-ilvD | 22 ATP + 4 FTHF + 6 Fucose_X + 10 Glycerophosphatidylethanolamine + 14 NAD+ + 15 NADPH + 7 Pyr = 15 ADP + 10 Acetate_X + CO2 + 10 Glycerol3P + 2 IMP + 2 Ile_X + 6 LacAld + 14 NADH + 15 NADP+ + 4 THF + 2 Tyr_X |
| purE-ilvB-lmo0517-lmo2134-ilvC-glpD-serC-purS-purQ-lmo1235-aroB-eutB-ilvD | 5 ATP + 4 FTHF + Glycerol3P + 10 Glycerophosphatidylethanolamine + 17 NAD+ + 15 NADPH + 2 Pyr + 11 Quinone = 3 ADP + 10 Acetate_X + CO2 + 2 IMP + 2 Ile_X + 17 NADH + 15 NADP+ + 2 Phe_X + 11 Quinol + 4 THF |
| purE-ilvB-lmo0517-lmo2134-ilvC-glpD-serC-purS-purQ-lmo1235-aroB-eutB-ilvD | 12 ATP + 4 FTHF + Glycerol3P + 10 Glycerophosphatidylethanolamine + 17 NAD+ + 15 NADPH + 2 Pyr + 11 Quinone = 10 ADP + 10 Acetate_X + CO2 + 2 IMP + 2 Ile_X + 17 NADH + 15 NADP+ + 2 Phe_X + 11 Quinol + 4 THF |
| purE-ilvB-lmo0517-lmo2134-ilvC-glpD-serC-purS-purQ-lmo1235-aroB-eutB-ilvD | 32 ATP + 4 FTHF + 10 Glycerophosphatidylethanolamine + 8 NAD+ + 15 NADPH + 11 Pyr + 2 Quinone = 21 ADP + 10 Acetate_X + CO2 + 8 Glycerol3P + 2 IMP + 2 Ile_X + 8 NADH + 15 NADP+ + 2 Phe_X + 2 Quinol + 4 THF |
| purE-ilvB-lmo0517-lmo2134-ilvC-glpD-serC-purS-purQ-lmo1235-aroB-eutB-ilvD | 39 ATP + 4 FTHF + 10 Glycerophosphatidylethanolamine + 8 NAD+ + 15 NADPH + 11 Pyr + 2 Quinone = 28 ADP + 10 Acetate_X + CO2 + 8 Glycerol3P + 2 IMP + 2 Ile_X + 8 NADH + 15 NADP+ + 2 Phe_X + 2 Quinol + 4 THF |
| purE-ilvB-lmo0517-lmo2134-ilvC-glpD-serC-purS-purQ-lmo1235-aroB-eutB-ilvD | 20 ATP + 4 FTHF + 10 Glycerophosphatidylethanolamine + 12 NAD+ + 15 NADPH + 7 Pyr + 6 Quinone = 13 ADP + 10 Acetate_X + CO2 + 4 Glycerol3P + 2 IMP + 2 Ile_X + 12 NADH + 15 NADP+ + 2 Phe_X + 6 Quinol + 4 THF |
| purE-ilvB-lmo0517-lmo2134-ilvC-glpD-serC-purS-purQ-lmo1235-aroB-eutB-ilvD | 27 ATP + 4 FTHF + 10 Glycerophosphatidylethanolamine + 12 NAD+ + 15 NADPH + 7 Pyr + 6 Quinone = 20 ADP + 10 Acetate_X + CO2 + 4 Glycerol3P + 2 IMP + 2 Ile_X + 12 NADH + 15 NADP+ + 2 Phe_X + 6 Quinol + 4 THF |
| purE-ilvB-lmo0517-lmo2134-ilvC-glpD-serC-purS-purQ-lmo1235-aroB-eutB-ilvD | 30 ATP + 4 FTHF + 4 Glc_X + 10 Glycerophosphatidylethanolamine + 12 NAD+ + 15 NADPH + 7 Pyr + 2 Quinol = 19 ADP + 10 Acetate_X + CO2 + 12 Glycerol3P + 2 IMP + 2 Ile_X + 12 NADH + 15 NADP+ + 2 Phe_X + 2 Quinone + 4 THF |
| purE-ilvB-lmo0517-lmo2134-ilvC-glpD-serC-purS-purQ-lmo1235-aroB-eutB-ilvD | 37 ATP + 4 FTHF + 4 Glc_X + 10 Glycerophosphatidylethanolamine + 12 NAD+ + 15 NADPH + 7 Pyr + 2 Quinol = 26 ADP + 10 Acetate_X + CO2 + 12 Glycerol3P + 2 IMP + 2 Ile_X + 12 NADH + 15 NADP+ + 2 Phe_X + 2 Quinone + 4 THF |
| purE-ilvB-lmo0517-lmo2134-ilvC-glpD-serC-purS-purQ-lmo1235-aroB-eutB-ilvD | 5 ATP + 4 FTHF + Glycerol3P + 10 Glycerophosphatidylethanolamine + 19 NAD+ + 15 NADPH + 2 Pyr + 11 Quinone = 3 ADP + 10 Acetate_X + CO2 + 2 IMP + 2 Ile_X + 19 NADH + 15 NADP+ + 11 Quinol + 4 THF + 2 Tyr_X |
| purE-ilvB-lmo0517-lmo2134-ilvC-glpD-serC-purS-purQ-lmo1235-aroB-eutB-ilvD | 12 ATP + 4 FTHF + Glycerol3P + 10 Glycerophosphatidylethanolamine + 19 NAD+ + 15 NADPH + 2 Pyr + 11 Quinone = 10 ADP + 10 Acetate_X + CO2 + 2 IMP + 2 Ile_X + 19 NADH + 15 NADP+ + 11 Quinol + 4 THF + 2 Tyr_X |
| purE-ilvB-lmo0517-lmo2134-ilvC-glpD-serC-purS-purQ-lmo1235-aroB-eutB-ilvD | 32 ATP + 4 FTHF + 10 Glycerophosphatidylethanolamine + 10 NAD+ + 15 NADPH + 11 Pyr + 2 Quinone = 21 ADP + 10 Acetate_X + CO2 + 8 Glycerol3P + 2 IMP + 2 Ile_X + 10 NADH + 15 NADP+ + 2 Quinol + 4 THF + 2 Tyr_X |
| purE-ilvB-lmo0517-lmo2134-ilvC-glpD-serC-purS-purQ-lmo1235-aroB-eutB-ilvD | 39 ATP + 4 FTHF + 10 Glycerophosphatidylethanolamine + 10 NAD+ + 15 NADPH + 11 Pyr + 2 Quinone = 28 ADP + 10 Acetate_X + CO2 + 8 Glycerol3P + 2 IMP + 2 Ile_X + 10 NADH + 15 NADP+ + 2 Quinol + 4 THF + 2 Tyr_X |
| purE-ilvB-lmo0517-lmo2134-ilvC-glpD-serC-purS-purQ-lmo1235-aroB-eutB-ilvD | 20 ATP + 4 FTHF + 10 Glycerophosphatidylethanolamine + 14 NAD+ + 15 NADPH + 7 Pyr + 6 Quinone = 13 ADP + 10 Acetate_X + CO2 + 4 Glycerol3P + 2 IMP + 2 Ile_X + 14 NADH + 15 NADP+ + 6 Quinol + 4 THF + 2 Tyr_X |
| purE-ilvB-lmo0517-lmo2134-ilvC-glpD-serC-purS-purQ-lmo1235-aroB-eutB-ilvD | 27 ATP + 4 FTHF + 10 Glycerophosphatidylethanolamine + 14 NAD+ + 15 NADPH + 7 Pyr + 6 Quinone = 20 ADP + 10 Acetate_X + CO2 + 4 Glycerol3P + 2 IMP + 2 Ile_X + 14 NADH + 15 NADP+ + 6 Quinol + 4 THF + 2 Tyr_X |
| purE-ilvB-lmo0517-lmo2134-ilvC-glpD-serC-purS-purQ-lmo1235-aroB-eutB-ilvD | 30 ATP + 4 FTHF + 4 Glc_X + 10 Glycerophosphatidylethanolamine + 14 NAD+ + 15 NADPH + 7 Pyr + 2 Quinol = 19 ADP + 10 Acetate_X + CO2 + 12 Glycerol3P + 2 IMP + 2 Ile_X + 14 NADH + 15 NADP+ + 2 Quinone + 4 THF + 2 Tyr_X |
| purE-ilvB-lmo0517-lmo2134-ilvC-glpD-serC-purS-purQ-lmo1235-aroB-eutB-ilvD | 37 ATP + 4 FTHF + 4 Glc_X + 10 Glycerophosphatidylethanolamine + 14 NAD+ + 15 NADPH + 7 Pyr + 2 Quinol = 26 ADP + 10 Acetate_X + CO2 + 12 Glycerol3P + 2 IMP + 2 Ile_X + 14 NADH + 15 NADP+ + 2 Quinone + 4 THF + 2 Tyr_X |
| purE-ilvB-lmo0517-lmo2134-ilvC-serC-purS-purQ-lmo1235-aroB-eutB-lmo1031-ilvD | 16 ATP + 4 FTHF + 11 Fucose_X + 10 Glycerophosphatidylethanolamine + 17 NAD+ + 15 NADPH + 2 Pyr = 14 ADP + 10 Acetate_X + CO2 + 10 Glycerol3P + 2 IMP + 2 Ile_X + 11 LacAld + 17 NADH + 15 NADP+ + 2 Phe_X + 4 THF |
| purE-ilvB-lmo0517-lmo2134-ilvC-serC-purS-purQ-lmo1235-aroB-eutB-lmo1031-ilvD | 23 ATP + 4 FTHF + 11 Fucose_X + 10 Glycerophosphatidylethanolamine + 17 NAD+ + 15 NADPH + 2 Pyr = 21 ADP + 10 Acetate_X + CO2 + 10 Glycerol3P + 2 IMP + 2 Ile_X + 11 LacAld + 17 NADH + 15 NADP+ + 2 Phe_X + 4 THF |
| purE-ilvB-lmo0517-lmo2134-ilvC-serC-purS-purQ-lmo1235-aroB-eutB-lmo1031-ilvD | 34 ATP + 4 FTHF + 2 Fucose_X + 10 Glycerophosphatidylethanolamine + 8 NAD+ + 15 NADPH + 11 Pyr = 23 ADP + 10 Acetate_X + CO2 + 10 Glycerol3P + 2 IMP + 2 Ile_X + 2 LacAld + 8 NADH + 15 NADP+ + 2 Phe_X + 4 THF |
| purE-ilvB-lmo0517-lmo2134-ilvC-serC-purS-purQ-lmo1235-aroB-eutB-lmo1031-ilvD | 41 ATP + 4 FTHF + 2 Fucose_X + 10 Glycerophosphatidylethanolamine + 8 NAD+ + 15 NADPH + 11 Pyr = 30 ADP + 10 Acetate_X + CO2 + 10 Glycerol3P + 2 IMP + 2 Ile_X + 2 LacAld + 8 NADH + 15 NADP+ + 2 Phe_X + 4 THF |
| purE-ilvB-lmo0517-lmo2134-ilvC-serC-purS-purQ-lmo1235-aroB-eutB-lmo1031-ilvD | 26 ATP + 4 FTHF + 6 Fucose_X + 10 Glycerophosphatidylethanolamine + 12 NAD+ + 15 NADPH + 7 Pyr = 19 ADP + 10 Acetate_X + CO2 + 10 Glycerol3P + 2 IMP + 2 Ile_X + 6 LacAld + 12 NADH + 15 NADP+ + 2 Phe_X + 4 THF |
| purE-ilvB-lmo0517-lmo2134-ilvC-serC-purS-purQ-lmo1235-aroB-eutB-lmo1031-ilvD | 33 ATP + 4 FTHF + 6 Fucose_X + 10 Glycerophosphatidylethanolamine + 12 NAD+ + 15 NADPH + 7 Pyr = 26 ADP + 10 Acetate_X + CO2 + 10 Glycerol3P + 2 IMP + 2 Ile_X + 6 LacAld + 12 NADH + 15 NADP+ + 2 Phe_X + 4 THF |
| purE-ilvB-lmo0517-lmo2134-ilvC-serC-purS-purQ-lmo1235-aroB-eutB-lmo1031-ilvD | 16 ATP + 4 FTHF + 11 Fucose_X + 10 Glycerophosphatidylethanolamine + 19 NAD+ + 15 NADPH + 2 Pyr = 14 ADP + 10 Acetate_X + CO2 + 10 Glycerol3P + 2 IMP + 2 Ile_X + 11 LacAld + 19 NADH + 15 NADP+ + 4 THF + 2 Tyr_X |
| purE-ilvB-lmo0517-lmo2134-ilvC-serC-purS-purQ-lmo1235-aroB-eutB-lmo1031-ilvD | 23 ATP + 4 FTHF + 11 Fucose_X + 10 Glycerophosphatidylethanolamine + 19 NAD+ + 15 NADPH + 2 Pyr = 21 ADP + 10 Acetate_X + CO2 + 10 Glycerol3P + 2 IMP + 2 Ile_X + 11 LacAld + 19 NADH + 15 NADP+ + 4 THF + 2 Tyr_X |
| purE-ilvB-lmo0517-lmo2134-ilvC-serC-purS-purQ-lmo1235-aroB-eutB-lmo1031-ilvD | 34 ATP + 4 FTHF + 2 Fucose_X + 10 Glycerophosphatidylethanolamine + 10 NAD+ + 15 NADPH + 11 Pyr = 23 ADP + 10 Acetate_X + CO2 + 10 Glycerol3P + 2 IMP + 2 Ile_X + 2 LacAld + 10 NADH + 15 NADP+ + 4 THF + 2 Tyr_X |
| purE-ilvB-lmo0517-lmo2134-ilvC-serC-purS-purQ-lmo1235-aroB-eutB-lmo1031-ilvD | 41 ATP + 4 FTHF + 2 Fucose_X + 10 Glycerophosphatidylethanolamine + 10 NAD+ + 15 NADPH + 11 Pyr = 30 ADP + 10 Acetate_X + CO2 + 10 Glycerol3P + 2 IMP + 2 Ile_X + 2 LacAld + 10 NADH + 15 NADP+ + 4 THF + 2 Tyr_X |
| purE-ilvB-lmo0517-lmo2134-ilvC-serC-purS-purQ-lmo1235-aroB-eutB-lmo1031-ilvD | 26 ATP + 4 FTHF + 6 Fucose_X + 10 Glycerophosphatidylethanolamine + 14 NAD+ + 15 NADPH + 7 Pyr = 19 ADP + 10 Acetate_X + CO2 + 10 Glycerol3P + 2 IMP + 2 Ile_X + 6 LacAld + 14 NADH + 15 NADP+ + 4 THF + 2 Tyr_X |
| purE-ilvB-lmo0517-lmo2134-ilvC-serC-purS-purQ-lmo1235-aroB-eutB-lmo1031-ilvD | 33 ATP + 4 FTHF + 6 Fucose_X + 10 Glycerophosphatidylethanolamine + 14 NAD+ + 15 NADPH + 7 Pyr = 26 ADP + 10 Acetate_X + CO2 + 10 Glycerol3P + 2 IMP + 2 Ile_X + 6 LacAld + 14 NADH + 15 NADP+ + 4 THF + 2 Tyr_X |

**Figure S1. Elementary mode numbers affected by cytoplasmically attenuated mutants.**

Shown are results of a flux analysis based on elementary mode analysis (EMA).


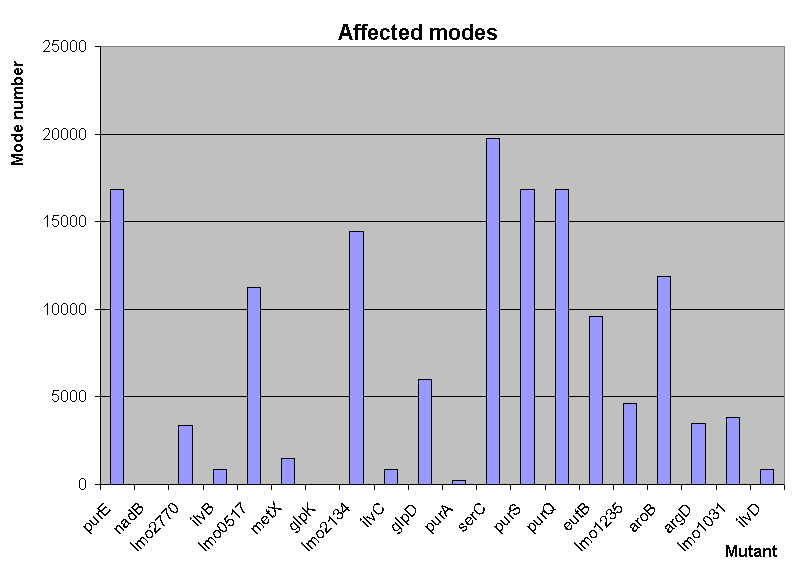

Supplement: Additional file 4 — Results obtained from the knockout in silico experiment are summarized in this document (Tables S1-S3, Figure S1). Table S1: Metabolic flux modes calculated using extreme pathway analysis. Table S2: Key enzymes occurring in all the flux modes. Table S3: Metabolic flux modes critical for cytoplasmic survival interpreted as pathway equations. Figure S1: Elementary mode numbers affected by cytoplasmically attenuated mutants. [file 1471-2164-11-573-S4.DOC]
