# Supplementary material for: Deciphering the intracellular metabolism of Listeria monocytogenes by mutant screening and modelling
Source: BMC Genomics. 2010 Oct 18;11:573. doi: 10.1186/1471-2164-11-573 (PMC3091722; doi:10.1186/1471-2164-11-573)
Supplement: Additional file 5 — Oligonucleotides used in this study. [file 1471-2164-11-573-S5.DOC]

Additional file 5: Oligonucleotides used in this study.

**construction of mutant EGD*∆*lmo1031-1036**

Lmo1030A GCCTGCTGTTGGTTGC

Lmo1030B GAAGATCTCCCAATTTTAATCGCCTC

Nested1030AB CGGGATCCGATAAAACGAATGGTTG

Lmo1037C GAAGATCTCTCCTTTTTTGAAAGATAGC

Lmo1037D GTCATGGAGTTATCCAGAG

Nested1037CD GGAATTCCGTGCCCCTTTATAGC

**construction of mutant EGD*∆*lmo0135-37**

Lmo0135A GGAGTAGAGGTTGGCG

Lmo0135B GAAGATCTTAATAAAAATTTCTTCATCTAAC

Lmo0135NestedAB CGGGATCCAACTGGGGAAGATATG

Lmo0137C GAAGATCTGACGTGCTGAGAAAAGG

Lmo0137D CTGGATCCGCTTGCTGGCTC

Lmo0137NestedCD GGAATTCAAGCTCTTGGTCTTTG

**construction of mutant EGD*∆*lmo2734**

Lmo2734A GACTCGCAGCAGAC

Lmo2734B GAAGATCTTCGGAATGTGCAACAACATG

Nested2734AB CGGGATCCCGCCAATCGGAC

Lmo2734C GAAGATCTTCGGAAAGGATTTGCAACTATTTTG

Lmo2734D CGAATTGGCTGTCCTTC

Nested2734CD GTAGGAATTCATTCAATG

**construction of mutant EGD*∆*lmo2781**

Lmo2783A CCGATTGTATATAACTTGAG

Lmo2782B GAAGATCTCTTATCCAAATAAACAGCC

Nested2783AB CGGAATTCCGACACCAGTGGTTGC

Lmo2780C GAAGATCTACTAAATTAACAGGGCGAG

Lmo2779D TCAATCGTCGCAAACGG

Nested2779CD CGGGATCCTACCAGCTGTAAGTGCC

**construction of mutant EGD*∆*lmo1085**

Lmo1085A GGAAGTAAACGTGGATG

Lmo1085B GAAGATCTTCCGACAATTATACGAATGCATC

Nested1085AB GGGGATCCCGCAGTACAGATTATGTTTTCG

Lmo1085C GAAGATCTTCGCAGCTCGAATTATTGATTATG

Lmo1085D GCCACGAACTGGAATATCTG

Nested1085CD CGGAATTCCGCAGGGATAACGGTATC

**construction of mutant EGD*∆tagB***

Lmo1087A CTGTAGCGTTTCATGC

Lmo1087B GAAGATCTTCGCTATATTTTTCACTATC

Lmo1087NestedAB CGGGATCCCGGTGTATGGGGCGATGG

Lmo1090C GAAGATCTTCGCAAGTGAACGCATGG

Lmo1090D GACGATGTAATCTCCCG

**construction of mutant EGD*∆*lmo0190**

Lmo0189A GGTATAGACTTAATCC

Lmo0189B GAAGATCTTGCAATGGTTTTTGGCATTTATTTTC

Lmo0189NestedAB CGGGATCCGTTAGCCCTTCCAACTG

Lmo0191C GAAGATCTAGTGAAGGCGAGAACG

Lmo0191D GACTACGTCATTCGTCC

Lmo0191NestedCD CGGAATTCTCGAATGCTCACACCG

**construction of mutant EGD*∆*lmo0618**

Lmo0619A GCGAATCATTCGTTTGGTC

Lmo0619B GAAGATCTAAGTGTCATTTCTCCCATTTATC

Lmo0619NestedAB CGGGATCCTGGTCTAGCGTATAACC

Lmo0617C GAAGATCTTCCTGTGTATCTATTTATG

Lmo0617D GCCGCAAACAATCATTG

Lmo0617NestedCD CGGAATTCCGCCCATACTAGC

**construction of mutant EGD*∆*lmo1506**

Lmo1507A GCATCAAGTAGCTCATC

Lmo1507B GAAGATCTTCTCTTAAAAAAATTCATGTG

Lmo1507NestedAB CGGGATCCCATAGTCATCTGCGCC

Lmo1505C GAAGATCTATGAATCCAAAAACAATTC

Lmo1505D CCATAATAACGCATTTAC

Lmo1505NestedCD CGGAATTCCAGTCGGCTCATCAGC

**construction of mutant EGD*∆oppF***

Lmo2193A GGGATGCGTCAACGG

Lmo2193B GAAGATCTTTCTCTTTGTTCAGTCATTC

Lmo2193NestedAB CGGGATCCGTAACCATCCAAGCGC

Lmo2191C GAAGATCTCATGCTAAGTTAACTGCTG

Lmo2191D CAATAATGATAGGACGTC

Lmo2191NestedCD CGGAATTCATCCTCAGTCATACGAAG
